# A one-pot, multicomponent reaction for the synthesis of novel 2-alkyl substituted 4-aminoimidazo[1,2-*a*][1,3,5]triazines†‡

**DOI:** 10.1039/c8ra03703e

**Published:** 2018-06-12

**Authors:** Felicia Phei Lin Lim, Lin Yuing Tan, Edward R. T. Tiekink, Anton V. Dolzhenko

**Affiliations:** School of Pharmacy, Monash University Malaysia Jalan Lagoon Selatan, Bandar Sunway Selangor Darul Ehsan 47500 Malaysia dolzhenkoav@gmail.com anton.dolzhenko@monash.edu +60-3-5514-6364 +60-3-5514-5867; Research Centre for Crystalline Materials, School of Science and Technology, Sunway University Bandar Sunway Selangor Darul Ehsan 47500 Malaysia; School of Pharmacy and Biomedical Sciences, Curtin Health Innovation Research Institute, Faculty of Health Sciences, Curtin University GPO Box U1987 Perth Western Australia 6845 Australia

## Abstract

A highly selective, one-pot, three-component synthesis of novel 2-alkyl-substituted 4-aminoimidazo[1,2-*a*][1,3,5]triazines has been developed. The scope of the method was explored in two dimensions, varying the structures of trialkyl orthoesters and 2-aminoimidazoles in their reactions with cyanamide. Conveniently performed under microwave irradiation, this method was also proved to be efficient under conventional heating. A library of 24 novel compounds was prepared in high purity using this multicomponent approach. Molecular and crystal structures of representative molecules were studied using X-ray crystallography.

## Abstract 2

A highly selective, one-pot, three-component synthesis of novel 2-alkyl-substituted 4-aminoimidazo[1,2-*a*][1,3,5]triazines has been developed.

## Graphical abstract

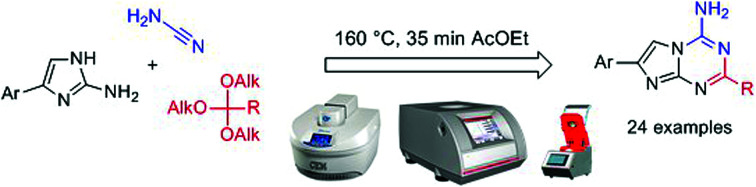

## Introduction

The imidazo[1,2-*a*][1,3,5]triazine ring system is recognised as an important scaffold in medicinal chemistry.^2^ The well-defined targets for imidazo[1,2-*a*][1,3,5]triazines include a variety of enzymes, such as focal adhesion kinase,^3^ activated Cdc42-associated tyrosine kinase 1,^4^ dipeptidyl peptidase IV,^5^ and phosphodiesterase 5.^6^ This scaffold has been successfully used for the design of ligands for μ-opioid^7^ and A_1_ adenosine^8^ receptors. The pharmacological potential of imidazo[1,2-*a*][1,3,5]triazines as anti-cancer,^9^ anti-viral^10^ and anti-diabetic^11^ agents has also been well documented in the literature. The broad spectrum of biological activities of imidazo[1,2-*a*][1,3,5]triazines results in ongoing demand for the development of new practical methods for the synthesis of diversely functionalized imidazo[1,2-*a*][1,3,5]triazines.

Since the first report^12^ on the synthesis of the imidazo[1,2-*a*][1,3,5]triazine ring system 50 years ago, a number of methods for the preparation of compounds with this heterocyclic core have been reported. They typically involve triazine ring construction onto substituted imidazoles^13^ or, more often, the annulation of the imidazole ring onto substituted 1,3,5-triazines.^14^ The two most common approaches utilize: (1) reactions of 2-amino-1,3,5-triazines with α-haloaldehydes/ketones^3,8,12,14*a*,*b*^ or chalcones,^14*c*^ and (2) intramolecular cyclisations of 2-amino-1,3,5-triazines substituted at the amino group.^11,14*d*–*f*^ Reports on the preparation of imidazo[1,2-*a*][1,3,5]triazines *via* the formation of the 1,3,5-triazine ring onto imidazoles are limited and usually utilize addition of 2-aminoimidazoles to *N*-acyliso(thio)cyanates followed by the intramolecular closure of the 1,3,5-triazine ring.^13^

Multicomponent reactions (MCRs) represent an efficient strategy in modern synthetic chemistry.^15^ Minimising the number of synthetic steps in obtaining targeted compounds from available starting reagents is highly desirable in the fields of organic synthesis and drug discovery. Due to their diversity and combinatorial potential, MCRs have become highly appreciated tools for the preparation of libraries of compounds for biological screening.^16^ However, issues surrounding chemo- and regio-selectivity of MCRs often pose a challenge and require fine-tuning during method development. The need for rapid and selective construction of biologically active compounds for drug discovery has stimulated intense development in the use of microwave technology. Microwave irradiation has been effectively used as an alternative source of energy, improving efficiency and selectivity of MCRs.^1,17,18^

We have been exploring the potential for new one-pot, multicomponent, microwave-assisted protocols for the synthesis of azolo[1,3,5]triazines.^18^ After the development of the MCRs utilizing aminoazoles, orthoformates and cyanamide, we have been refining the scope of this reaction using a variety of aminoazoles. In particular, the molecular diversity of compounds prepared has been achieved by varying the structures of the aminoazole precursors used in this three-component condensation and included libraries of pyrazolo[1,5-*a*][1,3,5]triazines,^1,18*a*–*c*^ 1,2,4-triazolo[1,5-*a*][1,3,5]triazines,^18*d*,*e*^ and imidazo[1,2-*a*][1,3,5]triazines.^18*f*^ Herein, we report our attempts to further expand the scope of this MCR by exploring its capacity to accommodate various orthoesters and therefore, introduce a new point of diversity in the reaction products. 2-Aminoimidazoles were selected as aminoazole substrates for the reaction in order to explore the regio- and chemo-selectivity of the process for these challenging substrates bearing endocyclic nitrogen atoms of similar reactivity.

## Results & discussion

### Synthesis

The starting materials used in our three-component reaction, *viz.* 2-aminoimidazoles (1), were prepared using the previously reported method developed by Van der Eycken's group.^19^ Optimization of the conditions for our proposed MCR was performed using a model reaction of 2-amino-4-phenylimidazole (1a), triethyl orthoacetate, and cyanamide under microwave irradiation (Table 1). The initial attempt to carry out this reaction at 150 °C for 20 min in ethyl acetate led to 4-amino-2-methyl-7-phenylimidazo[1,2-*a*][1,3,5]triazine (5a) obtained in 42% yield. Despite the moderate yield, the product 5a was isolated in a chromatography-free process as the sole product. We found that the yield could be improved to 60% by performing the reaction at 160 °C for 35 min. However, despite attempts to further increase the yields by manipulating the reaction conditions, no improvement in the outcome was achieved.

**Table 1:** Optimization of conditions for the model reaction^a^

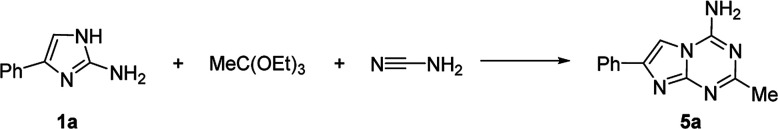			
Entry	Temperature (°C)	Time (min)	Isolated yield (%)
1	150	20	42
2	150	30	47
3	160	20	47
4	170	20	41
5	160	30	51
6	160	35	60
7	160	40	47
8^b^	160	35	45

a The reaction was performed using a Discover SP CEM microwave synthesizer with 1 mmol of 1a, 2.5 mmol of triethyl orthoacetate and 2.5 mmol of cyanamide in 2 mL of the ethyl acetate.

b The reaction was performed using 1 mmol of 1a, 3 mmol of triethyl orthoacetate and 3 mmol of cyanamide.

The developed method was tested using two different models of microwave synthesizers: Discover SP (CEM) and Monowave 400 (Anton Paar). The three-component reaction of 1a, triethyl orthoacetate and cyanamide under the optimized conditions was conducted in both systems and resulted in the formation of 5a in 60% and 56% yields, respectively.

Attempts to perform the reaction under reflux in ethyl acetate for 12 h led to the recovery of the starting material 1a without traces of 5a. However, an attempt to carry out the MCR of 1a, triethyl orthoacetate and cyanamide in the Monowave 50 (Anton Paar) reactor using sealed vessels under fast conventional heating imitating the optimised microwave irradiation conditions was successful, resulting in the 54% yield of equally pure 5a. Therefore, we could exclude any significant contribution of non-thermal microwave effects in promoting this MCR.

**Scheme 1:**
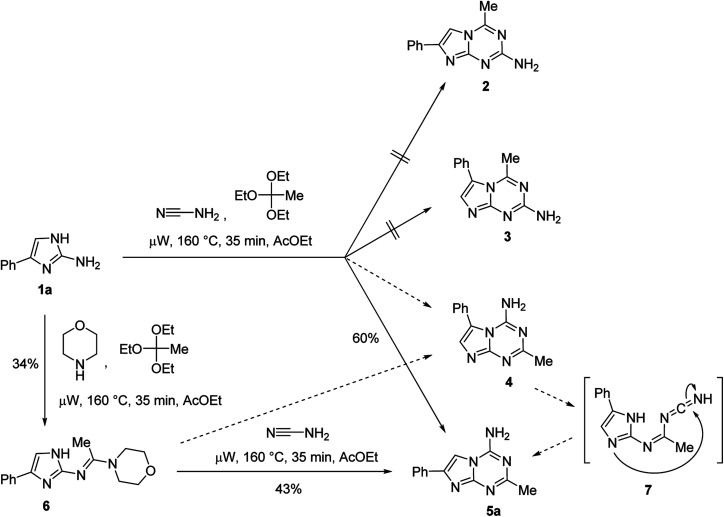
Synthesis of 4-amino-2-methyl-7-phenylimidazo[1,2-*a*][1,3,5]triazine (5a).

In principle, the annulation of the 1,3,5-triazine ring onto the 2-aminoimidazole 1a in the three-component reaction could proceed with the formation of four regioisomeric structures *viz.* 2-aminoimidazo[1,2-*a*][1,3,5]triazines 2 and 3 as well as 4-aminoimidazo[1,2-*a*][1,3,5]triazines 4 and 5a (Scheme 1). However, only one product was isolated from the MCR. The structure of this product was assigned on the basis of spectral data and an alternative synthesis of 5a adopting our previously reported step-wise synthesis of azolo[1,3,5]triazines *via* the 1,3,5-triazine ring annulation onto aminoazoles with a predisposed position of the amino group on the triazine ring.^20^ This method converted aminoazoles into formamidine intermediates *via* the reaction with orthoformates and morpholine followed by the triazine ring closing reaction of these intermediates with cyanamide. The conversion of 2-amino-4-phenylimidazole (1a) to the corresponding acetamidine (6) was achieved successfully using the reaction with triethyl orthoacetate and morpholine under microwave irradiation. The subsequent reaction of 6 with cyanamide led to the formation of a product identical to the one obtained from the MCR (Scheme 1). While the step-wise method excluded formation of compounds 2 and 3, a ring-closing reaction could still afford compounds 4 and/or 5a. The steric hindrance between the amino group and the phenyl ring in 4 would affect the thermodynamic stability of this potential product, therefore decreasing chances for its formation. Moreover, we could not exclude a rearrangement of 4 into 5a under the reaction conditions. The rearrangement would proceed *via* intermediate 7*via* the mechanism similar to the one reported^21^ for the rearrangement of another fused heterocycle with the amino-1,3,5-triazine ring. NOESY experiments performed on the product did not revealed any through-space interactions between the phenyl and methyl groups of the compound thus supporting assignment of structure 5a as the MCR product. X-ray crystal structure determinations for analogous compounds 5g and 5p (*vide infra*) further confirmed the regioselective closure of the triazine ring to the more sterically accessible endocyclic nitrogen of the imidazole.

Using the optimized conditions from the model reaction, we explored the scope of our three-component reaction using a variety of trialkyl orthoesters and 4-aryl substituted 2-aminoimidazoles (1). The method was successfully applied for the synthesis of a library of 24 new 2-alkyl-4-amino-7-arylimidazo[1,2-*a*][1,3,5]triazines (5) (Table 2). The reaction was found to proceed similarly for the full range of tested 2-aminoimidazoles accommodating aryl substituents in the position 4 of the imidazole ring. However, variations in the alkyl orthoester type affected product yields more significantly. In general, an increase in length of alkyl chain substituent on compound 5 led to a decrease of isolated yields. The yields of compounds 5a–f and 5g–l obtained from triethyl orthoacetate and triethyl orthopropionate, respectively, were substantially higher than those of 5m–r and 5s–x prepared using triethyl orthobutyrate and trimethyl orthovalerate. The decrease of yields in the homologous series of compounds 5 could be attributed to the relatively lower reactivity of long chain orthoesters used for their preparation. This assumption found a confirmation in the isolation of compounds 5 exclusively from all the reactions. No traces of other isomers 2–4 were detected. Besides products 5, only unreacted 2-aminoimidazoles (1) could be isolated from the reaction mixtures.

**Table 2:** Three-component synthesis of 2-alkyl-4-amino-7-arylimidazo[1,2-*a*][1,3,5]triazines (5)^a^

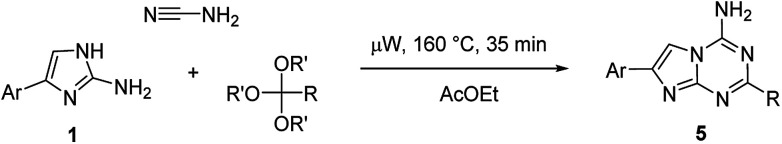
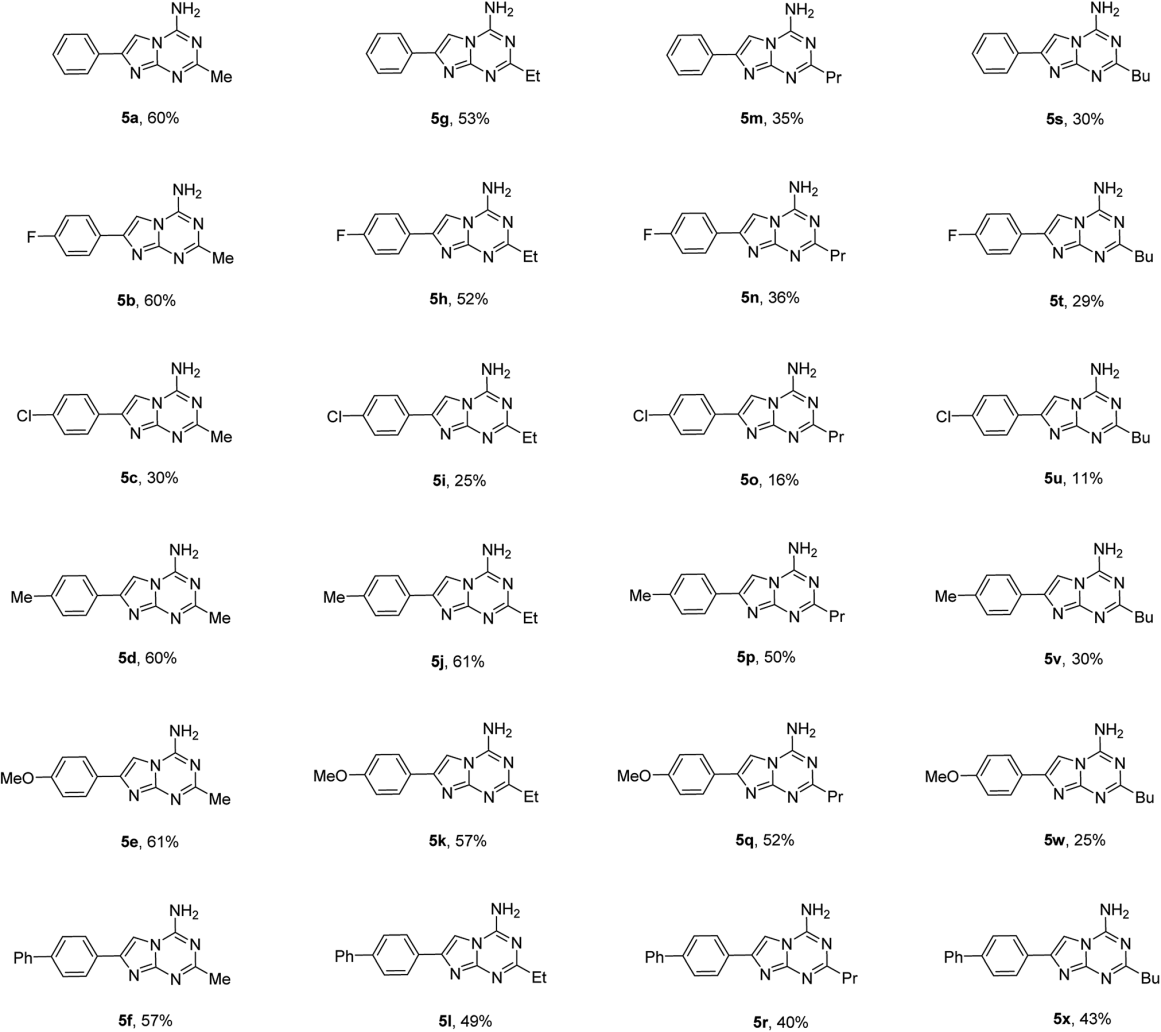

a The reaction was performed using a Discover SP CEM microwave synthesizer with 1 mmol of 2-aminoimidazoles (1), 2.5 mmol of trialkyl orthoesters and 2.5 mmol of cyanamide at 160 °C for 35 min in 2 mL of ethyl acetate.

### X-ray crystallography

**Fig. 1:**
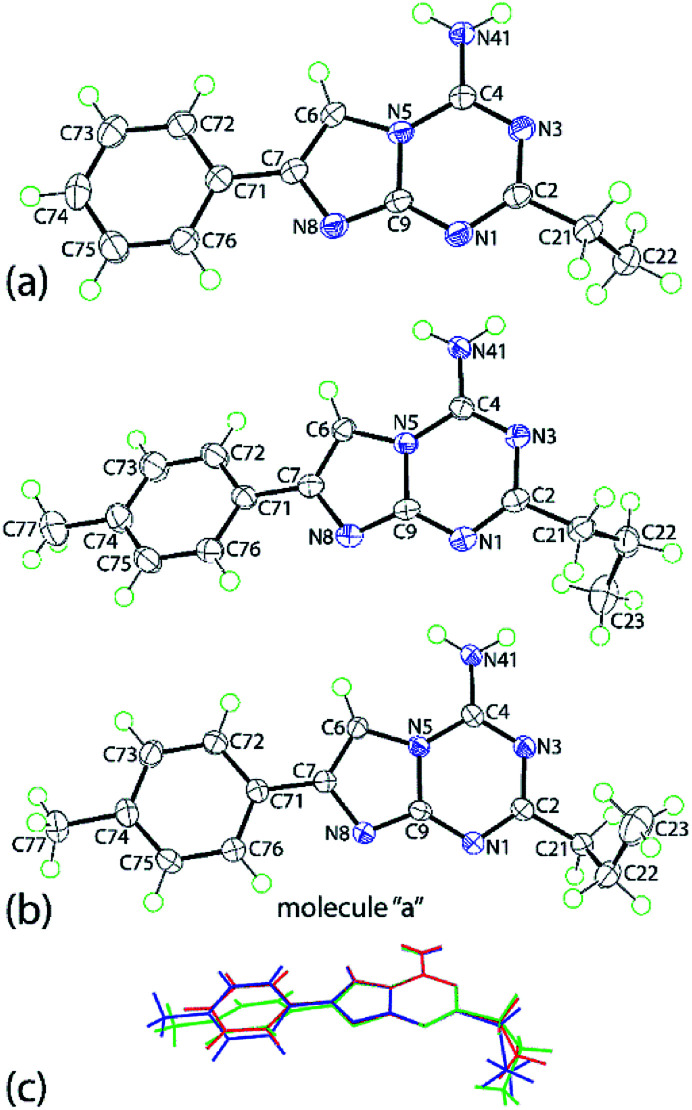
(a) Molecular structure of 5g and (b) molecular structures of the two independent molecules comprising the asymmetric unit of 5p, showing atom labelling scheme and 70% anisotropic displacement parameters. (c) An overlay diagram of 5g (red image), the first independent molecule of 5p (green) and the inverted second independent molecule of 5p (blue). The molecules have been overlapped so the triazine rings are coincident.

Crystals were obtained for two representative compounds, namely 5g and 5p, and their crystal and molecular structures determined by X-ray crystallography. The molecular structure of 5g is shown in Fig. 1a and comprises an essentially planar nine-membered ring with the r.m.s. deviation of the fitted atoms being 0.0129 Å; the maximum deviations above and below the least-square plane are found for the C2 [0.0215(8) Å] and C9 [0.0232(9) Å] atoms, respectively; the N41 and C71 atoms lie, respectively, 0.0464(13) and 0.0413(13) Å to the same side of the molecule.

Indeed, the entire molecule, with the exception of the C2-bound ethyl group, is essentially co-planar as seen in the dihedral between the imidazo[1,2-*a*][1,3,5]triazine and phenyl rings of 2.31(5)°. The ethyl group has an +anti-clinal (+ac) disposition with respect to the fused-ring system as seen in the torsion angle of 97.41(12)° for N1–C2–C21–C22.

The crystallographic asymmetric of 5p comprises two independent molecules, illustrated in Fig. 1b. The molecules present features that differ from those described for 5g. The r.m.s. of the nine atoms of the fused-ring system is 0.0247 Å [0.0103 Å for the second independent molecule] with the N41 and C71 atoms lying 0.0263(15) and 0.0837(14) Å [0.0333(15) and 0.0113(14) Å] to the same side of the plane. A twist in the molecule is evident as seen in the dihedral angle of 18.78(6)° [10.45(3)°] between the rings. The C2-bound *n*-propyl groups have very similar conformations with the C2–C21–C22–C23 torsion angles being −68.78(14) and −72.27(13)°, indicating a −syn-clinal (−sc) disposition in each case. The difference is that in the first independent molecule, the terminal methyl group is orientated away from the amino substituent whereas in the second molecule, it is orientated towards the amino group. An overlay diagram of the three molecules of 5g and 5p is shown in Fig. 1c. The difference in the co-planarity of the aromatic residues of 5g and 5p is clearly evident, as are the different orientations of the *n*-propyl substituents in 5p.

**Fig. 2:**
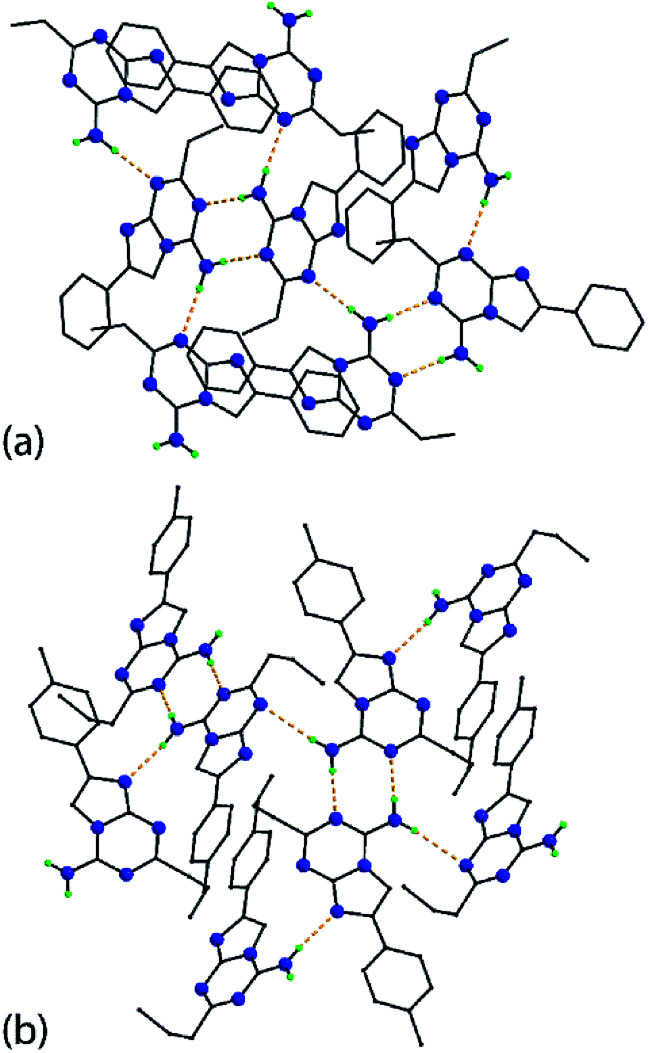
Supramolecular layers sustained by amino-N-H⋯N(ring) hydrogen bonding (see ESI Fig. S1‡), shown as orange dashed lines, in the crystals of (a) 5g and (b) 5p. Non-participating hydrogen atoms have been omitted and the carbon atoms are represented as small spheres.

The molecular packing arrangements in the crystals of 5g and 5p feature directional amino-N-H⋯N(ring) hydrogen bonding. In 5g, centrosymmetrically-related molecules are connected *via* amino-N-H⋯N3(triazine) hydrogen bonds to form eight-membered {⋯HNCN}_2_ synthons. The second amino-N-H hydrogen atom forms a hydrogen bond to the triazine-N1 atom. The result is a supramolecular layer parallel to (1 0 0) and with a zig-zag topology with the phenyl groups lying to either side, Fig. 2a. The layers stack along the *a*-axis with the primary interactions between them being of the type π⋯π. The closest such interactions [3.4699(6) Å] occur between imidazo rings, and between phenyl rings [3.8142(7) Å]. A view of the unit cell contents is shown in ESI Fig. S1‡ and geometric parameters characterizing the intermolecular interactions are given in the figure caption.

A supramolecular layer sustained by amino-N-H⋯N(ring) hydrogen bonding is also found in the crystal of 5p. The eight-membered {⋯HNCN}_2_ synthons arising from amino-N-H⋯N3(triazine) hydrogen bonds is formed by each of the independent molecules which self-associate. For the first independent molecule, rather than an amino-N-H⋯N3(triazine) hydrogen bond as formed by the second independent molecule (and in the crystal of 5g), an amino-N-H⋯N8(imidazo) hydrogen bond is formed instead; the latter pairs of interactions involve both independent molecules. As in 5g, the supramolecular layer, which is approximately parallel to (1 0 1), has a jagged topology with the 4-tolyl rings projecting to either side, Fig. 2b. The connections between layers to consolidate the three-dimensional architecture are of the type π(imidazo)⋯π(imidazo) [3.5695(7) Å] and π(triazine)⋯π(phenyl) [3.8946(7) and 3.9224(7) Å]. A view of the unit cell contents is shown in ESI Fig. S2‡ with geometric parameters given in the chart caption.

## Conclusion

In summary, we have successfully developed a three component reaction for the synthesis of novel 2-alkyl substituted 4-aminoimidazo[1,2-*a*][1,3,5]triazines 5 using easily accessible starting materials and a simple, catalyst- and chromatography-free protocol. The distinct advantage of the proposed approach was found to be high chemo- and regio-selectivity with the exclusive formation of desired products in high purity.

## Experimental section

### General information

Melting points (uncorrected) were determined on a Stuart™ SMP40 automatic melting point apparatus. ^1^H and ^13^C NMR spectra were recorded on a Bruker Fourier 300 spectrometer (300 MHz) using DMSO-*d*_6_ as a solvent and TMS as an internal reference. Microwave-assisted reactions were carried out in the closed vessel focused single mode using a Discover SP microwave synthesizer (CEM, USA) monitoring reaction temperature by equipped IR sensor. The model reaction was also carried out using Monowave 400 (Anton Paar, Austria) and Monowave 50 (Anton Paar, Austria) reactors.

#### General method for the synthesis of 4-amino-2-alkyl-7-arylimidazo[1,2-*a*][1,3,5]triazines (5) under microwave irradiation

The mixture of 2-aminoimidazoles (1, 1 mmol), cyanamide (105 mg, 2.5 mmol) and trialkyl orthoesters (2.5 mmol) in ethyl acetate (2 mL) were irradiated in a 10 mL seamless pressure vial using a microwave system operating at maximal microwave power up to 150 W (Discover SP, CEM) or 850 W (Monowave 400, Anton Paar) at 160 °C for 35 min. After cooling, the precipitate was filtered, washed with ethyl acetate and recrystallised from appropriate solvents.

##### 4-Amino-2-methyl-7-phenylimidazo[1,2-*a*][1,3,5]triazine (5a)

Light yellow solid, yield 60%, mp 303–305 °C (EtOH). ^1^H NMR (300 MHz, DMSO-*d*_6_): *δ* 2.38 (3H, s, CH_3_), 7.35 (1H, t, ^3^*J* = 7.4 Hz, H-4′), 7.47 (2H, t, ^3^*J* = 7.5 Hz, H-3′ and H-5′), 7.85 (2H, d, ^3^*J* = 7.0 Hz, H-2′ and H-6′), 8.15 (1H, s, H-6), 8.35 (2H, br s, NH_2_). ^13^C NMR (75 MHz, DMSO-*d*_6_): *δ* 25.3 (CH_3_), 101.7 (C-6), 125.2 (C-2′ and C-6′), 127.9 (C-4′), 128.7 (C-3′ and C-5′), 133.3 (C-1′), 143.2 (C-7), 150.3 & 150.6 (C-4 and C-8a), 164.7 (C-2). Anal. Calcd for C_12_H_11_N_5_: C, 63.99; H, 4.92; N, 31.09. Found: C, 63.82; H, 5.08; N, 30.91.

##### 4-Amino-2-methyl-7-(4-fluorophenyl)imidazo[1,2-*a*][1,3,5]triazine (5b)

White solid, yield 60%, mp > 350 °C (EtOH). ^1^H NMR (300 MHz, DMSO-*d*_6_): *δ* 2.38 (3H, s, CH_3_), 7.30 (2H, dd, ^3^*J*_HF_ = 8.9 Hz, ^3^*J*_HH_ = 8.9 Hz, H-3′ and H-5′), 7.87 (2H, dd, ^4^*J*_HF_ = 5.5 Hz, ^3^*J*_HH_ = 8.9 Hz, H-2′ and H-6′), 8.11 (1H, s, H-6), 8.37 (2H, br s, NH_2_). ^13^C NMR (75 MHz, DMSO-*d*_6_): *δ* 25.3 (CH_3_), 101.5 (C-6), 115.7 (d, ^2^*J*_CF_ = 21.7 Hz, C-3′ and C-5′), 127.2 (d, ^3^*J*_CF_ = 8.3 Hz, C-2′ and C-6′), 129.8 (d, ^4^*J*_CF_ = 2.9 Hz, C-1′), 142.3 (C-7), 150.4 & 150.6 (C-4 and C-8a), 161.9 (d, ^1^*J*_CF_ = 244.7 Hz, C-4′), 164.8 (C-2). Anal. Calcd for C_12_H_10_FN_5_: C, 59.25; H, 4.14; N, 28.79. Found: C, 59.02; H, 4.30; N, 28.63.

##### 4-Amino-2-methyl-7-(4-chlorophenyl)imidazo[1,2-*a*][1,3,5]triazine (5c)

Brown solid, yield 30%, mp > 350 °C (EtOH). ^1^H NMR (300 MHz, DMSO-*d*_6_): *δ* 2.37 (3H, s, CH_3_), 7.53 (2H, d, ^3^*J* = 8.6 Hz, H-3′ and H-5′), 7.9 (2H, d, ^3^*J* = 8.58 Hz, H-2′ and H-6′), 8.16 (1H, s, H-6), 8.38 (2H, br s, NH_2_). ^13^C NMR (75 MHz, DMSO-*d*_6_): *δ* 25.3 (CH_3_), 102.1 (C-6), 126.9 (C-2′ and C-6′), 128.8 (C-3′ and C-5′), 132.1 (C-1′), 132.3 (C-4′), 142.1 (C-7), 150.6 & 150.4 (C-4 and C-8a), 164.9 (C-2). Anal. Calcd for C_12_H_10_ClN_5_: C, 55.50; H, 3.88; N, 26.97. Found: 55.42; H, 4.01; N, 26.75.

##### 4-Amino-2-methyl-7-(4-methylphenyl)imidazo[1,2-*a*][1,3,5]triazine (5d)

White solid, yield 60%, mp > 350 °C (EtOH). ^1^H NMR (300 MHz, DMSO-*d*_6_): *δ* 2.34 (3H, s, CH_3_), 2.37 (3H, s, CH_3_), 7.27 (2H, d, ^3^*J* = 7.9 Hz, H-3′ and H-5′), 7.73 (2H, d, ^3^*J* = 8.1 Hz, H-2′ and H-6′), 8.09 (1H, s, H-6), 8.30 (2H, br s, NH_2_). ^13^C NMR (75 MHz, DMSO-*d*_6_): *δ* 20.8 (CH_3_), 25.3 (CH_3_), 101.1 (C-6), 125.2 (C-2′ and C-6′), 129.3 (C-3′ and C-5′), 130.5 (C-1′), 137.3 (C-4′), 143.4 (C-7), 150.2 & 150.5 (C-4 and C-8a), 164.5 (C-2). Anal. Calcd for C_13_H_13_N_5_: C, 65.25; H, 5.48; N, 29.27. Found: 65.18; H, 5.60; N, 29.18.

##### 4-Amino-2-methyl-7-(4-methoxyphenyl)imidazo[1,2-*a*][1,3,5]triazine (5e)

Light yellow solid, yield 61%, mp 338–339 °C (EtOH). ^1^H NMR (300 MHz, DMSO-*d*_6_): *δ* 2.37 (3H, s, CH_3_), 3.80 (3H, s, OCH_3_), 7.03 (2H, d, ^3^*J* = 8.9 Hz, H-3′ and H-5′), 7.77 (2H, d, ^3^*J* = 8.9 Hz, H-2′ and H-6′), 8.02 (1H, s, H-6), 8.29 (2H, br s, NH_2_). ^13^C NMR (75 MHz, DMSO-*d*_6_): *δ* 25.3 (CH_3_), 55.1 (OCH_3_), 100.4 (C-6), 114.2 (C-3′ and C-5′), 125.8 (C-1′), 126.6 (C-2′ and C-6′), 143.3 (C-7), 150.3 & 150.5 (C-4 and C-8a), 159.2 (C-4′), 164.4 (C-2). Anal. Calcd for C_13_H_13_N_5_O: C, 61.17; H, 5.13; N, 27.43. Found: C, 61.05; H, 5.23; N, 27.32.

##### 4-Amino-2-methyl-7-(4-biphenyl)imidazo[1,2-*a*][1,3,5]triazine (5f)

Light brown solid, yield 57%, mp > 350 °C (DMF). ^1^H NMR (300 MHz, DMSO-*d*_6_): *δ* 2.38 (3H, s, CH_3_), 7.38 (1H, t, ^3^*J* = 7.3 Hz, H-4′′), 7.49 (2H, t, ^3^*J* = 7.5 Hz, H-3′′ and H-5′′), 7.73 (2H, d, ^3^*J* = 7.1 Hz, H-2′′ and H-6′′), 7.79 (2H, d, ^3^*J* = 8.4 Hz, H-3′ and H-5′), 7.94 (2H, d, ^3^*J* = 8.4 Hz, H-2′ and H-6′), 8.20 (1H, s, H-6), 8.36 (2H, br s, NH_2_). ^13^C NMR (75 MHz, DMSO-*d*_6_): *δ* 25.3 (CH_3_), 101.8 (C-6), 125.8 (C-3′ and C-5′), 126.4 (C-3′′ and C-5′′), 127.0 (C-2′ and C-6′), 127.4 (C-4′′), 128.9 (C-2′′ and C-6′′), 132.4 (C-1′), 139.5 (C-4′ and C-1′′), 142.9 (C-7), 150.4 & 150.6 (C-4 and C-8a), 164.7 (C-2). Anal. Calcd for C_18_H_15_N_5_: C, 71.74; H, 5.02; N, 23.24. Found: C, 71.68; H, 5.11; N, 23.10.

##### 4-Amino-2-ethyl-7-phenylimidazo[1,2-*a*][1,3,5]triazine (5g)

Light yellow solid, yield 53%, mp 299–301 °C (EtOH). ^1^H NMR (300 MHz, DMSO-*d*_6_): *δ* 1.25 (3H, t, ^3^*J* = 7.6 Hz, CH_3_), 2.66 (2H, q, ^3^*J* = 7.5 Hz, CH_2_), 7.35 (1H, t, ^3^*J* = 7.4 Hz, H-4′), 7.47 (2H, t, ^3^*J* = 7.5 Hz, H-3′ and H-5′), 7.86 (2H, d, ^3^*J* = 7.0 Hz, H-2′ and H-6′), 8.16 (1H, s, H-6), 8.36 (2H, br s, NH_2_); ^13^C NMR (75 MHz, DMSO-*d*_6_): *δ* 11.7 (CH_3_), 31.5 (CH_2_), 101.7 (C-6), 125.2 (C-2′ and C-6′), 127.9 (C-4′), 128.7 (C-3′ and C-5′), 133.3 (C-1′), 143.3 (C-7), 150.4 & 150.8 (C-4 and C-8a), 168.6 (C-2). Anal. Calcd for C_13_H_13_N_5_: C, 65.25; H, 5.48; N, 29.27. Found: C, 65.22; H, 5.52; N, 29.12.

##### 4-Amino-2-ethyl-7-(4-fluorophenyl)imidazo[1,2-*a*][1,3,5]triazine (5h)

White solid, yield 52%, mp 339–340 °C (EtOH). ^1^H NMR (300 MHz, DMSO-*d*_6_): *δ* 1.25 (3H, t, ^3^*J* = 7.6 Hz, CH_3_), 2.65 (2H, q, ^3^*J* = 7.5 Hz, CH_2_), 7.31 (2H, dd, ^3^*J*_HF_ = 8.9 Hz, ^3^*J*_HH_ = 8.9 Hz, H-3′ and H-5′), 7.88 (2H, dd, ^4^*J*_HF_ = 5.5 Hz, ^3^*J*_HH_ = 8.9 Hz, H-2′ and H-6′), 8.12 (1H, s, H-6), 8.35 (2H, br s, NH_2_). ^13^C NMR (75 MHz, DMSO-*d*_6_): *δ* 11.7 (CH_3_), 31.5 (CH_2_), 101.5 (C-6), 115.7 (d, ^2^*J*_CF_ = 21.8 Hz, C-3′ and C-5′), 127.2 (d, ^3^*J*_CF_ = 8.3 Hz, C-2′ and C-6′), 129.9 (d, ^4^*J*_CF_ = 3.0 Hz, C-1′), 142.4 (C-7), 150.4 & 150.8 (C-4 and C-8a), 161.9 (d, ^1^*J*_CF_ = 245.1 Hz, C-4′), 168.7 (C-2). Anal. Calcd for C_13_H_12_FN_5_: C, 60.69; H, 4.70; N, 27.22. Found: C, 60.62; H, 4.83; N, 27.11.

##### 4-Amino-2-ethyl-7-(4-chlorophenyl)imidazo[1,2-*a*][1,3,5]triazine (5i)

Brown solid, yield 25%, mp 340–342 °C (EtOH).^1^H NMR (300 MHz, DMSO-*d*_6_): *δ* 1.24 (3H, t, ^3^*J* = 7.6 Hz, CH_3_), 2.65 (2H, q, ^3^*J* = 7.5 Hz, CH_2_), 7.53 (2H, d, ^3^*J* = 8.7 Hz, H-3′ and H-5′), 7.85 (2H, d, ^3^*J* = 8.7 Hz, H-2′ and H-6′), 8.17 (1H, s, H-6), 8.36 (2H, br s, NH_2_). ^13^C NMR (75 MHz, DMSO-*d*_6_): *δ* 11.7 (CH_3_), 31.5 (CH_2_), 102.1 (C-6), 126.9 (C-2′ and C-6′), 128.8 (C-3′ and C-5′), 132.2 (C-1′), 132.3 (C-4′), 142.1 (C-7), 150.5 & 150.8 (C-4 and C-8a), 168.8 (C-2). Anal. Calcd for C_13_H_12_ClN_5_: C, 57.04; H, 4.42; N, 25.59. Found: C, 56.97; H, 4.50; N, 25.49.

##### 4-Amino-2-ethyl-7-(4-methyphenyl)imidazo[1,2-*a*][1,3,5]triazine (5j)

Light yellow solid, yield 61%, mp > 350 °C (EtOH). ^1^H NMR (300 MHz, DMSO-*d*_6_): *δ* 1.25 (3H, t, ^3^*J* = 7.6 Hz, CH_3_), 2.34 (3H, s, CH_3_), 2.65 (2H, q, ^3^*J* = 7.5 Hz, CH_2_), 7.27 (2H, d, ^3^*J* = 8.0 Hz, H-3 and H-5), 7.75 (2H, d, ^3^*J* = 8.1 Hz, H-2′ and H-6′), 8.10 (1H, s, H-6), 8.31 (2H, br s, NH_2_). ^13^C NMR (75 MHz, DMSO-*d*_6_): *δ* 11.7 (CH_3_), 20.8 (CH_3_), 31.5 (CH_2_), 101.2 (C-6), 125.2 (C-2′ and C-6′), 129.3 (C-3′ and C-5′), 130.6 (C-1′), 137.3 (C-4′), 143.5 (C-7), 150.3 & 150.7 (C-4 and C-8a), 168.5 (C-2). Anal. Calcd for C_14_H_15_N_5_: C, 66.38; H, 5.97; N, 27.65. Found: C, 66.33; H, 6.08; N, 27.58.

##### 4-Amino-2-ethyl-7-(4-methoxyphenyl)imidazo[1,2-*a*][1,3,5]triazine (5k)

White solid, yield 57%, mp 328–330 °C (EtOH). ^1^H NMR (300 MHz, DMSO-*d*_6_): *δ* 1.24 (3H, t, ^3^*J* = 7.6 Hz, CH_3_), 2.64 (2H, q, ^3^*J* = 7.6 Hz, CH_2_), 3.80 (3H, s, OCH_3_), 7.04 (2H, d, ^3^*J* = 8.9 Hz, H-3′ and H-5′), 7.78 (2H, d, ^3^*J* = 8.9 Hz, H-2′ and H-6′), 8.02 (1H, s, H-6), 8.29 (2H, br s, NH_2_). ^13^C NMR (75 MHz, DMSO-*d*_6_): *δ* 11.7 (CH_3_), 31.4 (CH_2_), 100.4 (C-6), 114.2 (C-2′ and C-6′), 125.9 (C-4′), 126.6 (C-3′ and C-5′), 143.4 (C-7), 150.3 & 150.6 (C-4 and C-8a), 159.2 (C-1′), 168.3 (C-2). Anal. Calcd for C_14_H_15_N_5_O: C, 62.44; H, 5.61; N, 26.01. Found: C, 62.38; H, 5.74; N, 25.90.

##### 4-Amino-2-ethyl-7-(4-biphenyl)imidazo[1,2-*a*][1,3,5]triazine (5l)

White solid, yield 49%, mp 348–350 °C (DMF).^1^H NMR (300 MHz, DMSO-*d*_6_): *δ* 1.26 (3H, t, ^3^*J* = 7.6 Hz, CH_3_), 2.66 (2H, q, ^3^*J* = 7.6 Hz CH_2_), 7.38 (1H, t, ^3^*J* = 7.3 Hz, H-4′′), 7.49 (2H, t, ^3^*J* = 7.5 Hz, H-3′′ and H-5′′), 7.73 (2H, d, ^3^*J* = 7.2 Hz, H-2′′ and H-6′′), 7.79 (2H, d, ^3^*J* = 8.4 Hz, H-3′ and H-5′), 7.95 (2H, d, ^3^*J* = 8.3 Hz, H-2′ and H-6′), 8.21 (1H, s, H-6), 8.36 (2H, br s, NH_2_). ^13^C NMR (75 MHz, DMSO-*d*_6_): *δ* 11.7 (CH_3_), 31.5 (CH_2_), 101.9 (C-6), 125.8 (C-3′ and C-5′), 126.4 (C-3′′and C-5′′), 127.0 (C-2′ and C-6′), 127.4 (C-4′′), 128.9 (C-2′′ and C-6′′), 132.4 (C-1′), 139.5 & 139.6 (C-4′ and C-1′′), 143.0 (C-7), 150.5 & 150.8 (C-4 and C-8a), 168.7 (C-2). Anal. Calcd for C_19_H_17_N_5_: C, 72.36; H, 5.43; N, 22.21. Found: C, 72.31; H, 5.52; N, 22.14.

##### 4-Amino-2-propyl-7-phenylimidazo[1,2-*a*][1,3,5]triazine (5m)

Light yellow solid, yield 35%, mp 272–274 °C (EtOH). ^1^H NMR (300 MHz, DMSO-*d*_6_): *δ* 0.94 (3H, t, ^3^*J* = 7.4 Hz, CH_3_), 1.77 (2H, m, ^3^*J* = 7.4 Hz, CH_2_), 2.60 (2H, t, ^3^*J* = 7.4 Hz, CH_2_), 7.35 (1H, t, ^3^*J* = 7.3 Hz, H-4′), 7.47 (2H, t, ^3^*J* = 7.5 Hz, H-3′ and H-5′), 7.85 (2H, d, ^3^*J* = 7.1 Hz, H-2′ and H-6′), 8.15 (1H, s, H-6), 8.35 (2H, br s, NH_2_). ^13^C NMR (75 MHz, DMSO-*d*_6_): *δ* 13.6 (CH_3_), 20.4 (CH_2_), 40.2 (CH_2_), 101.7 (C-6), 125.2 (C-2′ and C-6′), 127.9 (C-4′), 128.7 (C-3′ and C-5′), 133.3 (C-1′), 143.3 (C-7), 150.3 & 150.8 (C-4 and C-8a), 167.6 (C-2). Anal. Calcd for C_14_H_15_N_5_: C, 66.38; H, 5.97; N, 27.65. Found: C, 66.31; H, 6.02; N, 27.60.

##### 4-Amino-2-propyl-7-(4-fluorophenyl)imidazo[1,2-*a*][1,3,5]triazine (5n)

White solid, yield 36%, mp 290–292 °C (EtOH). ^1^H NMR (300 MHz, DMSO-*d*_6_): *δ* 0.94 (3H, t, ^3^*J* = 7.4 Hz, CH_3_), 1.76 (2H, m, ^3^*J* = 7.4 Hz, CH_2_), 2.60 (2H, t, ^3^*J* = 7.4 Hz, CH_2_), 7.31 (2H, dd, ^3^*J*_HF_ = 8.9 Hz, ^3^*J*_HH_ = 8.9 Hz, H-3′ and H-5′), 7.87 (2H, dd, ^4^*J*_HF_ = 5.5 Hz, ^3^*J*_HH_ = 8.9 Hz, H-2′ and H-6′), 8.11 (1H, s, H-6), 8.34 (2H, br s, NH_2_). ^13^C NMR (75 MHz, DMSO-*d*_6_): *δ* 13.6 (CH_3_), 20.4 (CH_2_), 40.2 (CH_2_), 101.5 (C-6), 115.7 (d, ^2^*J*_CF_ = 21.7 Hz, C-3′ and C-5′), 127.2 (d, ^3^*J*_CF_ = 8.3 Hz, C-2′ and C-6′), 129.9 (d, ^4^*J*_CF_ = 3.5 Hz, C-1′), 142.4 (C-7), 150.4 & 150.8 (C-4 and C-8a), 161.9 (d, ^1^*J*_CF_ = 244.5 Hz, C-4′), 167.7 (C-2). Anal. Calcd for C_14_H_14_FN_5_: C, 61.98; H, 5.20; N, 25.81. Found: C, 61.92; H, 5.29; N, 25.72.

##### 4-Amino-2-propyl-7-(4-chlorophenyl)imidazo[1,2-*a*][1,3,5]triazine (5o)

Light brown solid, yield 16%, mp 294–295 °C (EtOH). ^1^H NMR (300 MHz, DMSO-*d*_6_): *δ* 0.94 (3H, t, ^3^*J* = 7.4 Hz, CH_3_), 1.76 (2H, m, ^3^*J* = 7.4 Hz, CH_2_), 2.60 (2H, t, ^3^*J* = 7.4 Hz, CH_2_), 7.53 (2H, d, ^3^*J* = 8.7 Hz, H-3′ and H-5′), 7.85 (2H, d, ^3^*J* = 8.9 Hz, H-2′ and H-6′), 8.17 (1H, s, H-6), 8.36 (2H, br s, NH_2_). ^13^C NMR (75 MHz, DMSO-*d*_6_): *δ* 13.6 (CH_3_), 20.4 (CH_2_), 40.2 (CH_2_), 102.1 (C-6), 126.9 (C-2′ and C-6′), 128.8 (C-3′ and C-5′), 132.2 (C-1′), 132.3 (C-4′), 142.1 (C-7), 150.4 & 150.8 (C-4 and C-8a), 167.8 (C-2). Anal. Calcd for C_14_H_14_ClN_5_: C, 58.44; H, 4.90; N, 24.34. Found: C, 58.37; H, 5.03; N, 24.22.

##### 4-Amino-2-propyl-7-(4-methyphenyl)imidazo[1,2-*a*][1,3,5]triazine (5p)

Light yellow solid, yield 50%, mp 305–307 °C (EtOH). ^1^H NMR (300 MHz, DMSO-*d*_6_): *δ* 0.94 (3H, t, ^3^*J* = 7.4 Hz, CH_3_), 1.77 (2H, m, ^3^*J* = 7.4 Hz, CH_2_), 2.34 (3H, s, CH_3_), 2.60 (2H, t, ^3^*J* = 7.4 Hz, CH_2_), 7.27 (2H, d, ^3^*J* = 7.3 Hz, H-3′ and H-5′), 7.75 (2H, d, ^3^*J* = 8.0 Hz, H-2′ and H-6′), 8.10 (1H, s, H-6), 8.30 (2H, br s, NH_2_). ^13^C NMR (75 MHz, DMSO-*d*_6_): *δ* 13.6 (CH_3_), 20.4 (CH_2_), 20.8 (CH_3_), 40.2 (CH_2_), 101.2 (C-6), 125.2 (C-3′ and C-5′), 129.3 (C-2′ and C-6′), 130.6 (C-1′), 137.3 (C-4′), 143.5 (C-7), 150.3 & 150.7 (C-4 and C-8a), 167.5 (C-2). Anal. Calcd for C_15_H_17_N_5_: C, 67.39; H, 6.41; N, 26.20. Found: C, 67.32; H, 6.53; N, 26.06.

##### 4-Amino-2-propyl-7-(4-methoxyphenyl)imidazo[1,2-*a*][1,3,5]triazine (5q)

White solid, yield 52%, mp 295–297 °C (MeOH). ^1^H NMR (300 MHz, DMSO-*d*_6_): *δ* 0.94 (3H, t, ^3^*J* = 7.4 Hz, CH_3_), 1.76 (2H, m, ^3^*J* = 7.4 Hz, CH_2_), 2.59 (2H, t, ^3^*J* = 7.4 Hz, CH_2_), 3.80 (3H, s, OCH_3_), 7.04 (2H, d, ^3^*J* = 8.9 Hz, H-3′ and H-5′), 7.78 (2H, d, ^3^*J* = 8.9 Hz, H-2′ and H-6′), 8.03 (1H, s, H-6), 8.30 (2H, br s, NH_2_). ^13^C NMR (75 MHz, DMSO-*d*_6_): *δ* 13.6 (CH_3_), 20.4 (CH_2_), 40.2 (CH_2_), 55.1 (OCH_3_), 100.4 (C-6), 114.2 (C-3′ and C-5′), 125.9 (C-1′), 126.6 (C-2′ and C-6′), 143.4 (C-7), 150.3 & 150.6 (C-4 and C-8a), 159.2 (C-4′), 167.3 (C-2). Anal. Calcd for C_15_H_17_N_5_O: C, 63.59; H, 6.05; N, 24.72. Found: C, 63.52; H, 6.20; N, 24.59.

##### 4-Amino-2-propyl-7-(4-biphenyl)imidazo[1,2-*a*][1,3,5]triazine (5r)

Light brown solid, yield 40%, mp 337–338 °C (DMF). ^1^H NMR (300 MHz, DMSO-*d*_6_): *δ* 0.95 (3H, t, ^3^*J* = 7.4 Hz, CH_3_), 1.78 (2H, m, ^3^*J* = 7.4 Hz, CH_2_), 2.62 (2H, t, ^3^*J* = 7.4 Hz, CH_2_), 7.38 (1H, t, ^3^*J* = 7.3 Hz, H-4′′), 7.49 (2H, t, ^3^*J* = 7.5 Hz, H-3′′ and H-5′′), 7.73 (2H, d, ^3^*J* = 7.2 Hz, H-2′′ and H-6′′), 7.79 (2H, d, ^3^*J* = 8.4 Hz, H-3′ and H-5′), 7.95 (2H, d, ^3^*J* = 8.3 Hz, H-2′ and H-6′), 8.21 (1H, s, H-6), 8.36 (2H, br s, NH_2_). ^13^C NMR (75 MHz, DMSO-*d*_6_): *δ* 13.7 (CH_3_), 20.4 (CH_2_), 40.2 (CH_2_), 101.9 (C-6), 125.8 (C-3′ and C-5′), 126.4 (C-3′′ and C-5′′), 127.0 (C-2′ and C-6′), 127.4 (C-4′′), 128.9 (C-2′′ and C-6′′), 132.4 (C-1′), 139.5 & 139.6 (C-4′ and C-1′′), 143.0 (C-7), 150.4 & 150.8 (C-4 and C-8a), 167.7 (C-2). Anal. Calcd for C_20_H_19_N_5_: C, 72.92; H, 5.81; N, 21.26. Found: C, 72.88; H, 5.94; N, 21.13.

##### 4-Amino-2-butyl-7-phenylimidazo[1,2-*a*][1,3,5]triazine (5s)

Light yellow solid, yield 30%, mp 267–269 °C (EtOH). ^1^H NMR (300 MHz, DMSO-*d*_6_): *δ* 0.92 (3H, t, ^3^*J* = 7.3 Hz, CH_3_), 1.36 (2H, m, ^3^*J* = 7.4 Hz, CH_2_), 1.72 (2H, m, ^3^*J* = 7.5 Hz, CH_2_), 2.62 (2H, t, ^3^*J* = 7.5 Hz, CH_2_), 7.35 (1H, t, ^3^*J* = 7.3 Hz, H-4′), 7.47 (2H, t, ^3^*J* = 7.5 Hz, H-3′ and H-5′), 7.85 (2H, d, ^3^*J* = 7.0 Hz, H-2′ and H-6′), 8.15 (1H, s, H-6), 8.35 (2H, br s, NH_2_). ^13^C NMR (75 MHz, DMSO-*d*_6_): *δ* 13.7 (CH_3_), 21.7 (CH_2_), 29.3 (CH_2_), 37.9 (CH_2_), 101.7 (C-6), 125.2 (C-2′ and C-6′), 127.9 (C-4′), 128.7 (C-3′ and C-5′), 133.3 (C-1′), 143.3 (C-7), 150.3 & 150.8 (C-4 and C-8a), 167.8 (C-2). Anal. Calcd for C_15_H_17_N_5_: C, 67.39; H, 6.41; N, 26.20. Found: C, 67.32; H, 6.55; N, 26.04.

##### 4-Amino-2-butyl-7-(4-fluorophenyl)imidazo[1,2-*a*][1,3,5]triazine (5t)

White solid, yield 29%, mp 296–298 °C (EtOH). ^1^H NMR (300 MHz, DMSO-*d*_6_): *δ* 0.92 (3H, t, ^3^*J* = 7.3 Hz, CH_3_), 1.36 (2H, m, ^3^*J* = 7.4 Hz, CH_2_), 1.72 (2H, m, ^3^*J* = 7.5 Hz, CH_2_), 2.62 (2H, t, ^3^*J* = 7.5 Hz, CH_2_), 7.31 (2H, dd, ^3^*J*_HF_ = 8.9 Hz, ^3^*J*_HH_ = 8.9 Hz, H-3′ and H-5′), 7.88 (2H, dd, ^4^*J*_HF_ = 5.6 Hz, ^3^*J*_HH_ = 8.9 Hz, H-2′ and H-6′), 8.12 (1H, s, H-6), 8.34 (2H, br s, NH_2_). ^13^C NMR (75 MHz, DMSO-*d*_6_): *δ* 13.8 (CH_3_), 21.7 (CH_2_), 29.3 (CH_2_), 37.9 (CH_2_), 101.5 (C-6), 115.7 (d, ^2^*J*_CF_ = 21.7 Hz, C-3′ and C-5′), 127.2 (d, ^3^*J*_CF_ = 8.3 Hz, C-2′ and C-6′), 129.9 (d, ^4^*J*_CF_ = 3.0 Hz, C-1′), 142.4 (C-7), 150.4 & 150.8 (C-4 and C-8a), 161.9 (d, ^1^*J*_CF_ = 244.8 Hz, C-4′), 167.9 (C-2). Anal. Calcd for C_15_H_16_FN_5_: C, 63.14; H, 5.65; N, 24.55. Found: C, 63.05; H, 5.77; N, 24.42.

##### 4-Amino-2-butyl-7-(4-chlorophenyl)imidazo[1,2-*a*][1,3,5]triazine (5u)

Light brown solid, yield 11%, mp 287–288 °C (EtOH). ^1^H NMR (300 MHz, DMSO-*d*_6_): *δ* 0.91 (3H, t, ^3^*J* = 7.3 Hz, CH_3_), 1.35 (2H, m, ^3^*J* = 7.4 Hz, CH_2_), 1.72 (2H, m, ^3^*J* = 7.5 Hz, CH_2_), 2.62 (2H, t, ^3^*J* = 7.5 Hz, CH_2_), 7.53 (2H, d, ^3^*J* = 8.6 Hz, H-3′ and H-5′), 7.84 (2H, d, ^3^*J* = 8.6 Hz, H-2′ and H-6′), 8.16 (1H, s, H-6), 8.36 (2H, br s, NH_2_). ^13^C NMR (75 MHz, DMSO-*d*_6_): *δ* 13.7 (CH_3_), 21.7 (CH_2_), 29.3 (CH_2_), 37.9 (CH_2_), 102.1 (C-6), 126.9 (C-2′ and C-6′), 128.8 (C-3′ and C-5′), 132.2 & 132.33 (C-1′ and C-4′), 142.1 (C-7), 150.4 & 150.8 (C-4 and C-8a), 168.1 (C-2). Anal. Calcd for C_15_H_16_ClN_5_: C, 59.70; H, 5.34; N, 23.21. Found: C, 59.63; H, 5.41; N, 23.15.

##### 4-Amino-2-butyl-7-(4-methylphenyl)imidazo[1,2-*a*][1,3,5]triazine (5v)

Light yellow solid, yield 25%, mp 287–289 °C (EtOH). ^1^H NMR (300 MHz, DMSO-*d*_6_): *δ* 0.92 (3H, t, ^3^*J* = 7.3 Hz, CH_3_), 1.36 (2H, m, ^3^*J* = 7.4 Hz, CH_2_), 1.72 (2H, m, ^3^*J* = 7.5 Hz, CH_2_), 2.34 (3H, s, CH_3_), 2.62 (2H, t, ^3^*J* = 7.5 Hz, CH_2_), 7.27 (2H, d, ^3^*J* = 7.9 Hz, H-3′ and H-5′), 7.74 (2H, d, ^3^*J* = 8.1 Hz, H-2′ and H-6′), 8.09 (1H, s, H-6), 8.30 (2H, br s, NH_2_). ^13^C NMR (75 MHz, DMSO-*d*_6_): *δ* 13.7 (CH_3_), 20.8 (CH_3_), 21.7 (CH_2_), 29.3 (CH_2_), 37.9 (CH_2_), 101.2 (C-6), 125.2 (C-2′ and C-6′), 129.3 (C-3′ and C-5′), 130.5 (C-1′), 137.3 (C-4′), 143.5 (C-7), 150.3 & 150.7 (C-4 and C-8a), 167.7 (C-2). Anal. Calcd for C_16_H_19_N_5_: C, 68.30; H, 6.81; N, 24.89. Found: C, 68.24; H, 6.90; N, 24.81.

##### 4-Amino-2-butyl-7-(4-methoxyphenyl)imidazo[1,2-*a*][1,3,5]triazine (5w)

Light yellow solid, yield 25%, mp 279–281 °C (MeOH). ^1^H NMR (300 MHz, DMSO-*d*_6_): *δ* 0.92 (3H, t, ^3^*J* = 7.3 Hz, CH_3_), 1.36 (2H, m, ^3^*J* = 7.4 Hz, CH_2_), 1.72 (2H, m, ^3^*J* = 7.5 Hz, CH_2_), 2.62 (2H, t, ^3^*J* = 7.5 Hz, CH_2_), 3.81 (3H, s, OCH_3_), 7.04 (2H, d, ^3^*J* = 8.9 Hz, H-3′ and H-5′), 7.78 (2H, d, ^3^*J* = 8.8 Hz, H-2′ and H-6′), 8.03 (1H, s, H-6), 8.30 (2H, br s, NH_2_). ^13^C NMR (75 MHz, DMSO-*d*_6_): *δ* 13.8 (CH_3_), 21.7 (CH_2_), 29.3 (CH_2_), 37.9 (CH_2_), 55.1 (OCH_3_), 100.4 (C-6), 114.2 (C-3′ and C-5′), 125.9 (C-1′), 126.6 (C-2′ and C-6′), 143.4 (C-7), 150.3 & 150.6 (C-4 and C-8a), 159.2 (C-4′), 167.6 (C-2). Anal. Calcd for C_16_H_19_N_5_O: C, 64.63; H, 6.44; N, 23.55. Found: C, 64.55; H, 6.54; N, 23.43.

##### 4-Amino-2-butyl-7-(4-biphenyl)imidazo[1,2-*a*][1,3,5]triazine (5x)

Light brown solid, yield 43%, mp 327–329 °C (DMF). ^1^H NMR (300 MHz, DMSO-*d*_6_): *δ* 0.92 (3H, t, ^3^*J* = 7.3 Hz, CH_3_), 1.37 (2H, m, ^3^*J* = 7.4 Hz, CH_2_), 1.73 (2H, m, ^3^*J* = 7.5 Hz, CH_2_), 2.63 (2H, t, ^3^*J* = 7.5 Hz, CH_2_), 7.38 (1H, t, ^3^*J* = 7.3 Hz, H-4′′), 7.49 (2H, t, ^3^*J* = 7.5 Hz, H-3′′ and H-5′′), 7.73 (2H, d, ^3^*J* = 7.5 Hz, H-2′′ and H-6′′), 7.79 (2H, d, ^3^*J* = 8.4 Hz, H-3′ and H-5′), 7.94 (2H, d, ^3^*J* = 8.4 Hz, H-2′ and H-6′), 8.20 (1H, s, H-6), 8.36 (2H, br s, NH_2_). ^13^C NMR (75 MHz, DMSO-*d*_6_): *δ* 13.7 (CH_3_), 21.7 (CH_2_), 29.3 (CH_2_), 37.9 (CH_2_), 101.8 (C-6), 125.8 (C-3′ and C-5′), 126.4 (C-3′′ and C-5′′), 127.0 (C-2′ and C-6′), 127.4 (C-4′′), 128.9 (C-2′′ and C-6′′), 132.4 (C-1′), 139.5 (C-4′ and C-1′′), 143.0 (C-7), 150.4 & 150.7 (C-4 and C-8a), 167.9 (C-2). Anal. Calcd for C_21_H_21_N_5_: C, 73.44; H, 6.16; N, 20.39. Found: C, 73.38; H, 6.26; N, 20.31.

#### Synthesis of 4-amino-2-methyl-7-phenylimidazo[1,2-*a*][1,3,5]triazine (5a) using Monowave 400 (Anton Paar) microwave synthesizer

The mixture of 2-amino-4-phenyl-1*H*-imidazole (**1a**, 159 mg, 1 mmol), cyanamide (105 mg, 2.5 mmol) and triethyl orthoacetate (0.46 mL, 2.5 mmol) in ethyl acetate (2 mL) were irradiated in a 10 mL seamless pressure vial using a microwave system operating at maximal microwave power up to 850 W (Monowave 400, Anton Paar) at 160 °C for 35 min. After cooling, the precipitate was filtered and washed with ethyl acetate to give 5a in the 56% yield.

#### Synthesis of 4-amino-2-methyl-7-phenylimidazo[1,2-*a*][1,3,5]triazine (5a) *via* heating in a sealed vessel

The mixture of 2-amino-4-phenyl-1*H*-imidazole (**1a**, 159 mg, 1 mmol), cyanamide (105 mg, 2.5 mmol) and triethyl orthoacetate (0.46 mL, 2.5 mmol) in ethyl acetate (2 mL) were heated in a 10 mL seamless pressure vial in an enclosed system (Monowave 50, Anton Paar) at 160 °C for 35 min. After cooling, the precipitate was filtered to obtain a compound, which was identical to 5a prepared under microwave irradiation. Yield 54%.

#### Step-wise approach for the synthesis of 4-amino-2-methyl-7-phenylimidazo[1,2-*a*][1,3,5]triazine (5a)

##### *N*′,*N*′-Morpholino-*N*-[3(5)-phenylimidazolo-5(3)yl acetamidine (6)

A mixture of 2-amino-1*H*-imidazole 1a (159 mg, 1 mmol) with triethyl orthoacetate (0.46 mL, 2.5 mmol) and morpholine (0.22 mL, 2.5 mmol) in ethyl acetate (2 mL) were irradiated in a 10 mL seamless pressure vial using the Discover SP (CEM) microwave system operating at maximal microwave power up to 150 W at 160 °C for 35 min. After cooling, the precipitate was filtered and recrystallised from EtOH to give pure formamidine 6. Light yellow solid, yield 34%, mp 217–219 °C (EtOH).^1^H NMR (300 MHz, DMSO-*d*_6_): *δ* 2.28 (3H, s, CH_3_), 3.55–3.56 (4H, m, (CH_2_)_2_), 3.62–3.65 (4H, m, (CH_2_)_2_), 7.10 (1H, t, ^3^*J* = 7.3 Hz, H-4′), 7.22 (1H, s, CH), 7.29 (2H, t, ^3^*J* = 7.7 Hz, H-3′ and H-5′), 7.66 (2H, d, ^3^*J* = 6.9 Hz, H-2′ and H-6′), 11.08 & 11.44 (1H, br s, NH). Anal. Calcd for C_15_H_18_N_4_O: C, 66.64; H, 6.71; N, 20.73. Found: C, 66.46; H, 6.94; N, 20.55.

##### 4-Amino-2-methyl-7-phenylimidazo[1,2-*a*][1,3,5]triazine (5a)

A mixture of 6 (270 mg, 1 mmol) with cyanamide (105 mg, 2.5 mmol) in ethyl acetate (2 mL) were irradiated in a 10 mL seamless pressure vial using the Discover SP (CEM) microwave system operating at maximal microwave power up to 150 W at 160 °C for 35 min. After cooling, the precipitate was filtered to obtain a compound, which was identical to 5a prepared using the multicomponent reaction. Yield 43%.

#### X-Ray crystallographic analysis

Intensity data for 5g and 5p were measured at 100 K on an Rigaku/Oxford Diffraction XtaLAB Synergy diffractometer (Dualflex, AtlasS2) fitted with CuKα radiation (*λ* = 1.54178 Å) so that *θ*_max_ = 67.1°. Data reduction and Gaussian absorption corrections were by standard methods.^22^ The structures were solved by direct-methods^23^ and refined on *F*^2^ (ref. 24) with anisotropic displacement parameters, C-bound H atoms in the riding model approximation and N-bound H atoms refined with N–H = 0.88 ± 0.01 Å. A weighting scheme of the form *w* = 1/[*σ*^2^(*F*_o_^2^) + (*aP*)^2^ + *bP*] where *P* = (*F*_o_^2^ + 2*F*_c_^2^)/3 was introduced in each case. The molecular structure diagrams showing 70% probability displacement ellipsoids were generated by ORTEP for Windows^25^ and the packing diagrams with DIAMOND.^26^ Additional data analysis was made with PLATON.^27^

##### Crystal data for 4-amino-2-ethyl-7-phenylimidazo[1,2-*a*][1,3,5]triazine (5g)

*M* = 239.28, orthorhombic, *Pccn*, *a* = 16.6173(2), *b* = 11.17070(10), *c* = 12.4074(2) Å, *V* = 2303.15(5) Å^3^, *Z* = 8, *D*_x_ = 1.380 g cm^−3^, *F*(000) = 1008, *μ* = 0.709 mm^−1^, no. reflns meas. = 14 762, no. unique reflns = 2060 (*R*_int_ = 0.026), no. reflns with *I* ≥ 2*σ*(*I*) = 1935, no. parameters = 170, *R*(obs. data) = 0.032, *wR*2(all data) = 0.085. CCDC deposition number: 1840247.

##### Crystal data for 4-amino-2-propyl-7-(4-methyphenyl)imidazo[1,2-*a*][1,3,5]triazine (5p)

*M* = 267.33, triclinic, *P*1̄, *a* = 10.7534(2), *b* = 11.8995(2), *c* = 12.1124(3) Å, *α* = 71.918(2), *β* = 68.962(2), *γ* = 78.712(2)°, *V* = 1368.93(5) Å^3^, *Z* = 4, *D*_x_ = 1.297 g cm^−3^, *F*(000) = 568, *μ* = 0.650 mm^−1^, no. reflns meas. = 32 816, no. unique reflns = 4893 (*R*_int_ = 0.029), no. reflns with *I* ≥ 2*σ*(*I*) = 4530, no. parameters = 377, *R*(obs. data) = 0.034, *wR*2(all data) = 0.092. CCDC deposition number: 1840248.

## Conflicts of interest

There are no conflicts to declare.

## Supplementary Material

RA-008-C8RA03703E-s001

RA-008-C8RA03703E-s002
