# Supplementary material for: A one-pot, multicomponent reaction for the synthesis of novel 2-alkyl substituted 4-aminoimidazo[1,2-a][1,3,5]triazines
Source: RSC Adv. 2018 Jun 12;8(38):21495–504. doi: 10.1039/c8ra03703e (PMC9080918; doi:10.1039/c8ra03703e)
Supplement: RA-008-C8RA03703E-s001 [file RA-008-C8RA03703E-s001.pdf]

Electronic Supplementary Material (ESI) for RSC Advances.
This journal is © The Royal Society of Chemistry 2018

RSC Advances

A one-pot, multicomponent reaction for the synthesis of novel 2-alkyl substituted 4-aminoimidazo[1,2-*a*][1,3,5]triazines

(Electronic Supplementary Information)

Felicia Phei Lin Lim,[a] Lin Yuing Tan,[a] Edward R. T. Tiekink,[b] Anton V. Dolzhenko[a,c]*

[a] *School of Pharmacy, Monash University Malaysia, Jalan Lagoon Selatan, Bandar Sunway, Selangor Darul Ehsan 47500, Malaysia. E-mail:dolzhenkoav@gmail.com; anton.dolzhenko@monash.edu; Fax: +60-3-5514-6364; Tel: +60-3-5514-5867*

[b] *Research Centre for Crystalline Materials, School of Science and Technology, Sunway University, Bandar Sunway, Selangor Darul Ehsan 47500, Malaysia.*

[c] *School of Pharmacy, Curtin Health Innovation Research Institute, Faculty of Health Sciences, Curtin University, GPO Box U1987 Perth, Western Australia 6845, Australia.*

Table of Contents

^{1}H and ^{13}C NMR spectra for compounds **5a**-**x** and **6**	S3
X-Ray crystallographic data for **5g** and **5p**	S57

^{1}H and ^{13}C NMR spectra for compounds **5a**-**x** and **6**

4-Amino-2-methyl-7-phenylimidazo[1,2-a][1,3,5]triazine (5a)

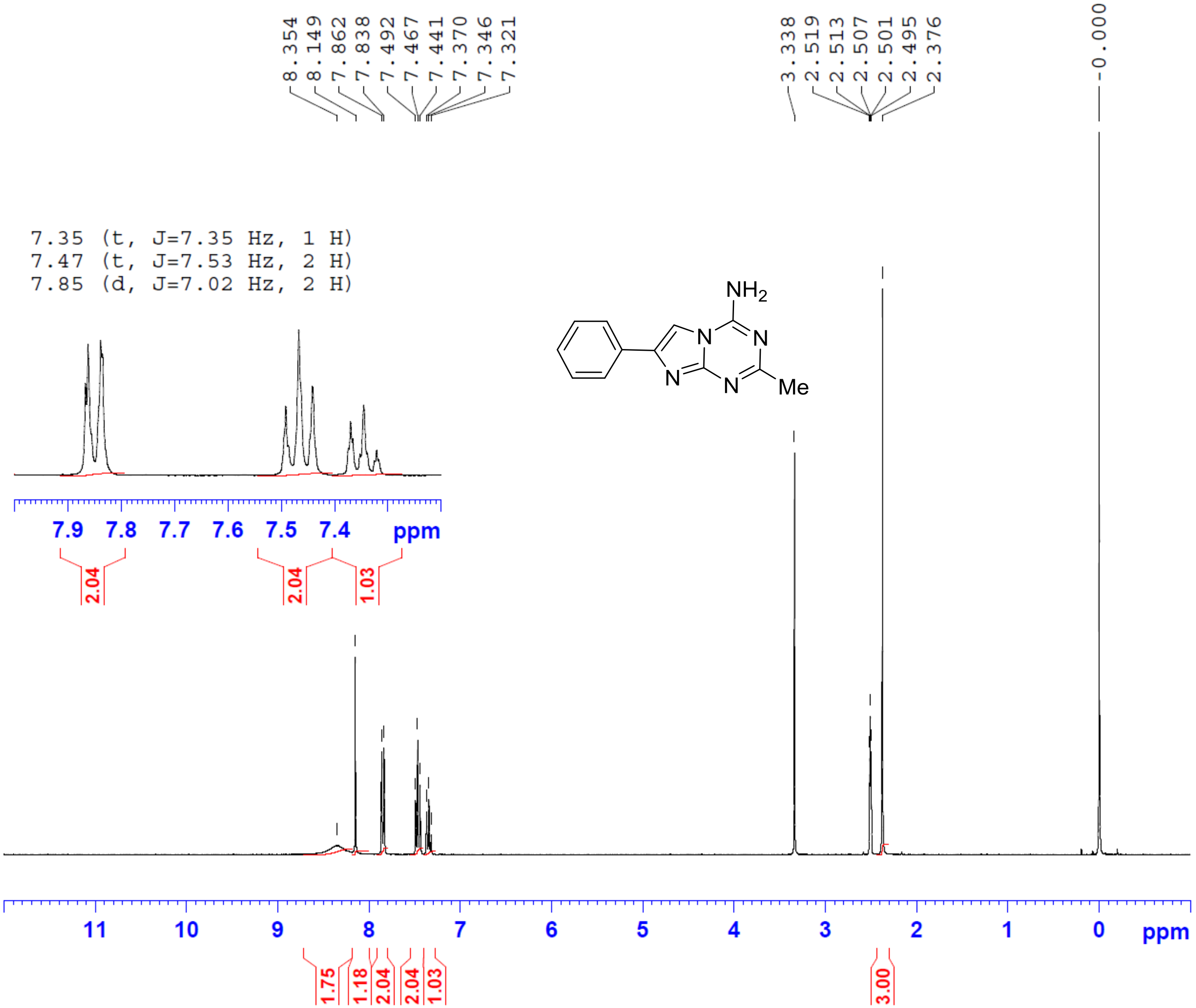

Current Data Parameters
NAME FL259
EXPNO 100
PROCNO 1

F2 - Acquisition Parameters
Date_ 20170513
Time 16.53
INSTRUM FOURIER300
PROBHD 5 mm DUL 13C-1
PULPROG zg30
TD 65536
SOLVENT DMSO
NS 16
DS 2
SWH 6103.516 Hz
FIDRES 0.093132 Hz
AQ 5.3687091 sec
RG 113.158
DW 81.920 usec
DE 6.50 usec
TE 297.5 K
D1 1.00000000 sec
TD0 1

======== CHANNEL f1 ========
SFO1 300.1618536 MHz
NUC1 1H
P1 13.50 usec
PLW1 9.30000019 W

F2 - Processing parameters
SI 65536
SF 300.1599988 MHz
WDW EM
SSB 0
LB 0.30 Hz
GB 0
PC 1.00

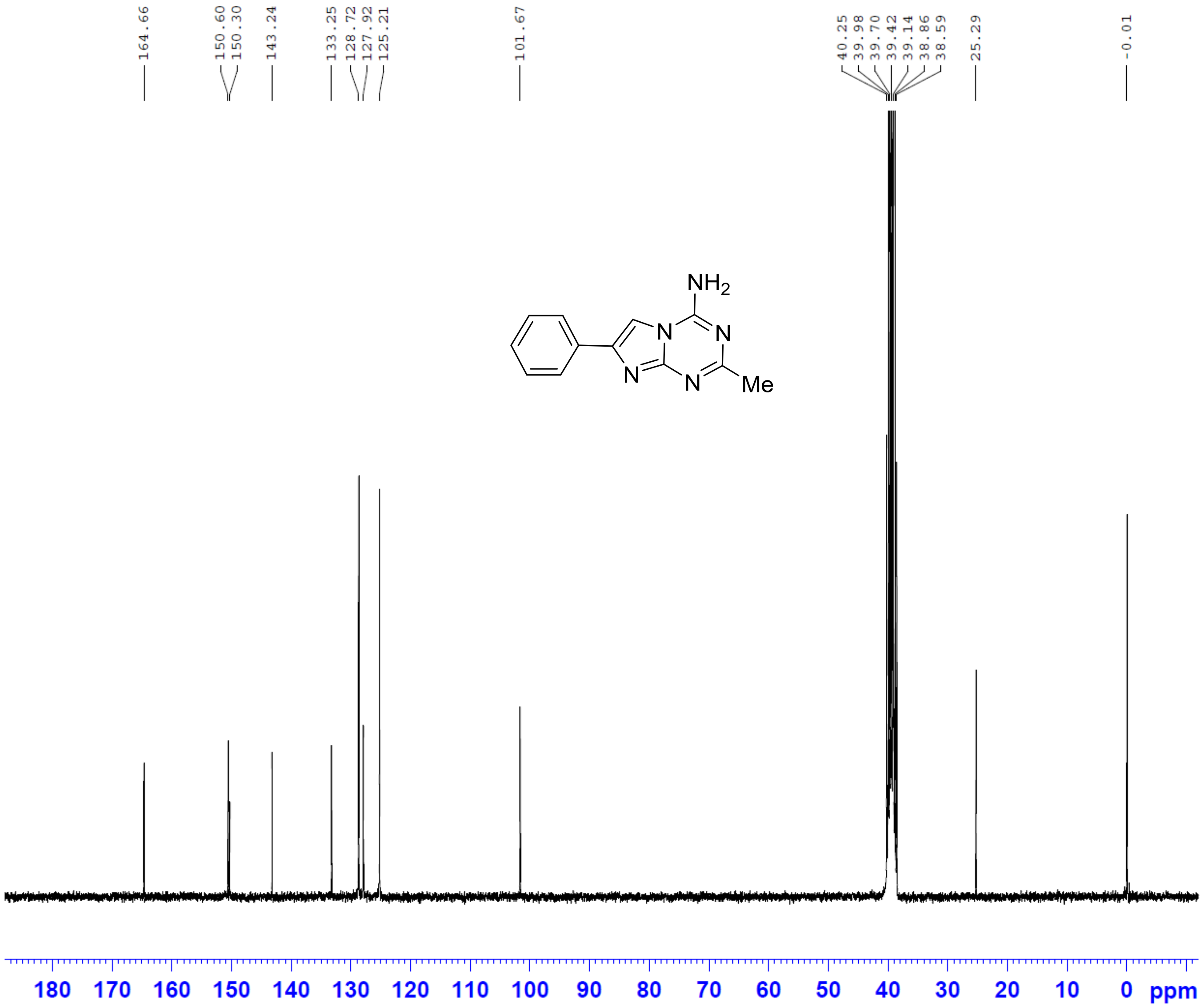


```
Current Data Parameters
NAME                FL259
EXPNO                 101
PROCNO                  1

F2 - Acquisition Parameters
Date_            20170513
Time                17.00
INSTRUM       FOURIER300
PROBHD    5 mm DUL 13C-1
PULPROG           zgpg30
TD                 65536
SOLVENT             DMSO
NS                 12288
DS                     4
SWH            24414.063 Hz
FIDRES          0.372529 Hz
AQ             1.3421773 sec
RG               501.187
DW                20.480 usec
DE                  6.50 usec
TE                 297.7 K
D1            2.00000000 sec
D11           0.03000000 sec
D31           0.00001500 sec
D40           0.00439029 sec
L4                    37
L5                    53
P32                98.00 usec
TD0                   12

======== CHANNEL f1 ========
SFO1         75.4828392 MHz
NUC1                13C
P1                15.00 usec
PLW1        22.00000000 W

======== CHANNEL f2 ========
SFO2        300.1612006 MHz
NUC2                 1H
CPDPRG[2         waltz16
PCPD2             98.00 usec
PLW2         9.30000019 W
PLW12        0.29359001 W
PLW13        0.20359001 W

F2 - Processing parameters
SI                 32768
SF            75.4753350 MHz
WDW                   EM
SSB       0
LB                  1.00 Hz
GB        0
PC                  1.40
```

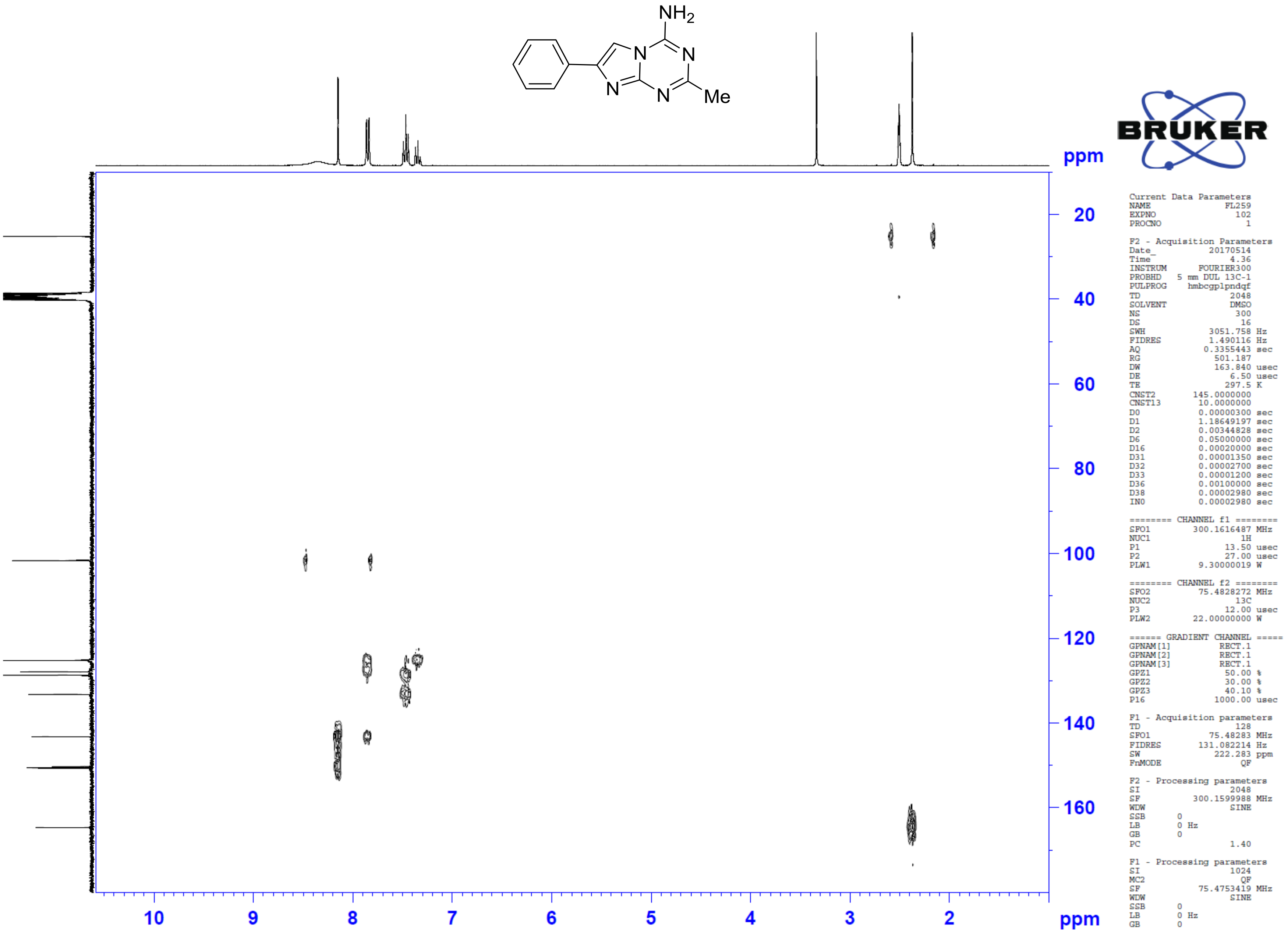
NH2
N
N
N
N
N
Me
ppm
20
40
60
80
100
120
140
160
10
9
8
7
6
5
4
3
2
ppm
BRUKER
Current Data Parameters
NAME FL259
EXPNO 102
PROCNO 1
F2 - Acquisition Parameters
Date_ 20170514
Time 4.36
INSTRUM FOURIER300
PROBHD 5 mm DUL 13C-1
PULPROG hmbcgplpndqf
TD 2048
SOLVENT DMSO
NS 300
DS 16
SWH 3051.758 Hz
FIDRES 1.490116 Hz
AQ 0.3355443 sec
RG 501.187
DW 163.840 usec
DE 6.50 usec
TE 297.5 K
CNST2 145.0000000
CNST13 10.0000000
D0 0.00000300 sec
D1 1.18649197 sec
D2 0.00344828 sec
D6 0.05000000 sec
D16 0.00020000 sec
D31 0.00001350 sec
D32 0.00002700 sec
D33 0.00001200 sec
D36 0.00100000 sec
D38 0.00002980 sec
IN0 0.00002980 sec
======== CHANNEL f1 ========
SFO1 300.1616487 MHz
NUC1 1H
P1 13.50 usec
P2 27.00 usec
PLW1 9.30000019 W
======== CHANNEL f2 ========
SFO2 75.4828272 MHz
NUC2 13C
P3 12.00 usec
PLW2 22.00000000 W
====== GRADIENT CHANNEL =====
GPNAM[1] RECT.1
GPNAM[2] RECT.1
GPNAM[3] RECT.1
GPZ1 50.00 %
GPZ2 30.00 %
GPZ3 40.10 %
P16 1000.00 usec
F1 - Acquisition parameters
TD 128
SFO1 75.48283 MHz
FIDRES 131.082214 Hz
SW 222.283 ppm
FnMODE QF
F2 - Processing parameters
SI 2048
SF 300.1599988 MHz
WDW SINE
SSB 0
LB 0 Hz
GB 0
PC 1.40
F1 - Processing parameters
SI 1024
MC2 QF
SF 75.4753419 MHz
WDW SINE
SSB 0
LB 0 Hz
GB 0

4-Amino-2-methyl-7-(4-fluorophenyl)imidazo[1,2-a][1,3,5]triazine (5b)

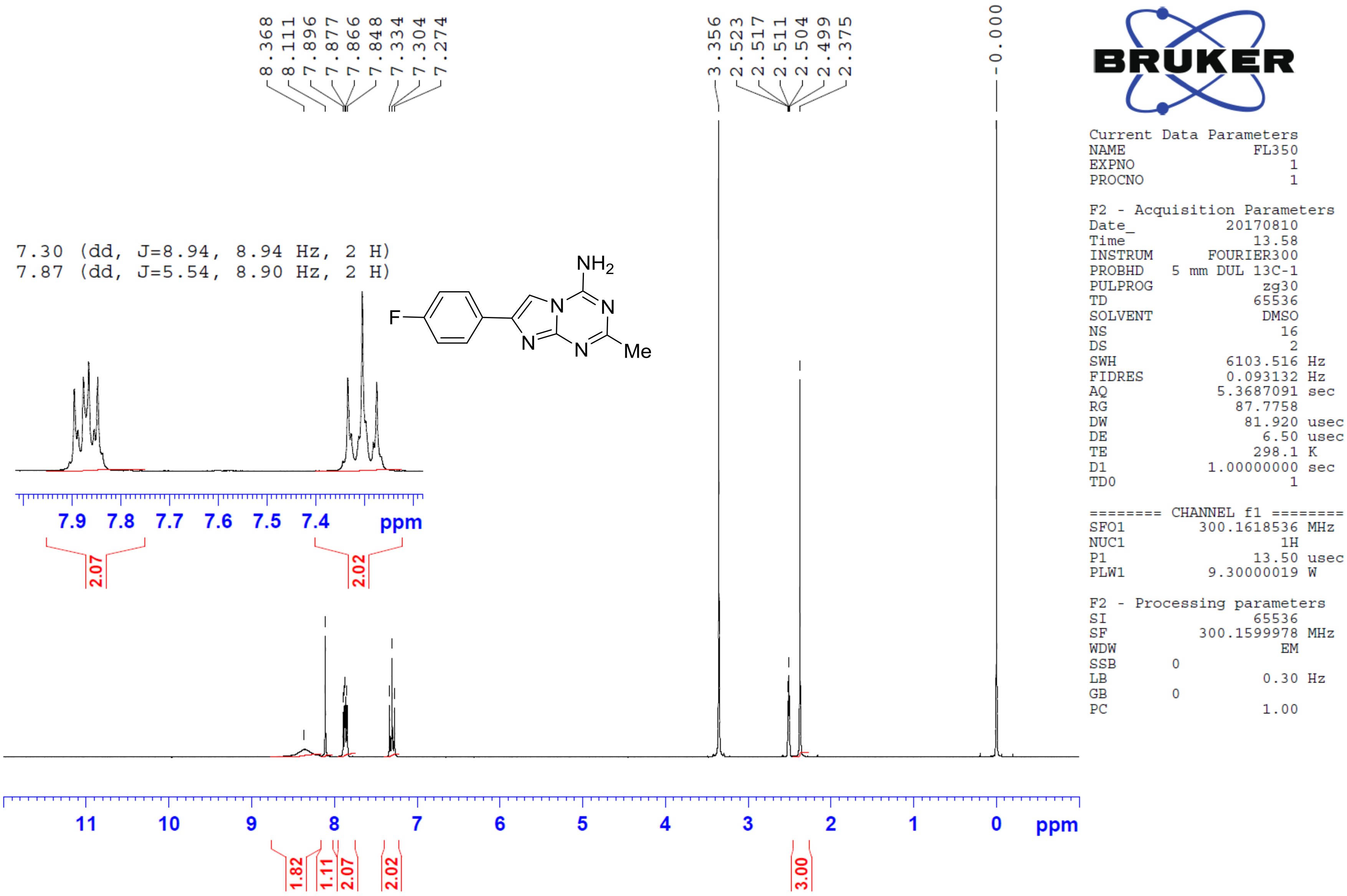

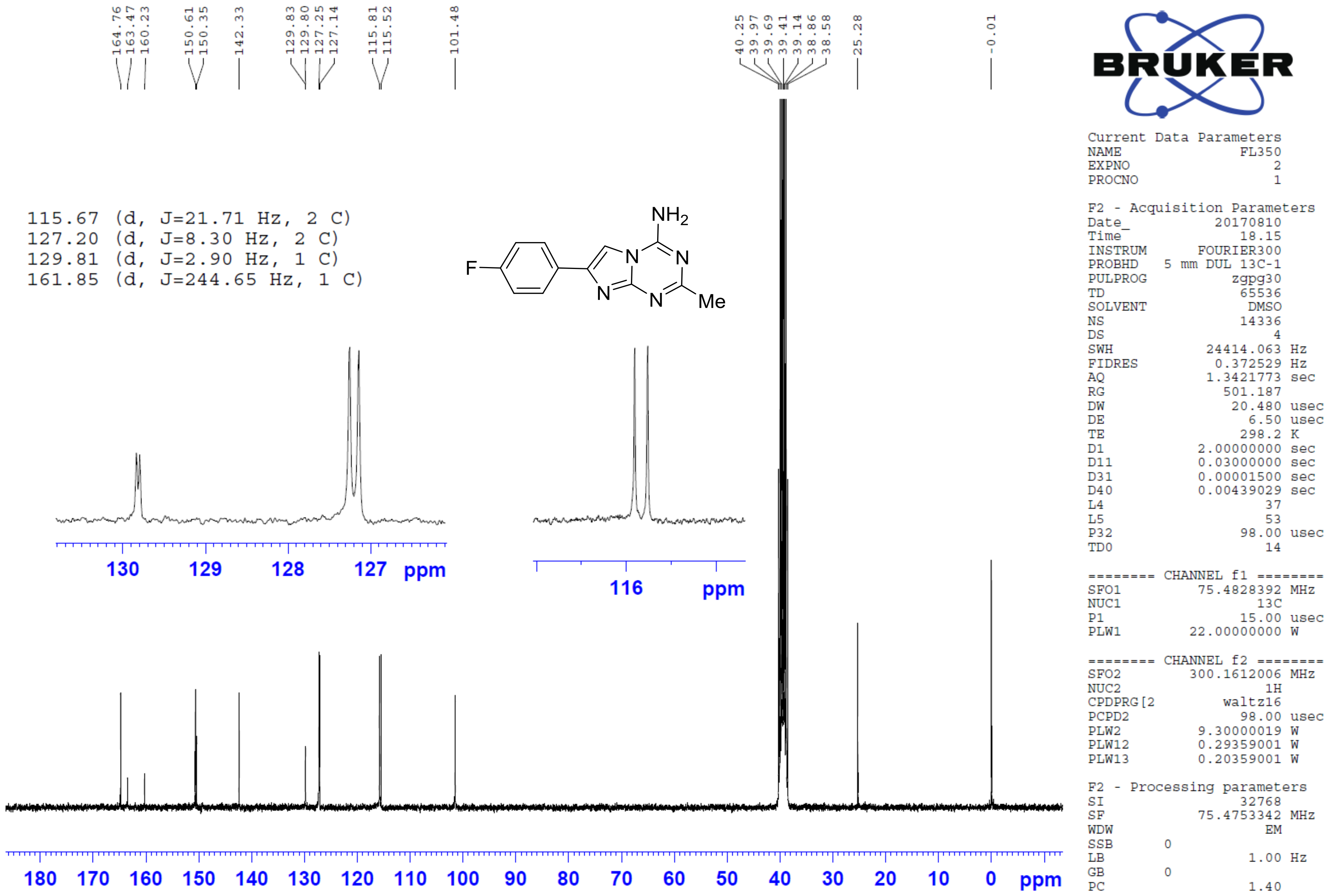
164.76
163.47
160.23
150.61
150.35
142.33
129.83
129.80
127.25
127.14
115.81
115.52
101.48
40.25
39.97
39.69
39.41
39.14
38.86
38.58
25.28
-0.01
115.67 (d, J=21.71 Hz, 2 C)
127.20 (d, J=8.30 Hz, 2 C)
129.81 (d, J=2.90 Hz, 1 C)
161.85 (d, J=244.65 Hz, 1 C)
F
NH2
N
N
N
N
Me
130
129
128
127
ppm
116
ppm
180
170
160
150
140
130
120
110
100
90
80
70
60
50
40
30
20
10
0
ppm
BRUKER
Current Data Parameters
NAME FL350
EXPNO 2
PROCNO 1
F2 - Acquisition Parameters
Date_ 20170810
Time 18.15
INSTRUM FOURIER300
PROBHD 5 mm DUL 13C-1
PULPROG zgpg30
TD 65536
SOLVENT DMSO
NS 14336
DS 4
SWH 24414.063 Hz
FIDRES 0.372529 Hz
AQ 1.3421773 sec
RG 501.187
DW 20.480 usec
DE 6.50 usec
TE 298.2 K
D1 2.00000000 sec
D11 0.03000000 sec
D31 0.00001500 sec
D40 0.00439029 sec
L4 37
L5 53
P32 98.00 usec
TD0 14
======== CHANNEL f1 ========
SFO1 75.4828392 MHz
NUC1 13C
P1 15.00 usec
PLW1 22.00000000 W
======== CHANNEL f2 ========
SFO2 300.1612006 MHz
NUC2 1H
CPDPRG[2 waltz16
PCPD2 98.00 usec
PLW2 9.30000019 W
PLW12 0.29359001 W
PLW13 0.20359001 W
F2 - Processing parameters
SI 32768
SF 75.4753342 MHz
WDW EM
SSB 0
LB 1.00 Hz
GB 0
PC 1.40

4-Amino-2-methyl-7-(4-chlorophenyl)imidazo[1,2-a][1,3,5]triazine (5c)

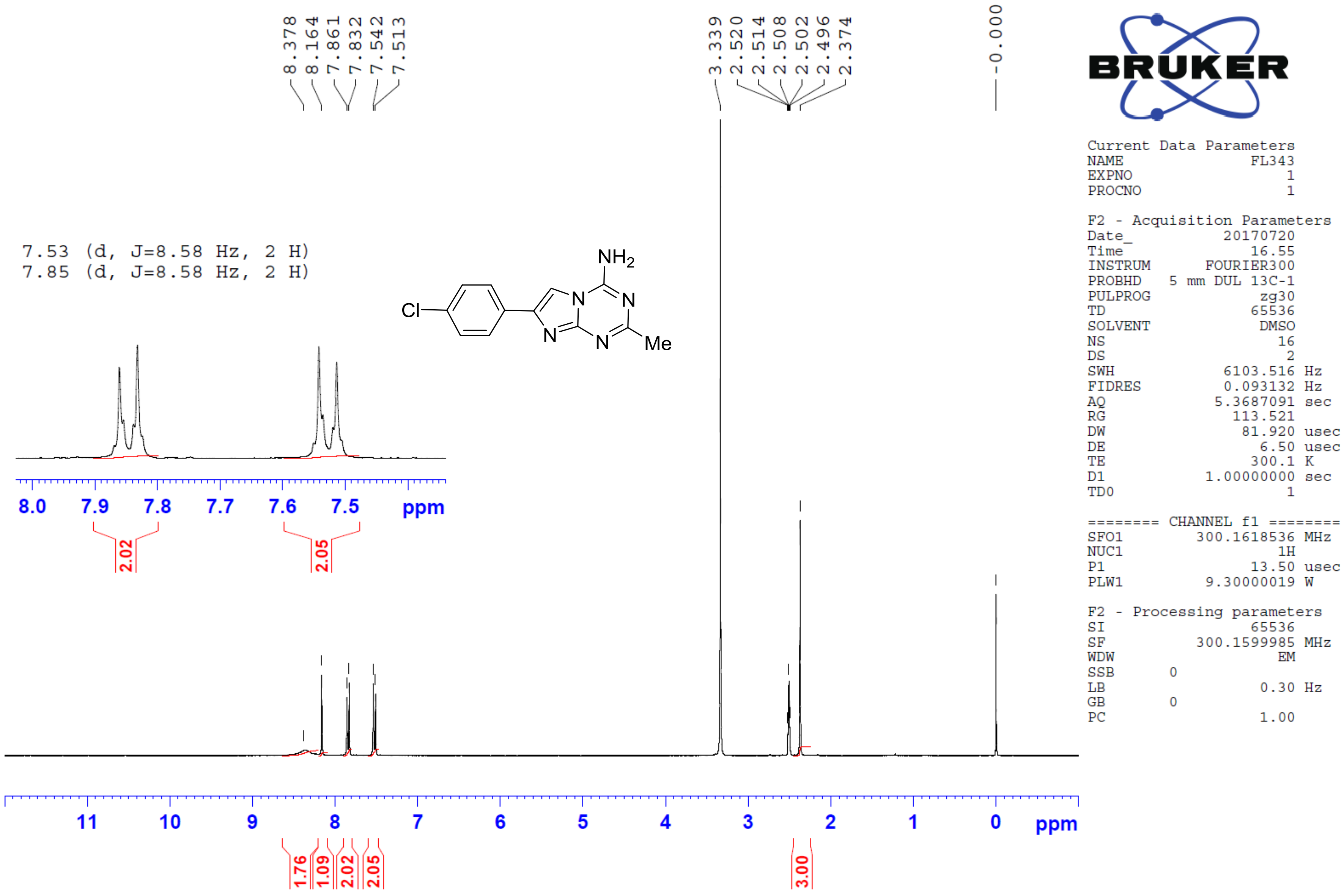

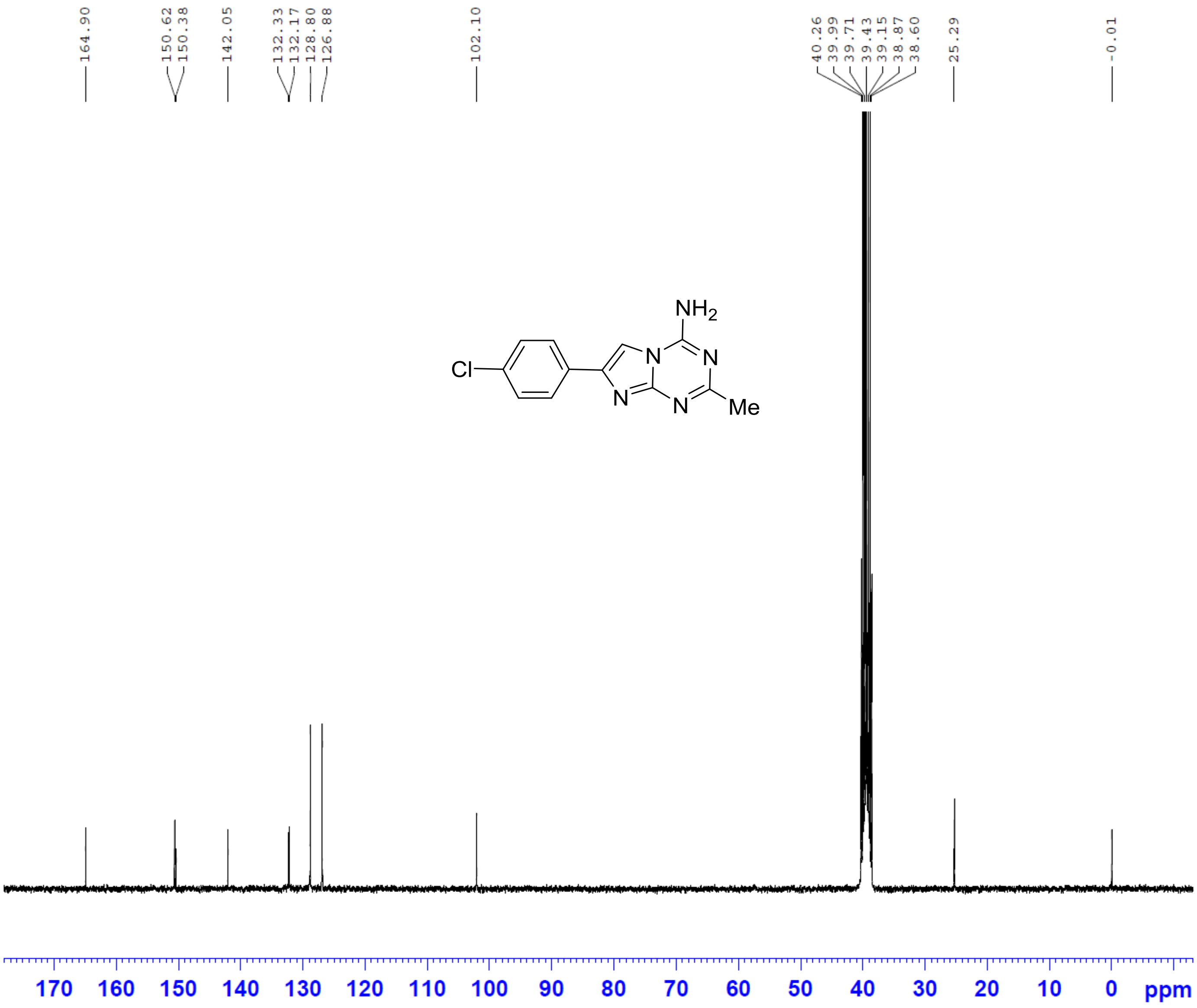

BRUKER

```
Current Data Parameters
NAME                FL343
EXPNO                   2
PROCNO                  1

F2 - Acquisition Parameters
Date_            20170720
Time                17.41
INSTRUM        FOURIER300
PROBHD    5 mm DUL 13C-1
PULPROG            zgpg30
TD                  65536
SOLVENT              DMSO
NS                  14336
DS                      4
SWH             24414.063 Hz
FIDRES           0.372529 Hz
AQ              1.3421773 sec
RG                501.187
DW                 20.480 usec
DE                   6.50 usec
TE                  300.2 K
D1             2.00000000 sec
D11            0.03000000 sec
D31            0.00001500 sec
D40            0.00439029 sec
L4                     37
L5                     53
P32                 98.00 usec
TD0                    14

======== CHANNEL f1 ========
SFO1           75.4828392 MHz
NUC1                  13C
P1                  15.00 usec
PLW1          22.00000000 W

======== CHANNEL f2 ========
SFO2          300.1612006 MHz
NUC2                   1H
CPDPRG[2          waltz16
PCPD2               98.00 usec
PLW2           9.30000019 W
PLW12          0.29359001 W
PLW13          0.20359001 W

F2 - Processing parameters
SI                  32768
SF             75.4753350 MHz
WDW                    EM
SSB       0
LB                   1.00 Hz
GB        0
PC                   1.40
```

4-Amino-2-methyl-7-(4-methylphenyl)imidazo[1,2-a][1,3,5]triazine (5d)

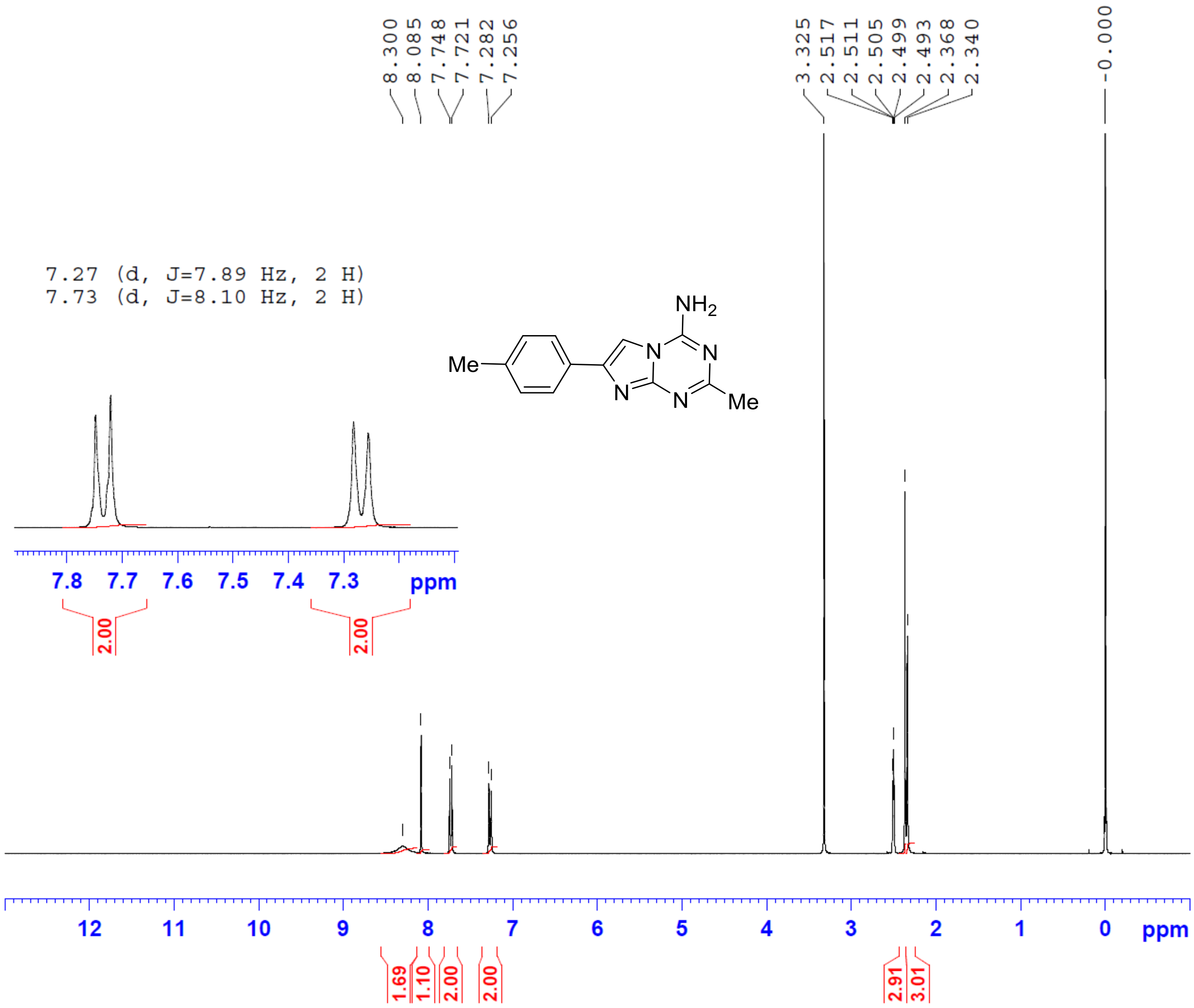


```
Current Data Parameters
NAME              FL359
EXPNO                 1
PROCNO                1

F2 - Acquisition Parameters
Date_          20170913
Time              14.53
INSTRUM      FOURIER300
PROBHD   5 mm DUL 13C-1
PULPROG            zg30
TD                65536
SOLVENT            DMSO
NS                   16
DS                    2
SWH            6103.516 Hz
FIDRES         0.093132 Hz
AQ            5.3687091 sec
RG              107.611
DW               81.920 usec
DE                 6.50 usec
TE                299.9 K
D1           1.00000000 sec
TD0                   1

======== CHANNEL f1 ========
SFO1        300.1618536 MHz
NUC1                 1H
P1                13.50 usec
PLW1         9.30000019 W

F2 - Processing parameters
SI                65536
SF          300.1599994 MHz
WDW                  EM
SSB       0
LB                 0.30 Hz
GB        0
PC                 1.00
```

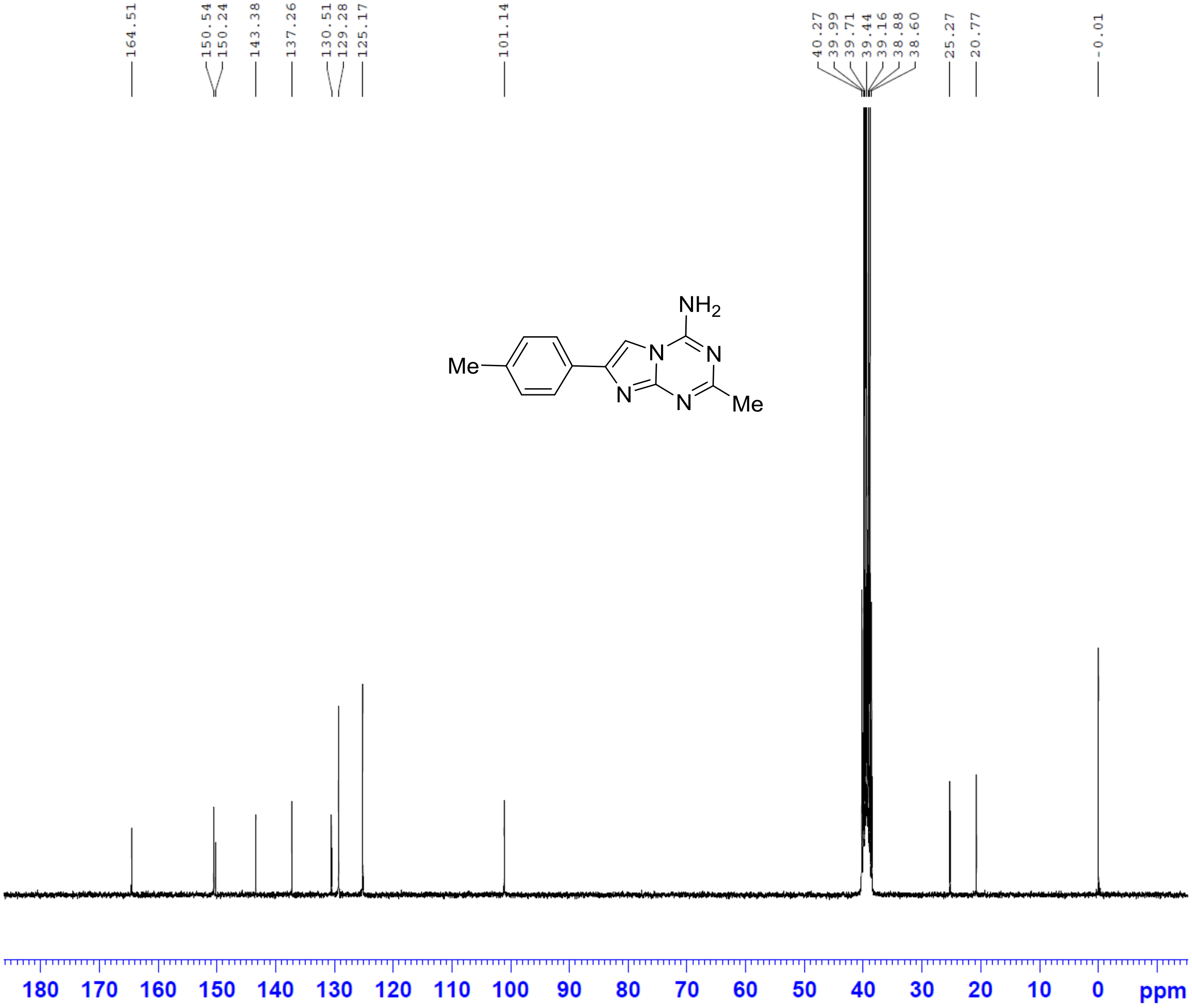

Current Data Parameters	
NAME	FL359
EXPNO	2
PROCNO	1
F2 - Acquisition Parameters	
Date_	20170914
Time	18.05
INSTRUM	FOURIER300
PROBHD	5 mm DUL 13C-1
PULPROG	zgpg30
TD	65536
SOLVENT	DMSO
NS	15360
DS	4
SWH	24414.063 Hz
FIDRES	0.372529 Hz
AQ	1.3421773 sec
RG	501.187
DW	20.480 usec
DE	6.50 usec
TE	300.1 K
D1	2.00000000 sec
D11	0.03000000 sec
D31	0.00001500 sec
D40	0.00439029 sec
L4	37
L5	53
P32	98.00 usec
TD0	15
======== CHANNEL f1 ========	
SFO1	75.4828392 MHz
NUC1	13C
P1	15.00 usec
PLW1	22.00000000 W
======== CHANNEL f2 ========	
SFO2	300.1612006 MHz
NUC2	1H
CPDPRG[2	waltz16
PCPD2	98.00 usec
PLW2	9.30000019 W
PLW12	0.29359001 W
PLW13	0.20359001 W
F2 - Processing parameters	
SI	32768
SF	75.4753350 MHz
WDW	EM
SSB	0
LB	1.00 Hz
GB	0
PC	1.40

4-Amino-2-methyl-7-(4-methoxyphenyl)imidazo[1,2-a][1,3,5]triazine (5e)

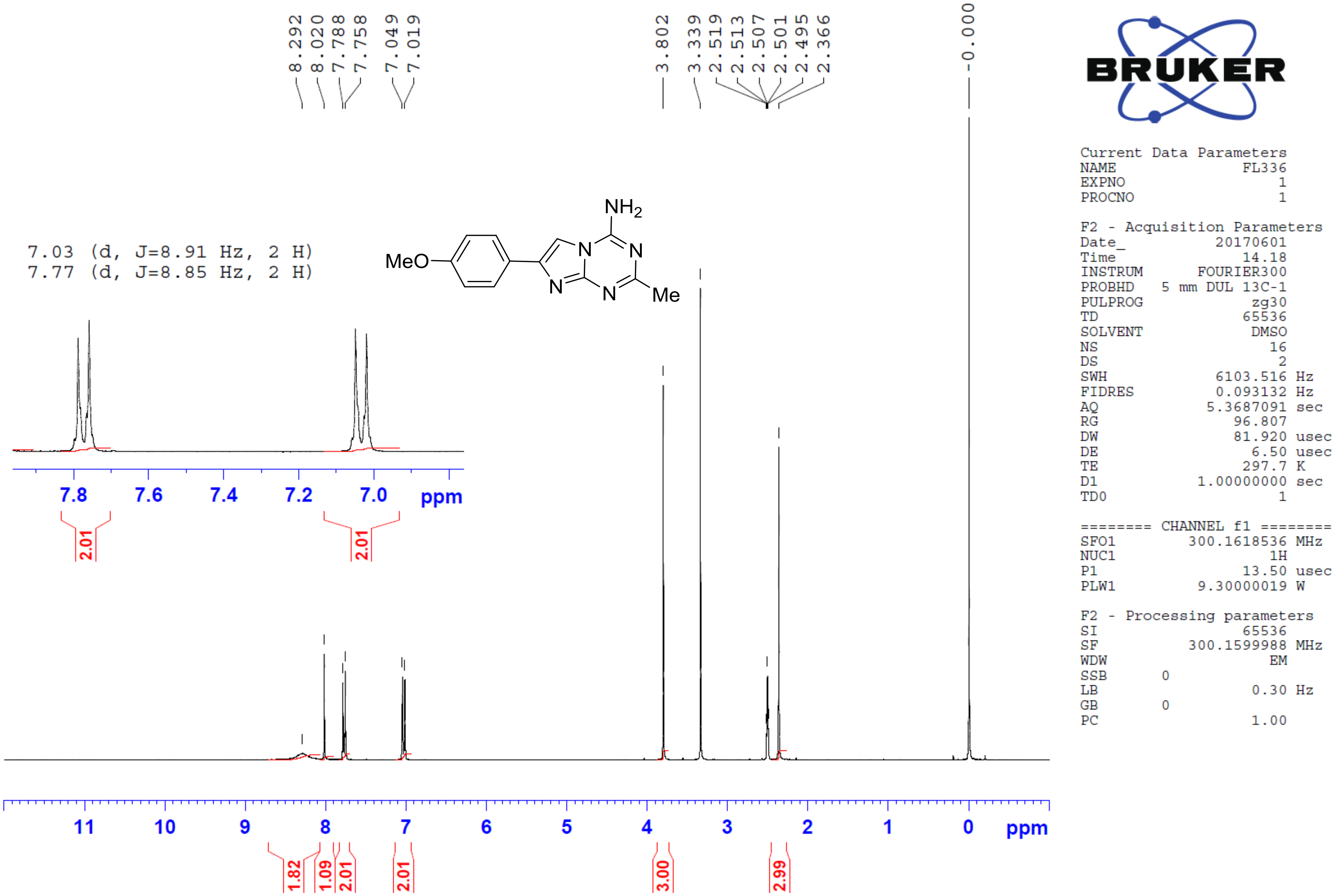

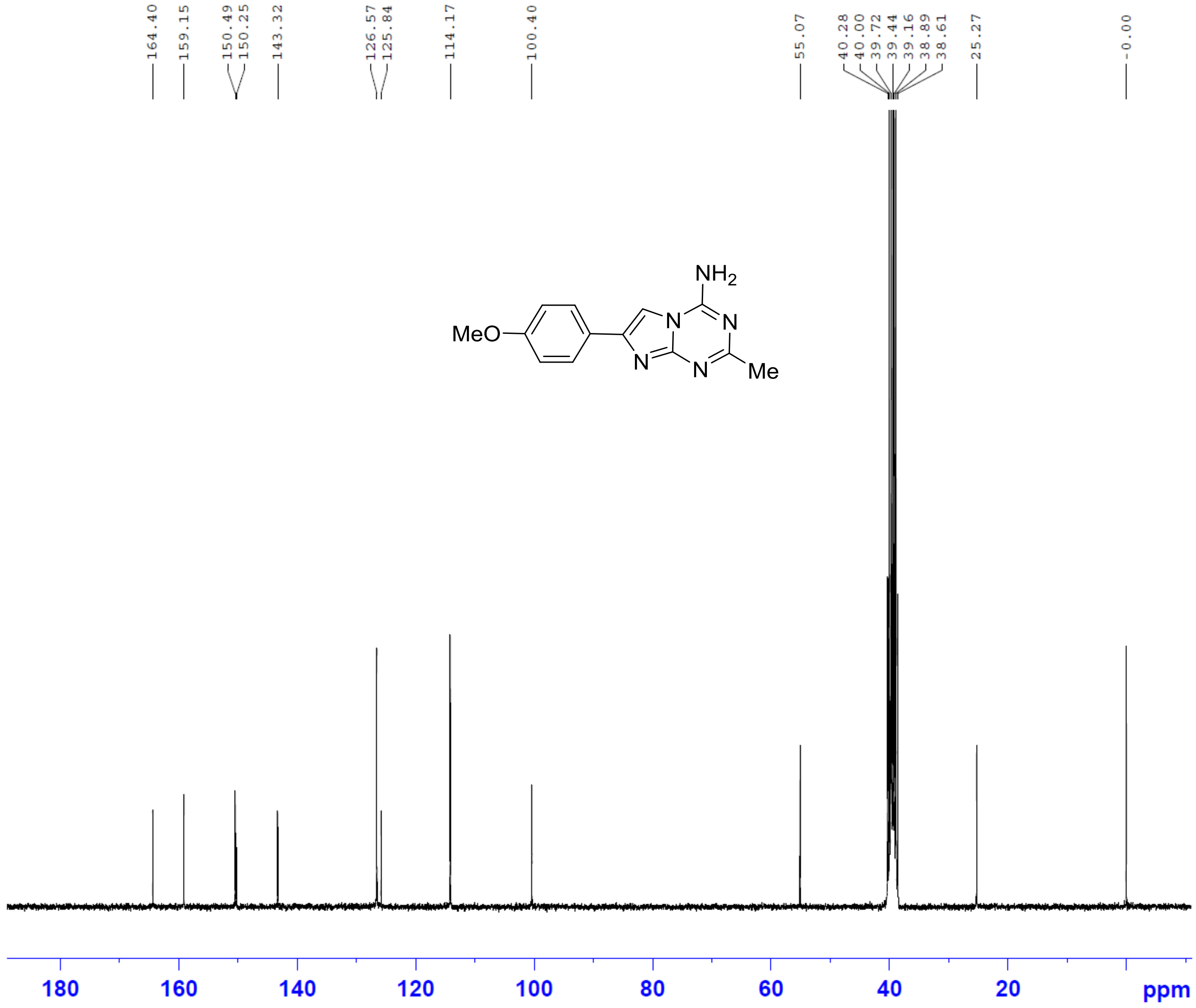

Current Data Parameters

NAME	FL336
EXPNO	2
PROCNO	1

F2 - Acquisition Parameters

Date_	20170606
Time	16.42
INSTRUM	FOURIER300
PROBHD	5 mm DUL 13C-1
PULPROG	zgpg30
TD	65536
SOLVENT	DMSO
NS	15360
DS	4
SWH	24414.063 Hz
FIDRES	0.372529 Hz
AQ	1.3421773 sec
RG	501.187
DW	20.480 usec
DE	6.50 usec
TE	300.1 K
D1	2.00000000 sec
D11	0.03000000 sec
D31	0.00001500 sec
D40	0.00439029 sec
L4	37
L5	53
P32	98.00 usec
TD0	15

======== CHANNEL f1 ========

SFO1	75.4828392 MHz
NUC1	13C
P1	15.00 usec
PLW1	22.00000000 W

======== CHANNEL f2 ========

SFO2	300.1612006 MHz
NUC2	1H
CPDPRG[2	waltz16
PCPD2	98.00 usec
PLW2	9.30000019 W
PLW12	0.29359001 W
PLW13	0.20359001 W

F2 - Processing parameters

SI	32768
SF	75.4753342 MHz
WDW	EM
SSB	0
LB	1.00 Hz
GB	0
PC	1.40

4-Amino-2-methyl-7-(4-biphenyl)imidazo[1,2-a][1,3,5]triazine (5f)

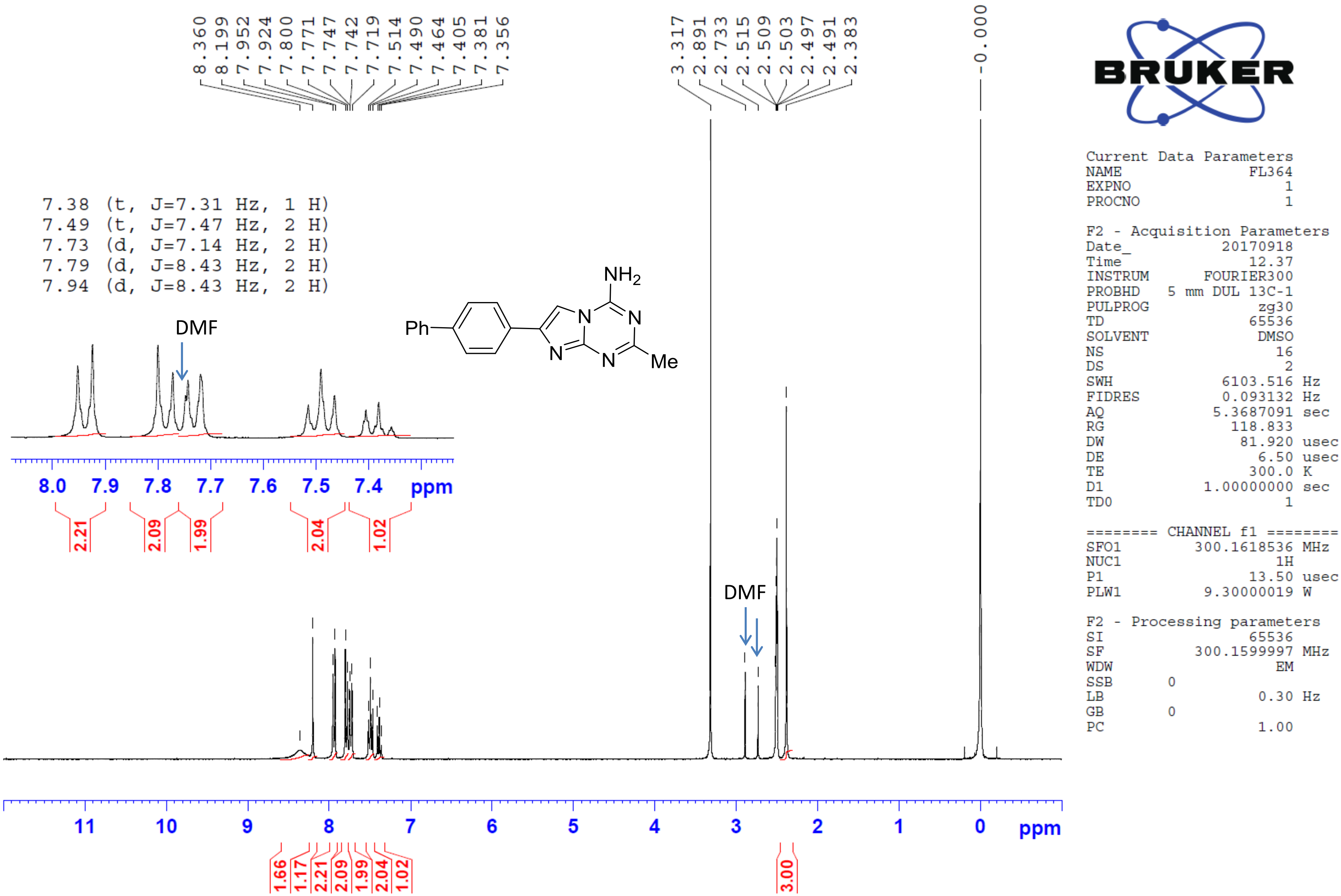

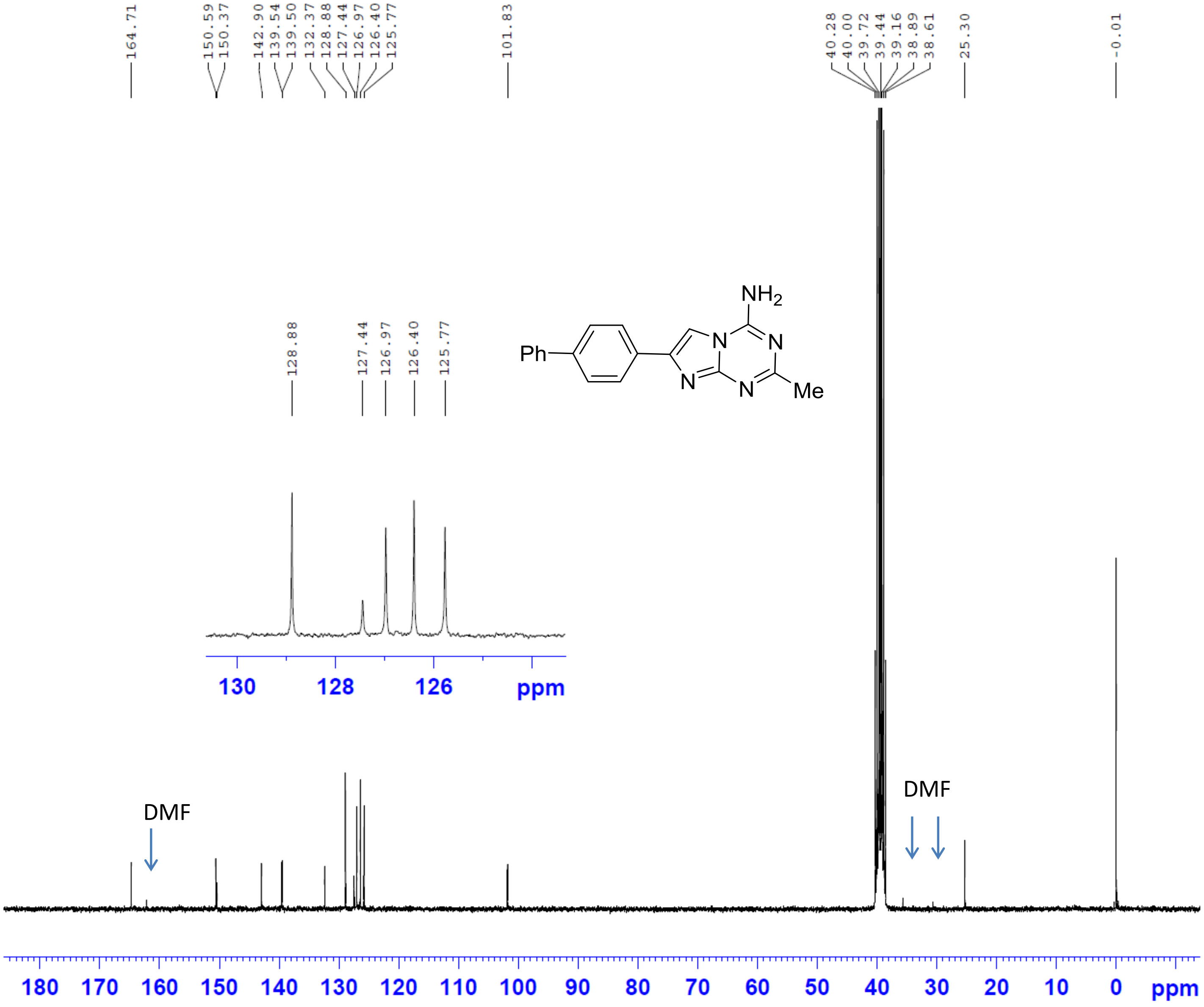


```
Current Data Parameters
NAME                FL364
EXPNO                   2
PROCNO                  1

F2 - Acquisition Parameters
Date_            20170919
Time                18.06
INSTRUM       FOURIER300
PROBHD   5 mm DUL 13C-1
PULPROG           zgpg30
TD                 65536
SOLVENT             DMSO
NS                 15360
DS                     4
SWH            24414.063 Hz
FIDRES          0.372529 Hz
AQ             1.3421773 sec
RG               501.187
DW                20.480 usec
DE                  6.50 usec
TE                 300.1 K
D1            2.00000000 sec
D11           0.03000000 sec
D31           0.00001500 sec
D40           0.00439029 sec
L4                    37
L5                    53
P32                98.00 usec
TD0                   15

======== CHANNEL f1 ========
SFO1          75.4828392 MHz
NUC1                 13C
P1                 15.00 usec
PLW1         22.00000000 W

======== CHANNEL f2 ========
SFO2         300.1612006 MHz
NUC2                  1H
CPDPRG[2         waltz16
PCPD2              98.00 usec
PLW2          9.30000019 W
PLW12         0.29359001 W
PLW13         0.20359001 W

F2 - Processing parameters
SI                 32768
SF            75.4753350 MHz
WDW                   EM
SSB       0
LB                  1.00 Hz
GB        0
PC                  1.40
```

4-Amino-2-ethyl-7-phenylimidazo[1,2-a][1,3,5]triazine (5g)

```
1.25 (t, J=7.55 Hz, 3 H)
2.66 (q, J=7.54 Hz, 2 H)
7.35 (t, J=7.37 Hz, 1 H)
7.47 (t, J=7.50 Hz, 2 H)
7.86 (d, J=7.02 Hz, 2 H)
```

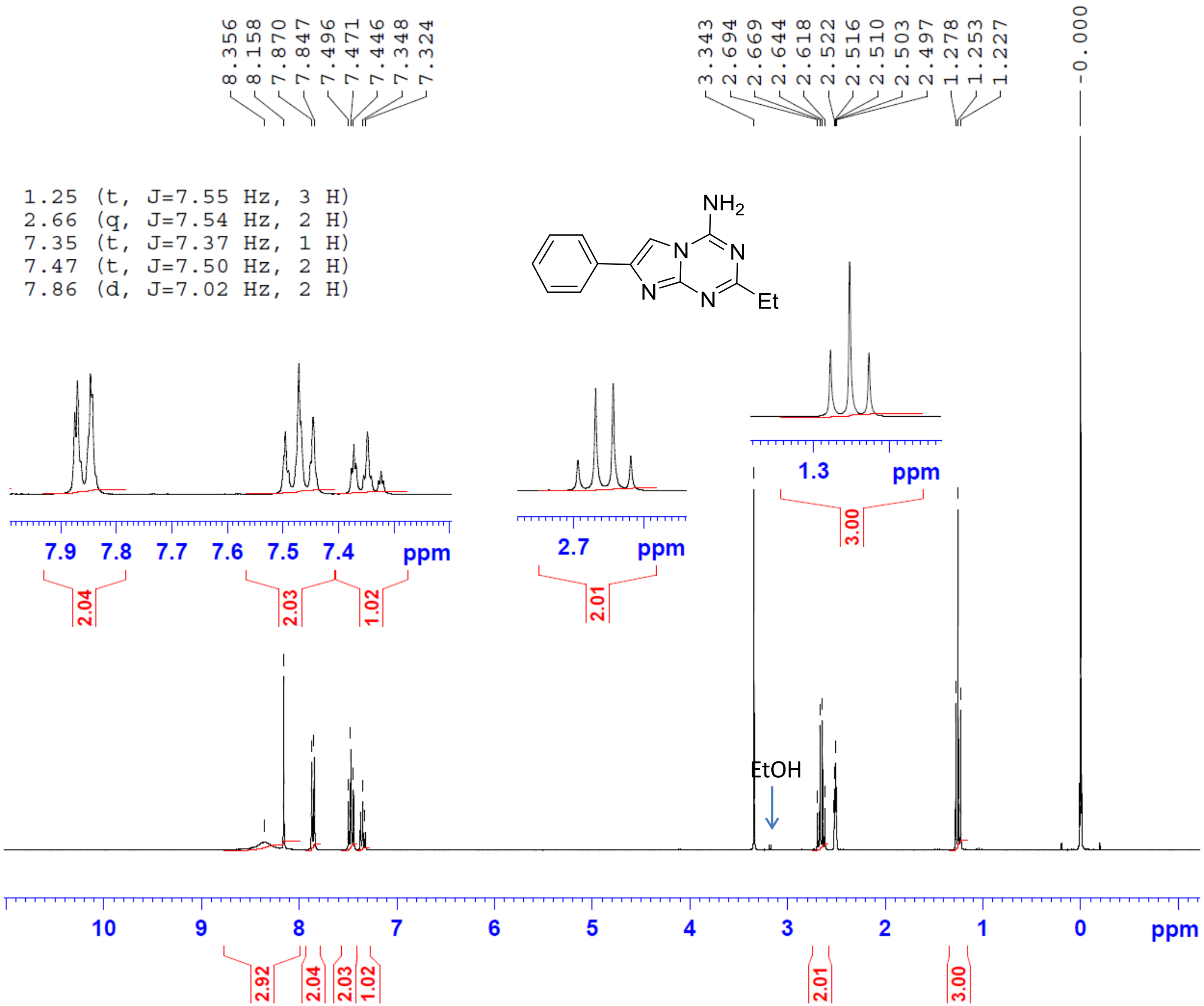


```
Current Data Parameters
NAME            FL328
EXPNO               2
PROCNO              1

F2 - Acquisition Parameters
Date_        20170529
Time            15.05
INSTRUM    FOURIER300
PROBHD   5 mm DUL 13C-1
PULPROG          zg30
TD              65536
SOLVENT          DMSO
NS                 16
DS                  2
SWH          6103.516 Hz
FIDRES       0.093132 Hz
AQ          5.3687091 sec
RG            79.5788
DW             81.920 usec
DE               6.50 usec
TE              297.7 K
D1         1.00000000 sec
TD0                 1

======== CHANNEL f1 ========
SFO1      300.1618536 MHz
NUC1               1H
P1              13.50 usec
PLW1       9.30000019 W

F2 - Processing parameters
SI              65536
SF        300.1599979 MHz
WDW                EM
SSB      0
LB               0.30 Hz
GB       0
PC               1.00
```

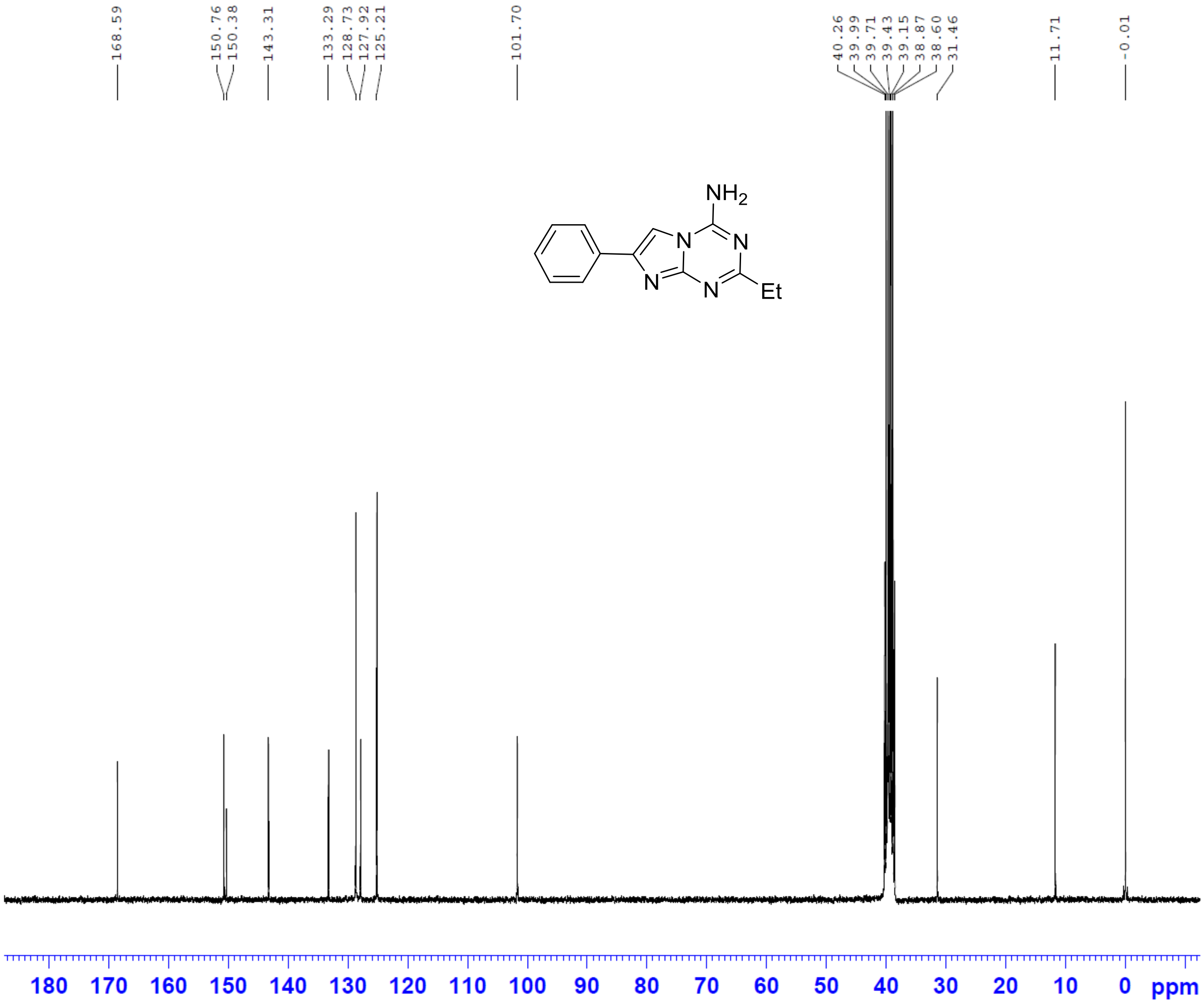


```
Current Data Parameters
NAME                FL328
EXPNO                   3
PROCNO                  1

F2 - Acquisition Parameters
Date_            20170530
Time                19.05
INSTRUM        FOURIER300
PROBHD    5 mm DUL 13C-1
PULPROG           zgpg30
TD                  65536
SOLVENT              DMSO
NS                  14336
DS                      4
SWH             24414.063 Hz
FIDRES           0.372529 Hz
AQ              1.3421773 sec
RG                501.187
DW                 20.480 usec
DE                   6.50 usec
TE                  297.7 K
D1             2.00000000 sec
D11            0.03000000 sec
D31            0.00001500 sec
D40            0.00439029 sec
L4                     37
L5                     53
P32                 98.00 usec
TD0                    14

======== CHANNEL f1 ========
SFO1           75.4828392 MHz
NUC1                  13C
P1                  15.00 usec
PLW1          22.00000000 W

======== CHANNEL f2 ========
SFO2          300.1612006 MHz
NUC2                   1H
CPDPRG[2          waltz16
PCPD2               98.00 usec
PLW2           9.30000019 W
PLW12          0.29359001 W
PLW13          0.20359001 W

F2 - Processing parameters
SI                  32768
SF             75.4753342 MHz
WDW                    EM
SSB       0
LB                   1.00 Hz
GB        0
PC                   1.40
```

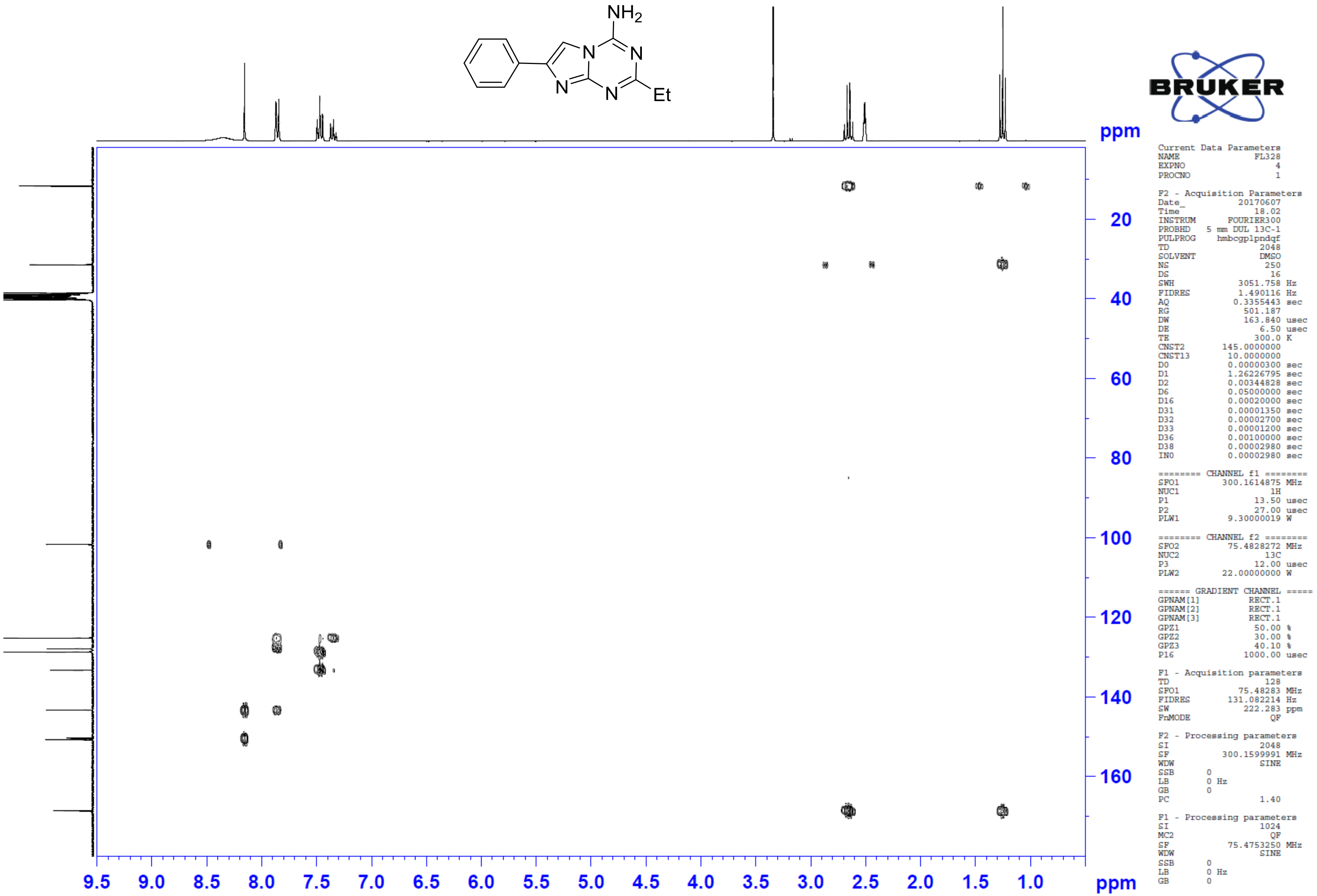
NH2
N
N
N
N
Et
ppm
20
40
60
80
100
120
140
160
9.5
9.0
8.5
8.0
7.5
7.0
6.5
6.0
5.5
5.0
4.5
4.0
3.5
3.0
2.5
2.0
1.5
1.0
ppm
Current Data Parameters
NAME FL328
EXPNO 4
PROCNO 1
F2 - Acquisition Parameters
Date_ 20170607
Time 18.02
INSTRUM FOURIER300
PROBHD 5 mm DUL 13C-1
PULPROG hmbcgplpndqf
TD 2048
SOLVENT DMSO
NS 250
DS 16
SWH 3051.758 Hz
FIDRES 1.490116 Hz
AQ 0.3355443 sec
RG 501.187
DW 163.840 usec
DE 6.50 usec
TE 300.0 K
CNST2 145.0000000
CNST13 10.0000000
D0 0.00000300 sec
D1 1.26226795 sec
D2 0.00344828 sec
D6 0.05000000 sec
D16 0.00020000 sec
D31 0.00001350 sec
D32 0.00002700 sec
D33 0.00001200 sec
D36 0.00100000 sec
D38 0.00002980 sec
IN0 0.00002980 sec
======== CHANNEL f1 ========
SFO1 300.1614875 MHz
NUC1 1H
P1 13.50 usec
P2 27.00 usec
PLW1 9.30000019 W
======== CHANNEL f2 ========
SFO2 75.4828272 MHz
NUC2 13C
P3 12.00 usec
PLW2 22.00000000 W
====== GRADIENT CHANNEL =====
GPNAM[1] RECT.1
GPNAM[2] RECT.1
GPNAM[3] RECT.1
GPZ1 50.00 %
GPZ2 30.00 %
GPZ3 40.10 %
P16 1000.00 usec
F1 - Acquisition parameters
TD 128
SFO1 75.48283 MHz
FIDRES 131.082214 Hz
SW 222.283 ppm
FnMODE QF
F2 - Processing parameters
SI 2048
SF 300.1599991 MHz
WDW SINE
SSB 0
LB 0 Hz
GB 0
PC 1.40
F1 - Processing parameters
SI 1024
MC2 QF
SF 75.4753250 MHz
WDW SINE
SSB 0
LB 0 Hz
GB 0

4-Amino-2-ethyl-7-(4-fluorophenyl)imidazo[1,2-a][1,3,5]triazine (5h)

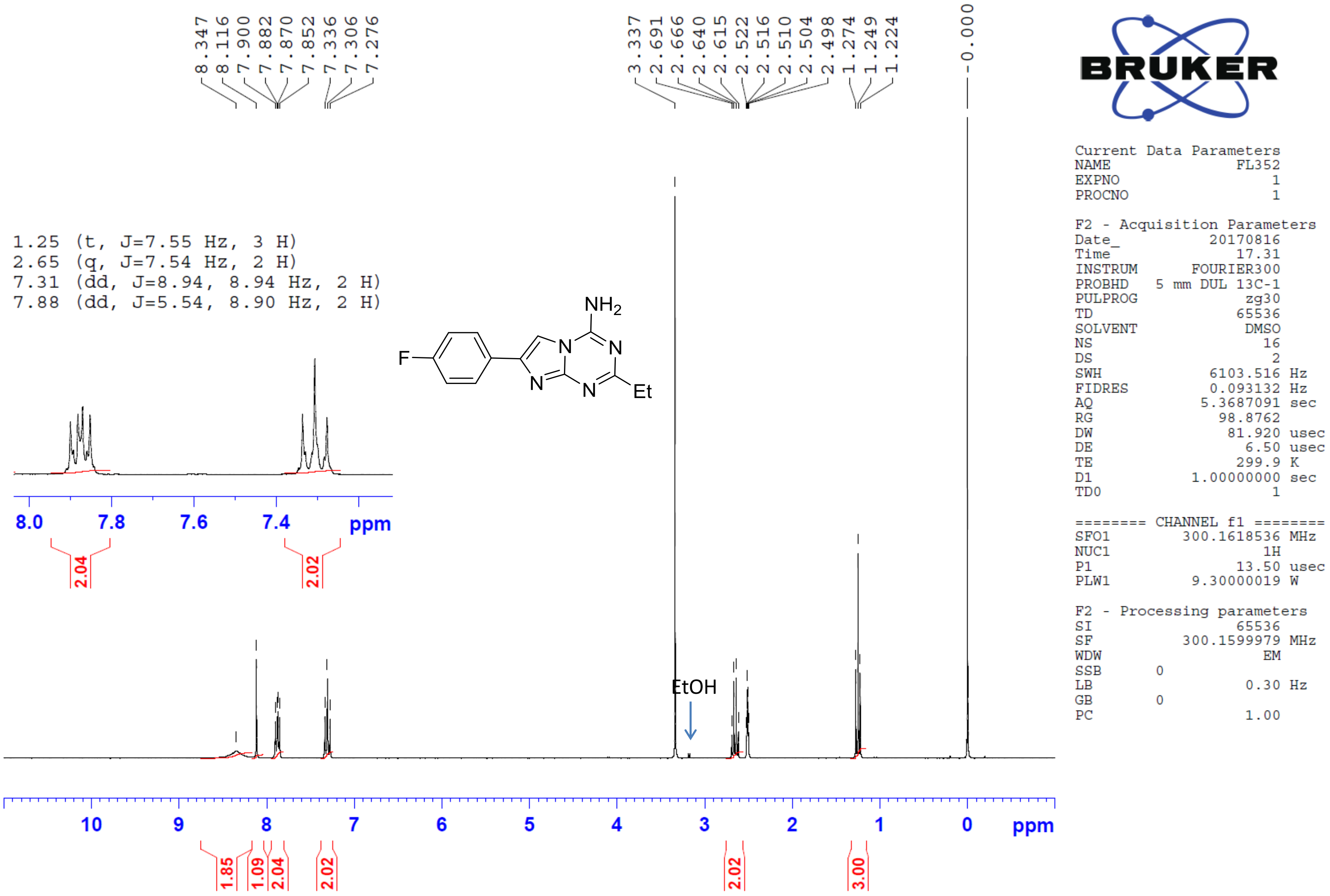

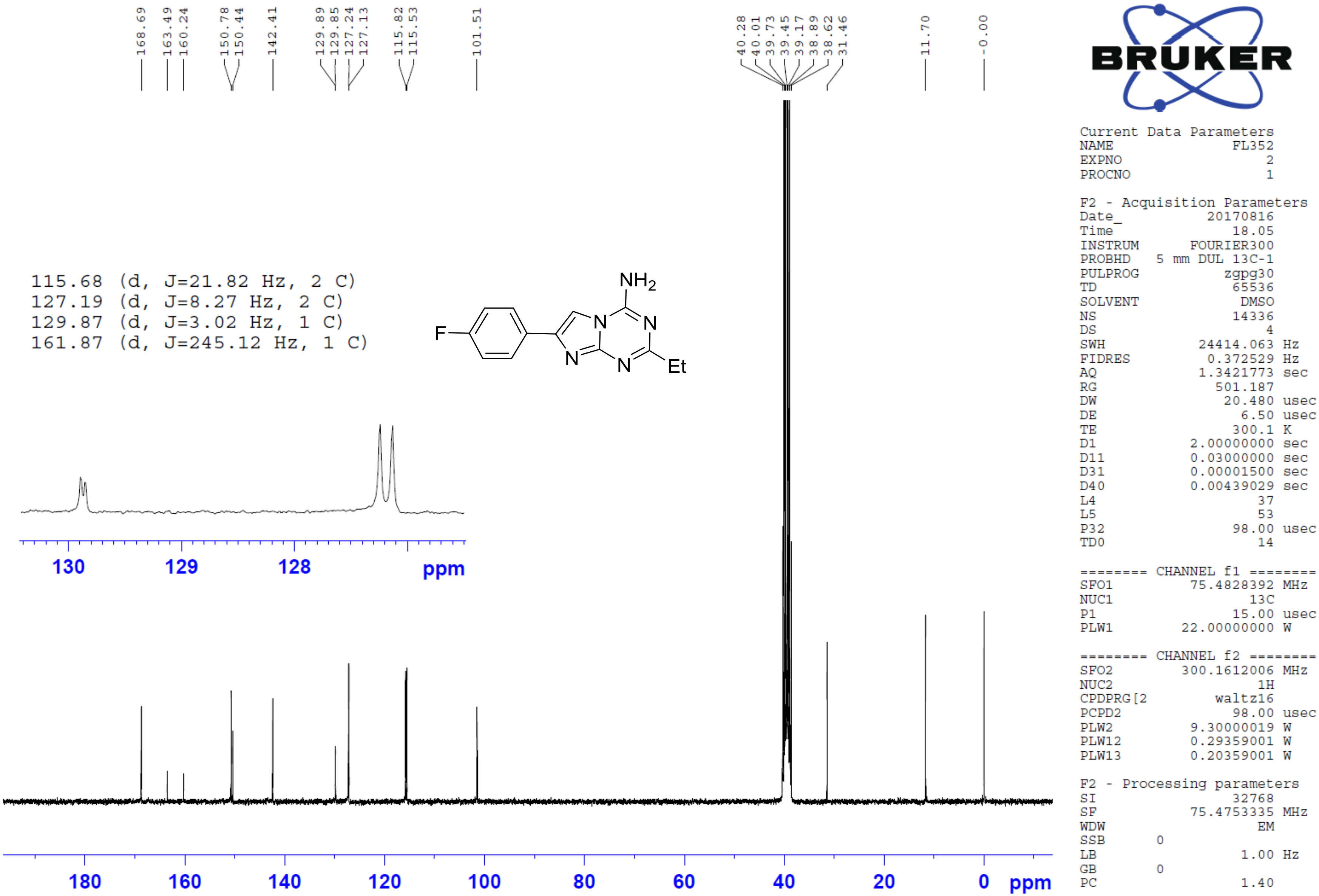
168.69
163.49
160.24
150.78
150.44
142.41
129.89
129.85
127.24
127.13
115.82
115.53
101.51
40.28
40.01
39.73
39.45
39.17
38.89
38.62
31.46
11.70
-0.00
115.68 (d, J=21.82 Hz, 2 C)
127.19 (d, J=8.27 Hz, 2 C)
129.87 (d, J=3.02 Hz, 1 C)
161.87 (d, J=245.12 Hz, 1 C)
F
NH2
N
N
N
N
Et
130
129
128
ppm
180
160
140
120
100
80
60
40
20
0
ppm
BRUKER
Current Data Parameters
NAME FL352
EXPNO 2
PROCNO 1
F2 - Acquisition Parameters
Date_ 20170816
Time 18.05
INSTRUM FOURIER300
PROBHD 5 mm DUL 13C-1
PULPROG zgpg30
TD 65536
SOLVENT DMSO
NS 14336
DS 4
SWH 24414.063 Hz
FIDRES 0.372529 Hz
AQ 1.3421773 sec
RG 501.187
DW 20.480 usec
DE 6.50 usec
TE 300.1 K
D1 2.00000000 sec
D11 0.03000000 sec
D31 0.00001500 sec
D40 0.00439029 sec
L4 37
L5 53
P32 98.00 usec
TD0 14
======== CHANNEL f1 ========
SFO1 75.4828392 MHz
NUC1 13C
P1 15.00 usec
PLW1 22.00000000 W
======== CHANNEL f2 ========
SFO2 300.1612006 MHz
NUC2 1H
CPDPRG[2 waltz16
PCPD2 98.00 usec
PLW2 9.30000019 W
PLW12 0.29359001 W
PLW13 0.20359001 W
F2 - Processing parameters
SI 32768
SF 75.4753335 MHz
WDW EM
SSB 0
LB 1.00 Hz
GB 0
PC 1.40

4-Amino-2-ethyl-7-(4-chlorophenyl)imidazo[1,2-a][1,3,5]triazine (5i)

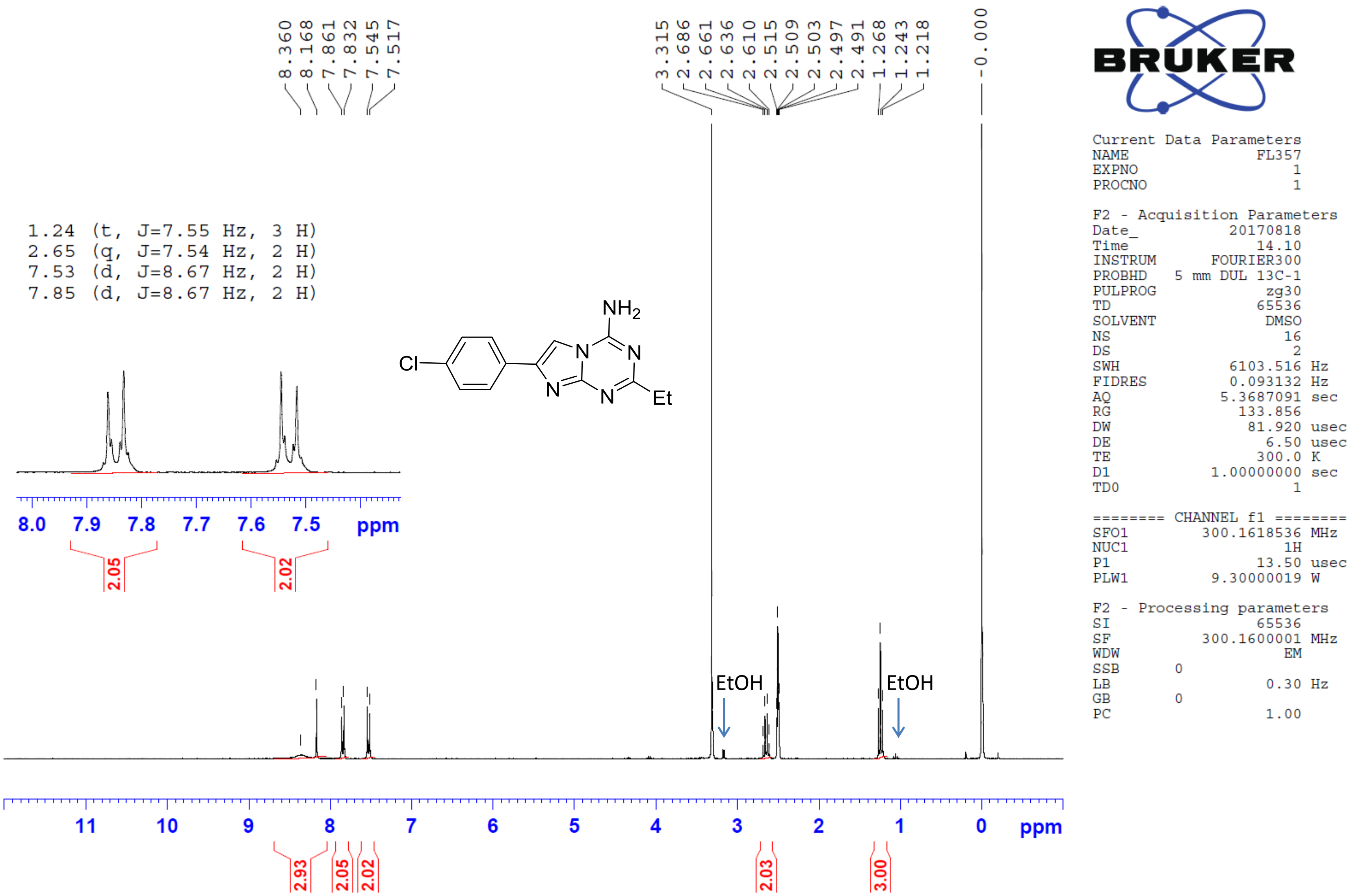

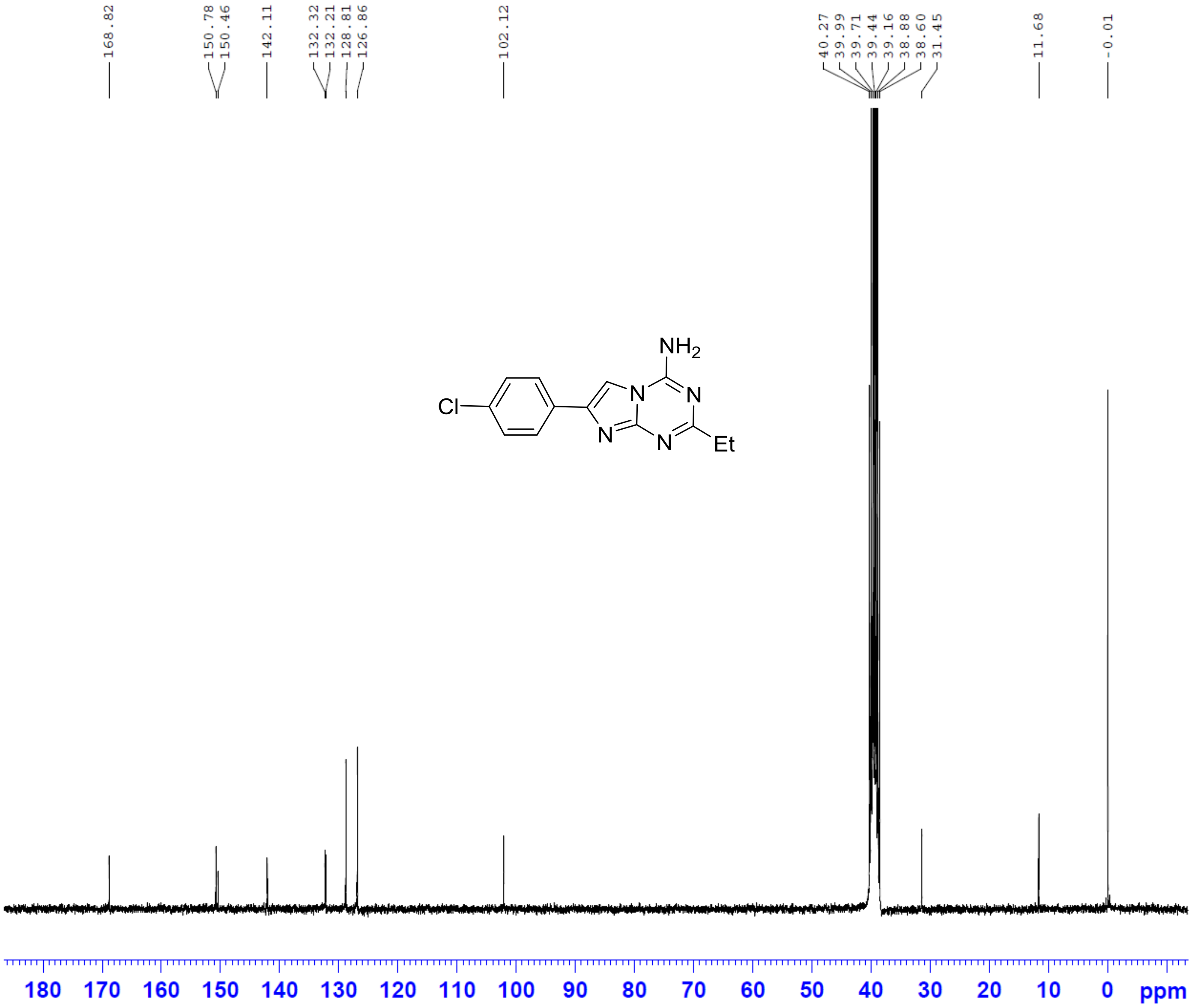

Current Data Parameters
NAME FL357
EXPNO 2
PROCNO 1

F2 - Acquisition Parameters
Date_ 20170820
Time 14.26
INSTRUM FOURIER300
PROBHD 5 mm DUL 13C-1
PULPROG zgpg30
TD 65536
SOLVENT DMSO
NS 20480
DS 4
SWH 24414.063 Hz
FIDRES 0.372529 Hz
AQ 1.3421773 sec
RG 501.187
DW 20.480 usec
DE 6.50 usec
TE 300.1 K
D1 2.00000000 sec
D11 0.03000000 sec
D31 0.00001500 sec
D40 0.00439029 sec
L4 37
L5 53
P32 98.00 usec
TD0 20

======== CHANNEL f1 ========
SFO1 75.4828392 MHz
NUC1 13C
P1 15.00 usec
PLW1 22.00000000 W

======== CHANNEL f2 ========
SFO2 300.1612006 MHz
NUC2 1H
CPDPRG[2 waltz16
PCPD2 98.00 usec
PLW2 9.30000019 W
PLW12 0.29359001 W
PLW13 0.20359001 W

F2 - Processing parameters
SI 32768
SF 75.4753350 MHz
WDW EM
SSB 0
LB 1.00 Hz
GB 0
PC 1.40

4-Amino-2-ethyl-7-(4-methylphenyl)imidazo[1,2-a][1,3,5]triazine (5j)

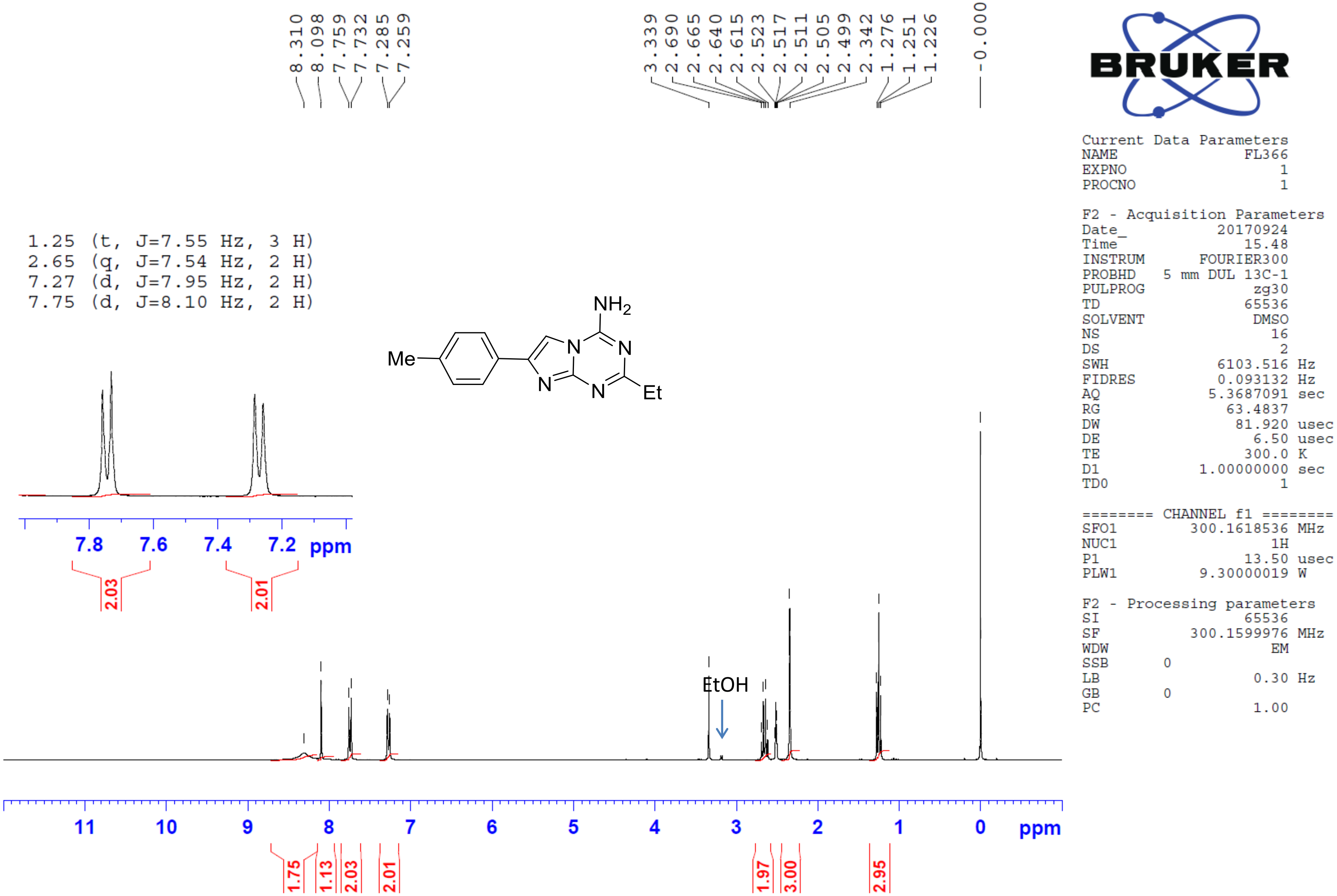

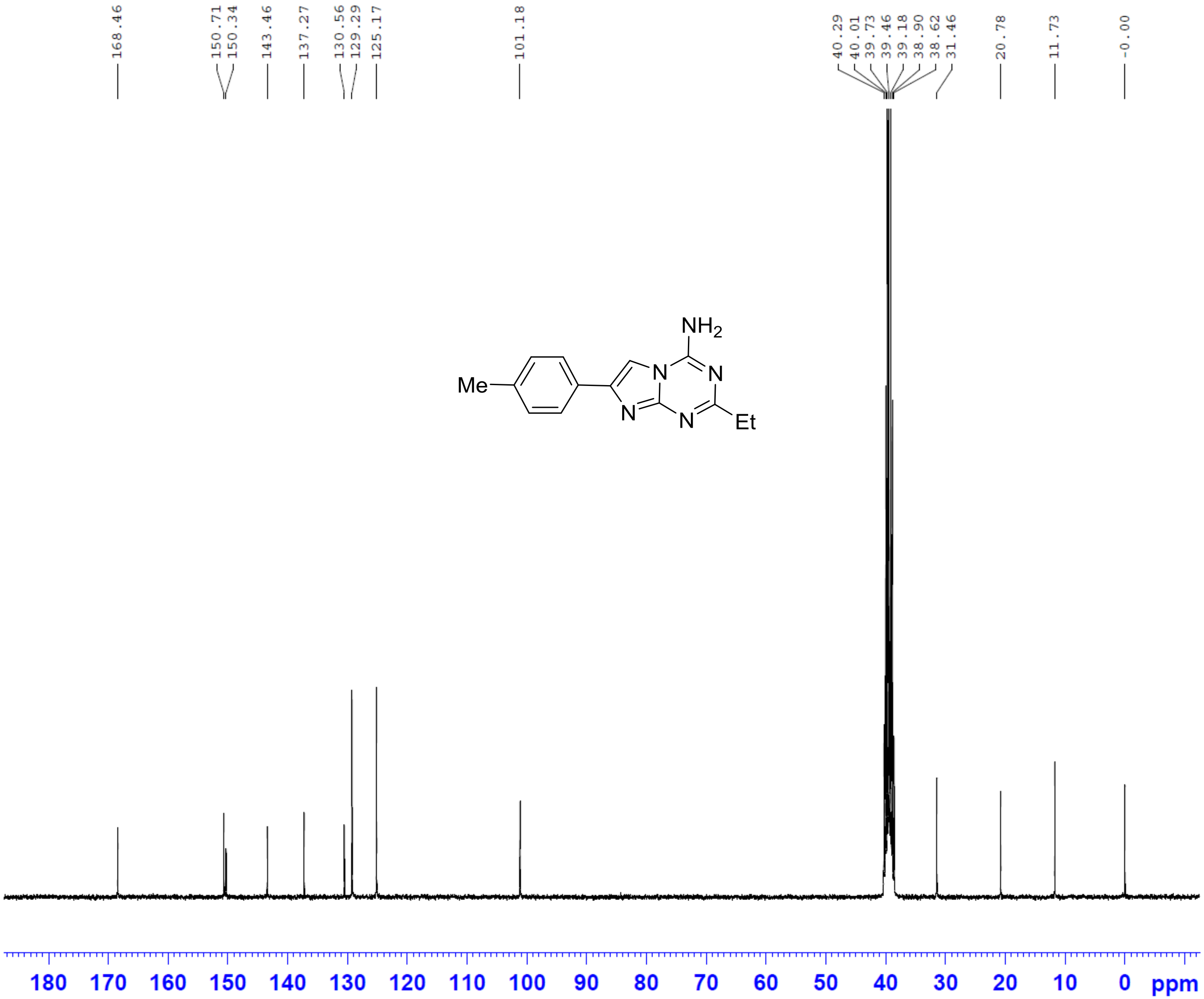

BRUKER

Current Data Parameters
NAME FL366
EXPNO 2
PROCNO 1

F2 - Acquisition Parameters
Date_ 20170924
Time 16.10
INSTRUM FOURIER300
PROBHD 5 mm DUL 13C-1
PULPROG zgpg30
TD 65536
SOLVENT DMSO
NS 9216
DS 4
SWH 24414.063 Hz
FIDRES 0.372529 Hz
AQ 1.3421773 sec
RG 501.187
DW 20.480 usec
DE 6.50 usec
TE 300.1 K
D1 2.00000000 sec
D11 0.03000000 sec
D31 0.00001500 sec
D40 0.00439029 sec
L4 37
L5 53
P32 98.00 usec
TD0 9

======== CHANNEL f1 ========
SFO1 75.4828392 MHz
NUC1 13C
P1 15.00 usec
PLW1 22.00000000 W

======== CHANNEL f2 ========
SFO2 300.1612006 MHz
NUC2 1H
CPDPRG[2 waltz16
PCPD2 98.00 usec
PLW2 9.30000019 W
PLW12 0.29359001 W
PLW13 0.20359001 W

F2 - Processing parameters
SI 32768
SF 75.4753335 MHz
WDW EM
SSB 0
LB 1.00 Hz
GB 0
PC 1.40

4-Amino-2-ethyl-7-(4-methoxyphenyl)imidazo[1,2-a][1,3,5]triazine (5k)

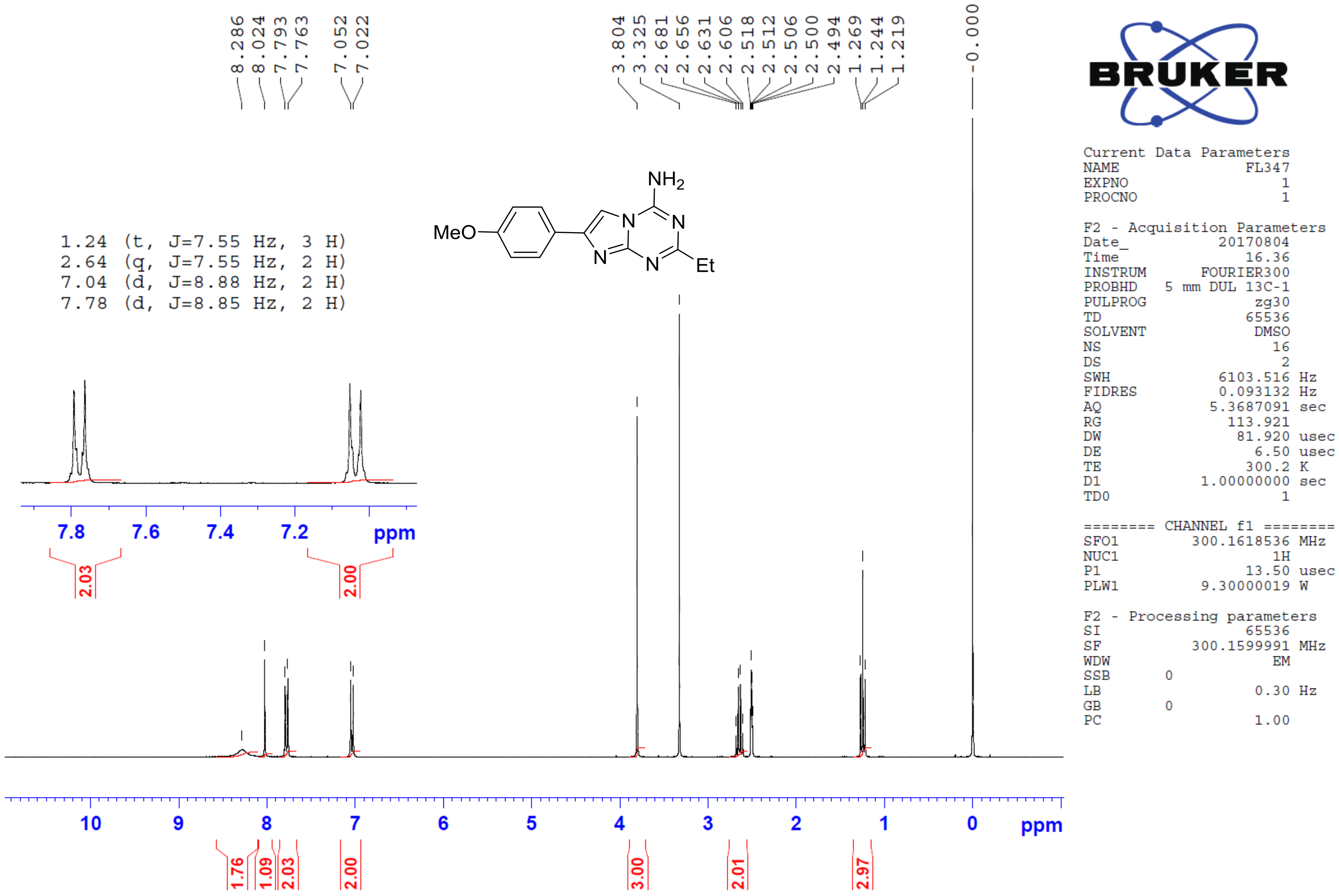

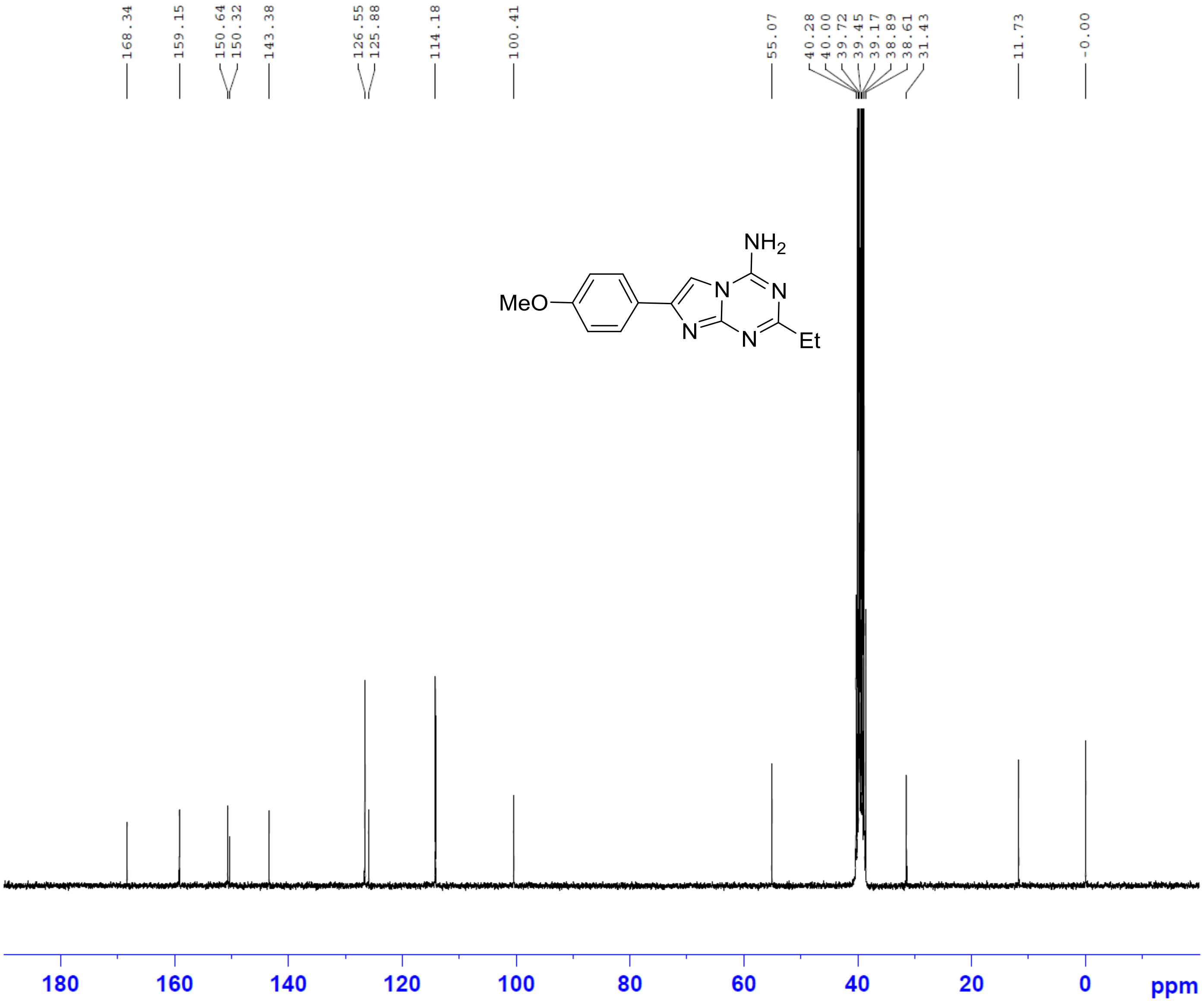

BRUKER

```
Current Data Parameters
NAME                  FL347
EXPNO                     2
PROCNO                    1

F2 - Acquisition Parameters
Date_              20170804
Time                  18.05
INSTRUM          FOURIER300
PROBHD   5 mm DUL 13C-1
PULPROG             zgpg30
TD                    65536
SOLVENT                DMSO
NS                    14336
DS                        4
SWH              24414.063 Hz
FIDRES            0.372529 Hz
AQ               1.3421773 sec
RG                 501.187
DW                  20.480 usec
DE                    6.50 usec
TE                   300.2 K
D1              2.00000000 sec
D11             0.03000000 sec
D31             0.00001500 sec
D40             0.00439029 sec
L4                       37
L5                       53
P32                  98.00 usec
TD0                      14

======== CHANNEL f1 ========
SFO1           75.4828392 MHz
NUC1                   13C
P1                   15.00 usec
PLW1           22.00000000 W

======== CHANNEL f2 ========
SFO2          300.1612006 MHz
NUC2                    1H
CPDPRG[2           waltz16
PCPD2                98.00 usec
PLW2            9.30000019 W
PLW12           0.29359001 W
PLW13           0.20359001 W

F2 - Processing parameters
SI                    32768
SF               75.4753343 MHz
WDW                      EM
SSB       0
LB                     1.00 Hz
GB        0
PC                     1.40
```

4-Amino-2-ethyl-7-(4-biphenyl)imidazo[1,2-a][1,3,5]triazine (5l)

1.26 (t, J=7.55 Hz, 3 H)
2.66 (q, J=7.55 Hz, 2 H)
7.38 (t, J=7.29 Hz, 1 H)
7.49 (t, J=7.50 Hz, 2 H)
7.73 (d, J=7.23 Hz, 2 H)
7.79 (d, J=8.37 Hz, 2 H)
7.95 (d, J=8.28 Hz, 2 H)

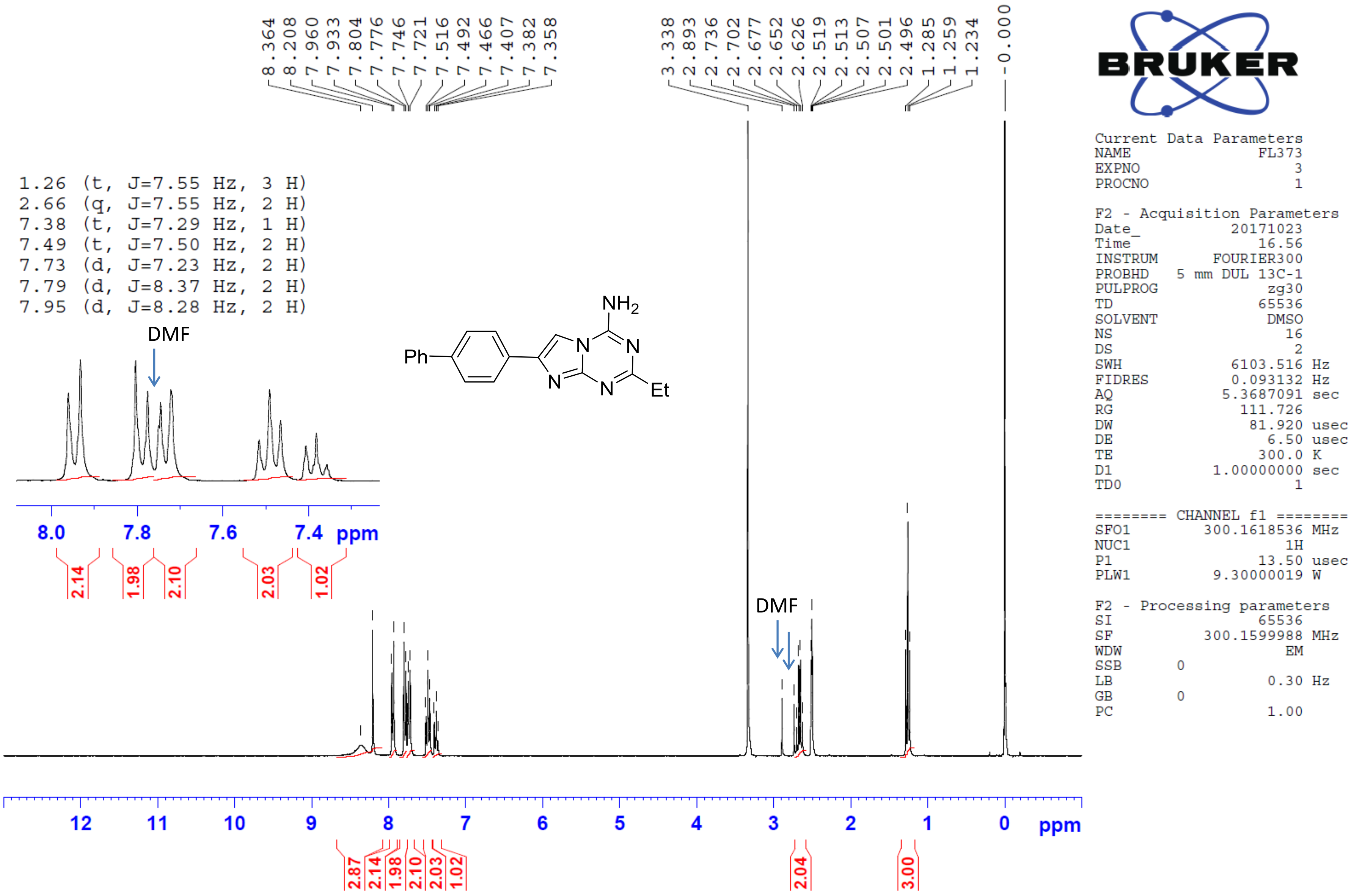

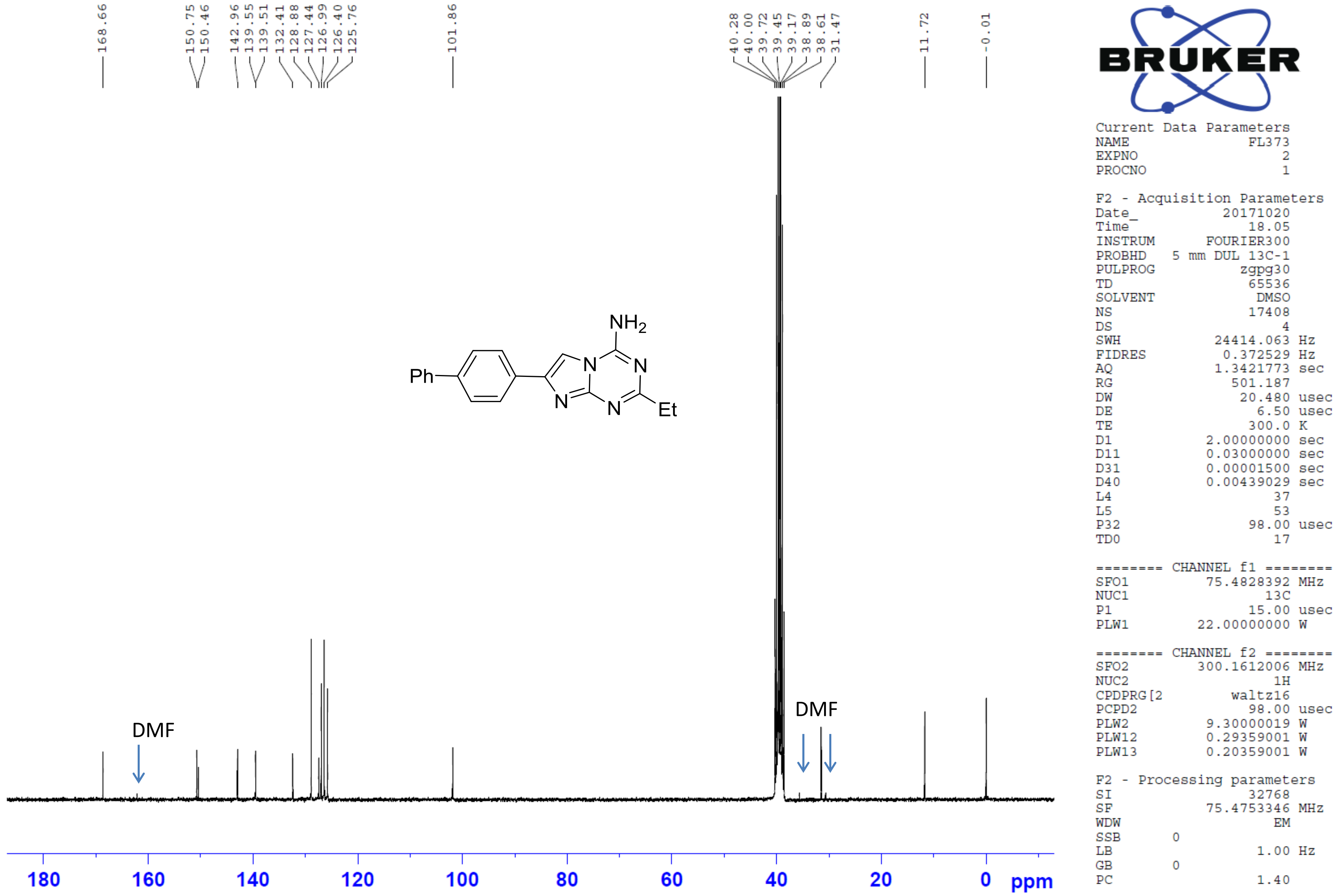
168.66
150.75
150.46
142.96
139.55
139.51
132.41
128.88
127.44
126.99
126.40
125.76
101.86
40.28
40.00
39.72
39.45
39.17
38.89
38.61
31.47
11.72
-0.01
NH2
Ph
N
N
N
N
Et
DMF
DMF
180
160
140
120
100
80
60
40
20
0
ppm
BRUKER
Current Data Parameters
NAME FL373
EXPNO 2
PROCNO 1
F2 - Acquisition Parameters
Date_ 20171020
Time 18.05
INSTRUM FOURIER300
PROBHD 5 mm DUL 13C-1
PULPROG zgpg30
TD 65536
SOLVENT DMSO
NS 17408
DS 4
SWH 24414.063 Hz
FIDRES 0.372529 Hz
AQ 1.3421773 sec
RG 501.187
DW 20.480 usec
DE 6.50 usec
TE 300.0 K
D1 2.00000000 sec
D11 0.03000000 sec
D31 0.00001500 sec
D40 0.00439029 sec
L4 37
L5 53
P32 98.00 usec
TD0 17
======== CHANNEL f1 ========
SFO1 75.4828392 MHz
NUC1 13C
P1 15.00 usec
PLW1 22.00000000 W
======== CHANNEL f2 ========
SFO2 300.1612006 MHz
NUC2 1H
CPDPRG[2 waltz16
PCPD2 98.00 usec
PLW2 9.30000019 W
PLW12 0.29359001 W
PLW13 0.20359001 W
F2 - Processing parameters
SI 32768
SF 75.4753346 MHz
WDW EM
SSB 0
LB 1.00 Hz
GB 0
PC 1.40

4-Amino-2-propyl-7-phenylimidazo[1,2-a][1,3,5]triazine (5m)

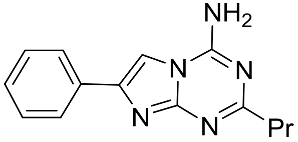

0.94 (t, J=7.38 Hz, 3 H)
1.77 (m, J=7.40 Hz, 2 H)
2.60 (t, J=7.40 Hz, 2 H)
7.35 (t, J=7.34 Hz, 1 H)
7.47 (t, J=7.52 Hz, 2 H)
7.85 (d, J=7.08 Hz, 2 H)

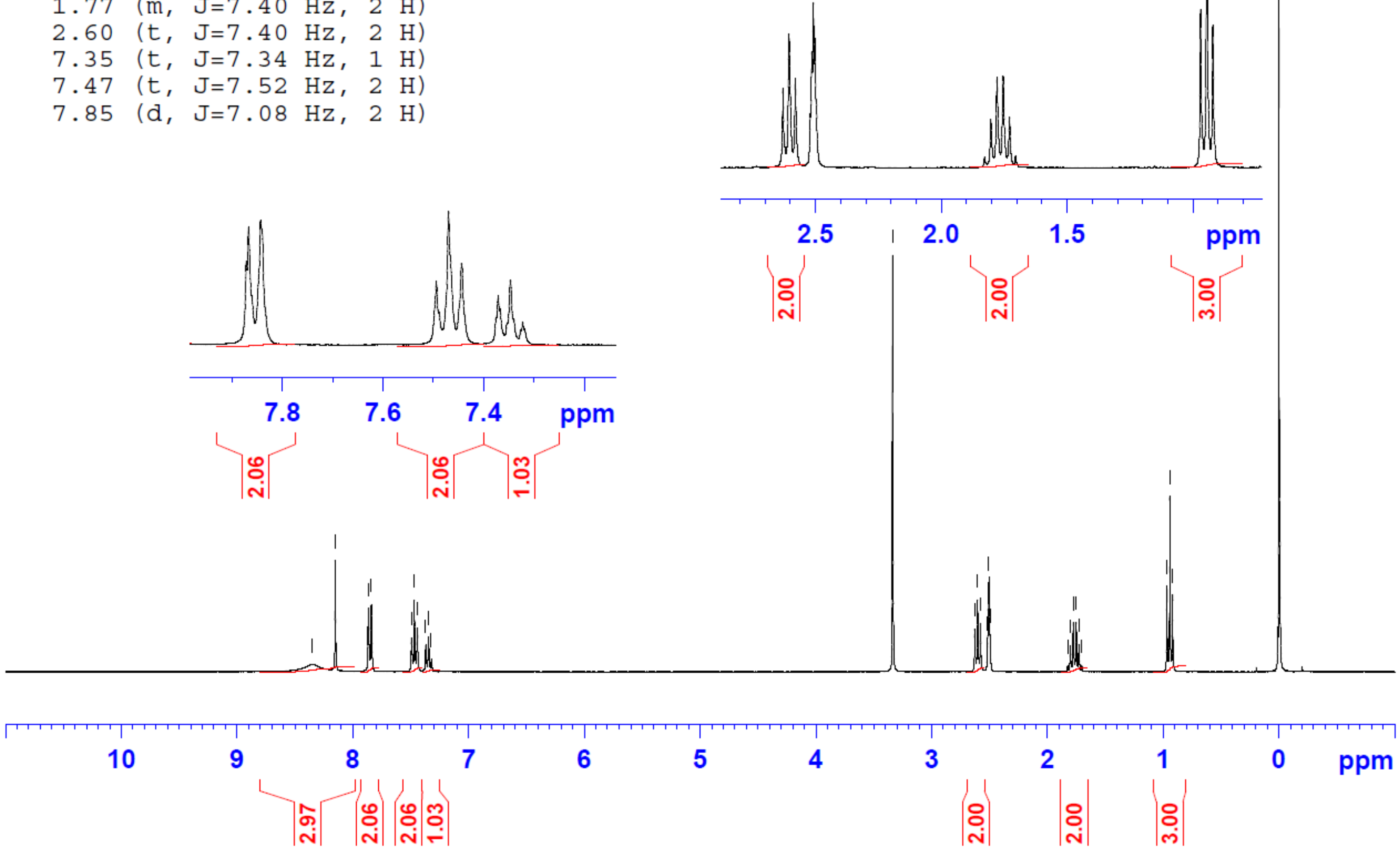

BRUKER

Current Data Parameters
NAME FL334
EXPNO 1
PROCNO 1

F2 - Acquisition Parameters
Date_ 20170601
Time 14.09
INSTRUM FOURIER300
PROBHD 5 mm DUL 13C-1
PULPROG zg30
TD 65536
SOLVENT DMSO
NS 16
DS 2
SWH 6103.516 Hz
FIDRES 0.093132 Hz
AQ 5.3687091 sec
RG 88.2542
DW 81.920 usec
DE 6.50 usec
TE 297.7 K
D1 1.00000000 sec
TD0 1

======== CHANNEL f1 ========
SFO1 300.1618536 MHz
NUC1 1H
P1 13.50 usec
PLW1 9.30000019 W

F2 - Processing parameters
SI 65536
SF 300.1599986 MHz
WDW EM
SSB 0
LB 0.30 Hz
GB 0
PC 1.00

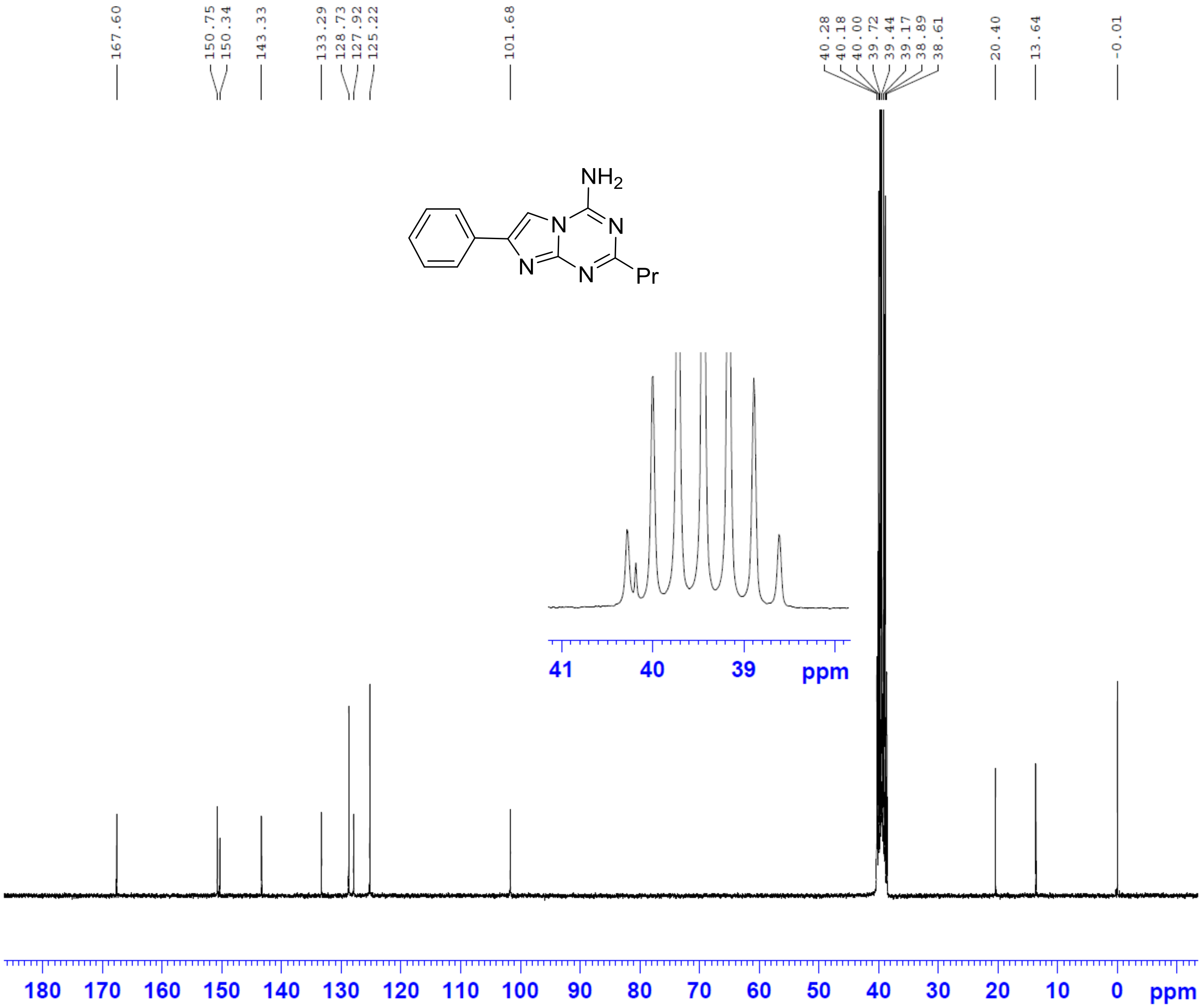

Current Data Parameters

NAME	FL334
EXPNO	2
PROCNO	1

F2 - Acquisition Parameters

Date_	20170610
Time	18.06
INSTRUM	FOURIER300
PROBHD	5 mm DUL 13C-1
PULPROG	zgpg30
TD	65536
SOLVENT	DMSO
NS	15360
DS	4
SWH	24414.063 Hz
FIDRES	0.372529 Hz
AQ	1.3421773 sec
RG	501.187
DW	20.480 usec
DE	6.50 usec
TE	300.1 K
D1	2.00000000 sec
D11	0.03000000 sec
D31	0.00001500 sec
D40	0.00439029 sec
L4	37
L5	53
P32	98.00 usec
TD0	15

======== CHANNEL f1 ========

SFO1	75.4828392 MHz
NUC1	13C
P1	15.00 usec
PLW1	22.00000000 W

======== CHANNEL f2 ========

SFO2	300.1612006 MHz
NUC2	1H
CPDPRG[2	waltz16
PCPD2	98.00 usec
PLW2	9.30000019 W
PLW12	0.29359001 W
PLW13	0.20359001 W

F2 - Processing parameters

SI	32768
SF	75.4753342 MHz
WDW	EM
SSB	0
LB	1.00 Hz
GB	0
PC	1.40

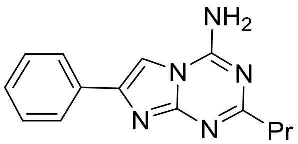
NH2
N
N
N
N
Pr

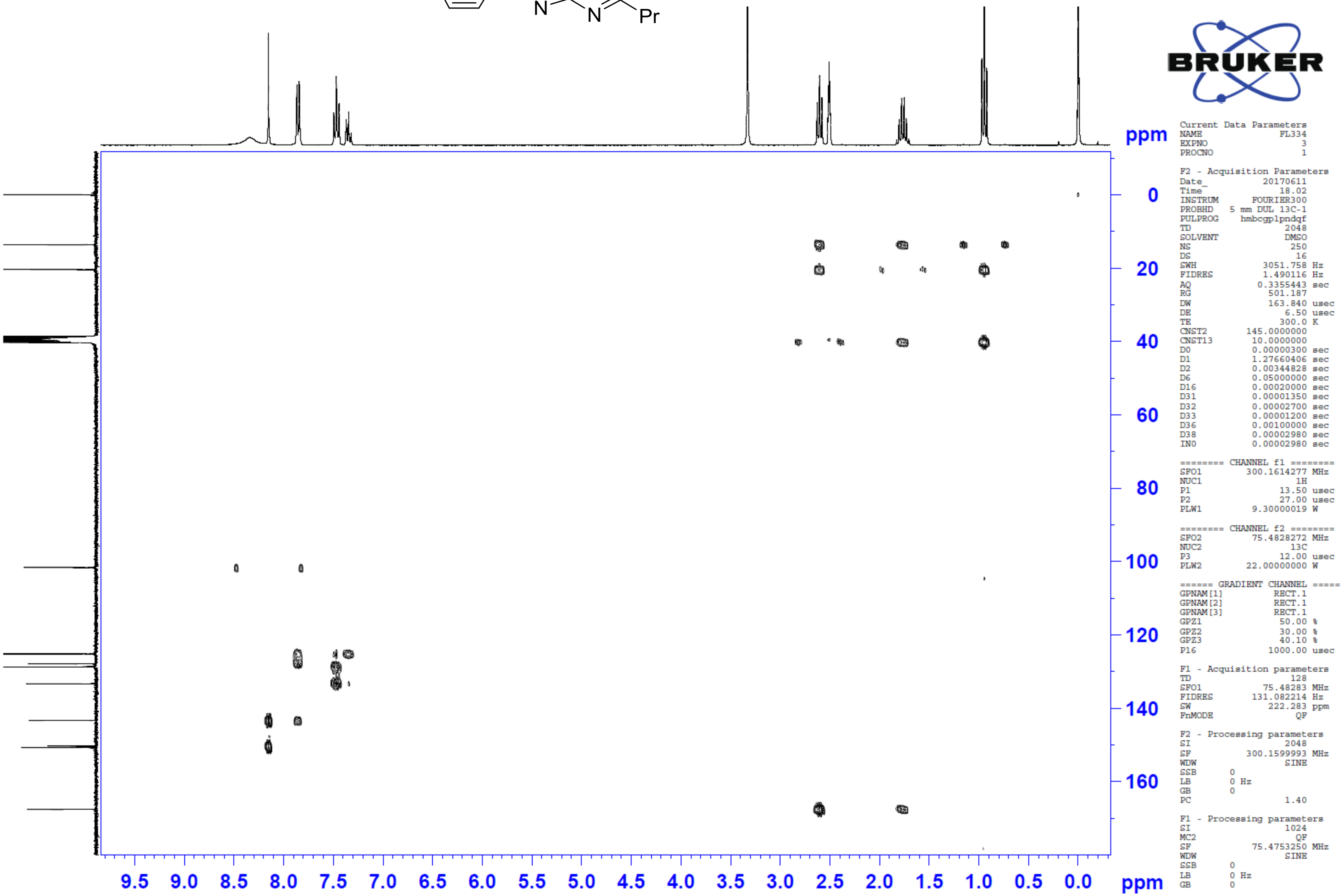
ppm
0
20
40
60
80
100
120
140
160
9.5
9.0
8.5
8.0
7.5
7.0
6.5
6.0
5.5
5.0
4.5
4.0
3.5
3.0
2.5
2.0
1.5
1.0
0.5
0.0
ppm
Current Data Parameters
NAME FL334
EXPNO 3
PROCNO 1
F2 - Acquisition Parameters
Date_ 20170611
Time 18.02
INSTRUM FOURIER300
PROBHD 5 mm DUL 13C-1
PULPROG hmbcgplpndqf
TD 2048
SOLVENT DMSO
NS 250
DS 16
SWH 3051.758 Hz
FIDRES 1.490116 Hz
AQ 0.3355443 sec
RG 501.187
DW 163.840 usec
DE 6.50 usec
TE 300.0 K
CNST2 145.0000000
CNST13 10.0000000
D0 0.00000300 sec
D1 1.27660406 sec
D2 0.00344828 sec
D6 0.05000000 sec
D16 0.00020000 sec
D31 0.00001350 sec
D32 0.00002700 sec
D33 0.00001200 sec
D36 0.00100000 sec
D38 0.00002980 sec
IN0 0.00002980 sec
======== CHANNEL f1 ========
SFO1 300.1614277 MHz
NUC1 1H
P1 13.50 usec
P2 27.00 usec
PLW1 9.30000019 W
======== CHANNEL f2 ========
SFO2 75.4828272 MHz
NUC2 13C
P3 12.00 usec
PLW2 22.00000000 W
====== GRADIENT CHANNEL =====
GPNAM[1] RECT.1
GPNAM[2] RECT.1
GPNAM[3] RECT.1
GPZ1 50.00 %
GPZ2 30.00 %
GPZ3 40.10 %
P16 1000.00 usec
F1 - Acquisition parameters
TD 128
SFO1 75.48283 MHz
FIDRES 131.082214 Hz
SW 222.283 ppm
FnMODE QF
F2 - Processing parameters
SI 2048
SF 300.1599993 MHz
WDW SINE
SSB 0
LB 0 Hz
GB 0
PC 1.40
F1 - Processing parameters
SI 1024
MC2 QF
SF 75.4753250 MHz
WDW SINE
SSB 0
LB 0 Hz
GB 0

4-Amino-2-propyl-7-(4-fluorophenyl)imidazo[1,2-a][1,3,5]triazine (5n)

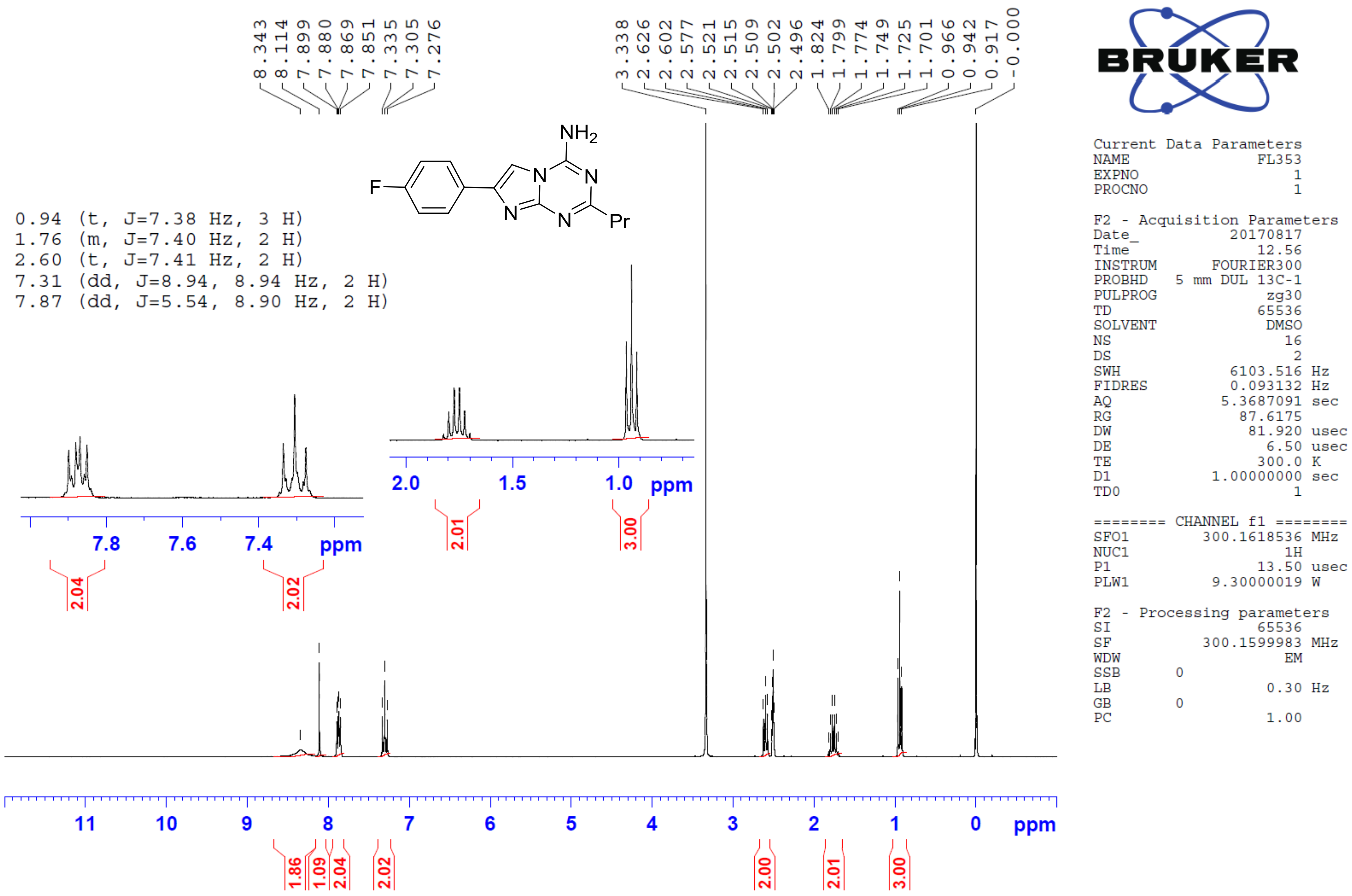

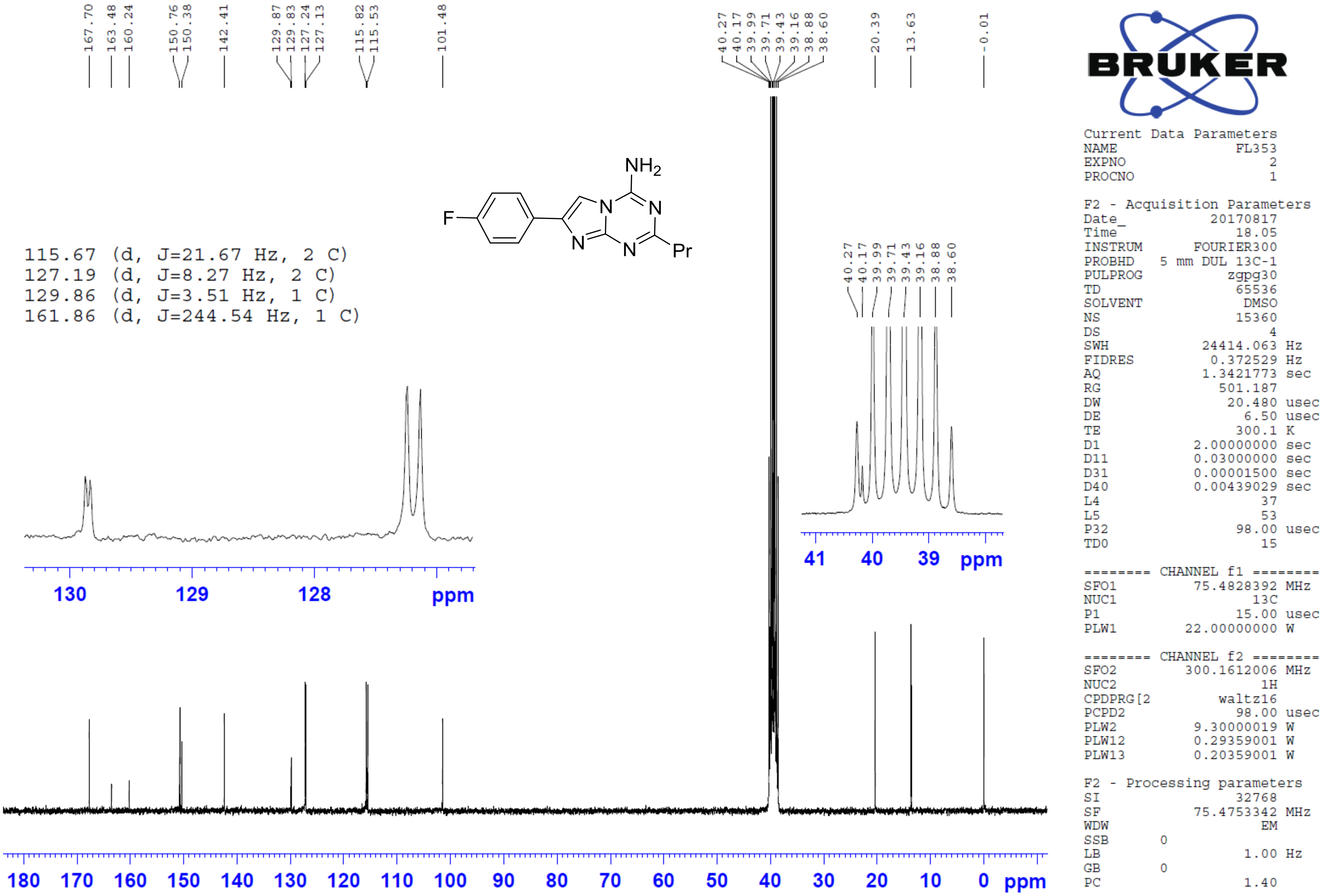
167.70
163.48
160.24
150.76
150.38
142.41
129.87
129.83
127.24
127.13
115.82
115.53
101.48
40.27
40.17
39.99
39.71
39.43
39.16
38.88
38.60
20.39
13.63
-0.01
NH2
F
N
N
N
N
Pr
115.67 (d, J=21.67 Hz, 2 C)
127.19 (d, J=8.27 Hz, 2 C)
129.86 (d, J=3.51 Hz, 1 C)
161.86 (d, J=244.54 Hz, 1 C)
130
129
128
ppm
41
40
39
ppm
180 170 160 150 140 130 120 110 100 90 80 70 60 50 40 30 20 10 0 ppm
BRUKER
Current Data Parameters
NAME FL353
EXPNO 2
PROCNO 1
F2 - Acquisition Parameters
Date_ 20170817
Time 18.05
INSTRUM FOURIER300
PROBHD 5 mm DUL 13C-1
PULPROG zgpg30
TD 65536
SOLVENT DMSO
NS 15360
DS 4
SWH 24414.063 Hz
FIDRES 0.372529 Hz
AQ 1.3421773 sec
RG 501.187
DW 20.480 usec
DE 6.50 usec
TE 300.1 K
D1 2.00000000 sec
D11 0.03000000 sec
D31 0.00001500 sec
D40 0.00439029 sec
L4 37
L5 53
P32 98.00 usec
TD0 15
======== CHANNEL f1 ========
SFO1 75.4828392 MHz
NUC1 13C
P1 15.00 usec
PLW1 22.00000000 W
======== CHANNEL f2 ========
SFO2 300.1612006 MHz
NUC2 1H
CPDPRG[2 waltz16
PCPD2 98.00 usec
PLW2 9.30000019 W
PLW12 0.29359001 W
PLW13 0.20359001 W
F2 - Processing parameters
SI 32768
SF 75.4753342 MHz
WDW EM
SSB 0
LB 1.00 Hz
GB 0
PC 1.40

4-Amino-2-propyl-7-(4-chlorophenyl)imidazo[1,2-a][1,3,5]triazine (5o)

0.94 (t, J=7.38 Hz, 3 H)
1.76 (m, J=7.40 Hz, 2 H)
2.60 (t, J=7.40 Hz, 2 H)
7.53 (d, J=8.70 Hz, 2 H)
7.85 (d, J=8.90 Hz, 2 H)

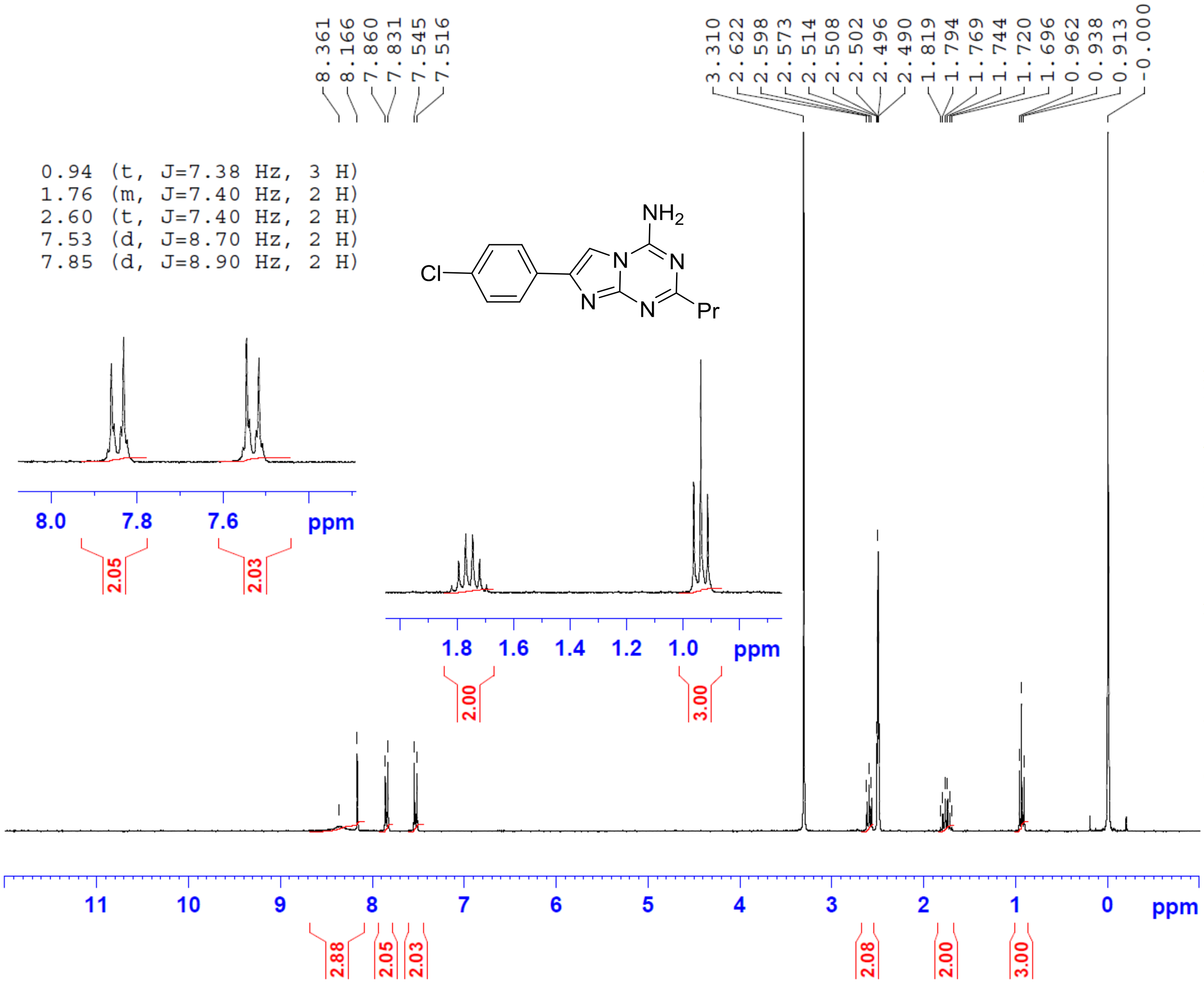

Current Data Parameters	
NAME	FL363
EXPNO	1
PROCNO	1

F2 - Acquisition Parameters	
Date_	20170918
Time	12.33
INSTRUM	FOURIER300
PROBHD	5 mm DUL 13C-1
PULPROG	zg30
TD	65536
SOLVENT	DMSO
NS	16
DS	2
SWH	6103.516 Hz
FIDRES	0.093132 Hz
AQ	5.3687091 sec
RG	145.81
DW	81.920 usec
DE	6.50 usec
TE	300.0 K
D1	1.00000000 sec
TD0	1

======== CHANNEL f1 ========	
SFO1	300.1618536 MHz
NUC1	1H
P1	13.50 usec
PLW1	9.30000019 W

F2 - Processing parameters		
SI		65536
SF		300.1600004 MHz
WDW		EM
SSB	0	
LB		0.30 Hz
GB	0	
PC		1.00

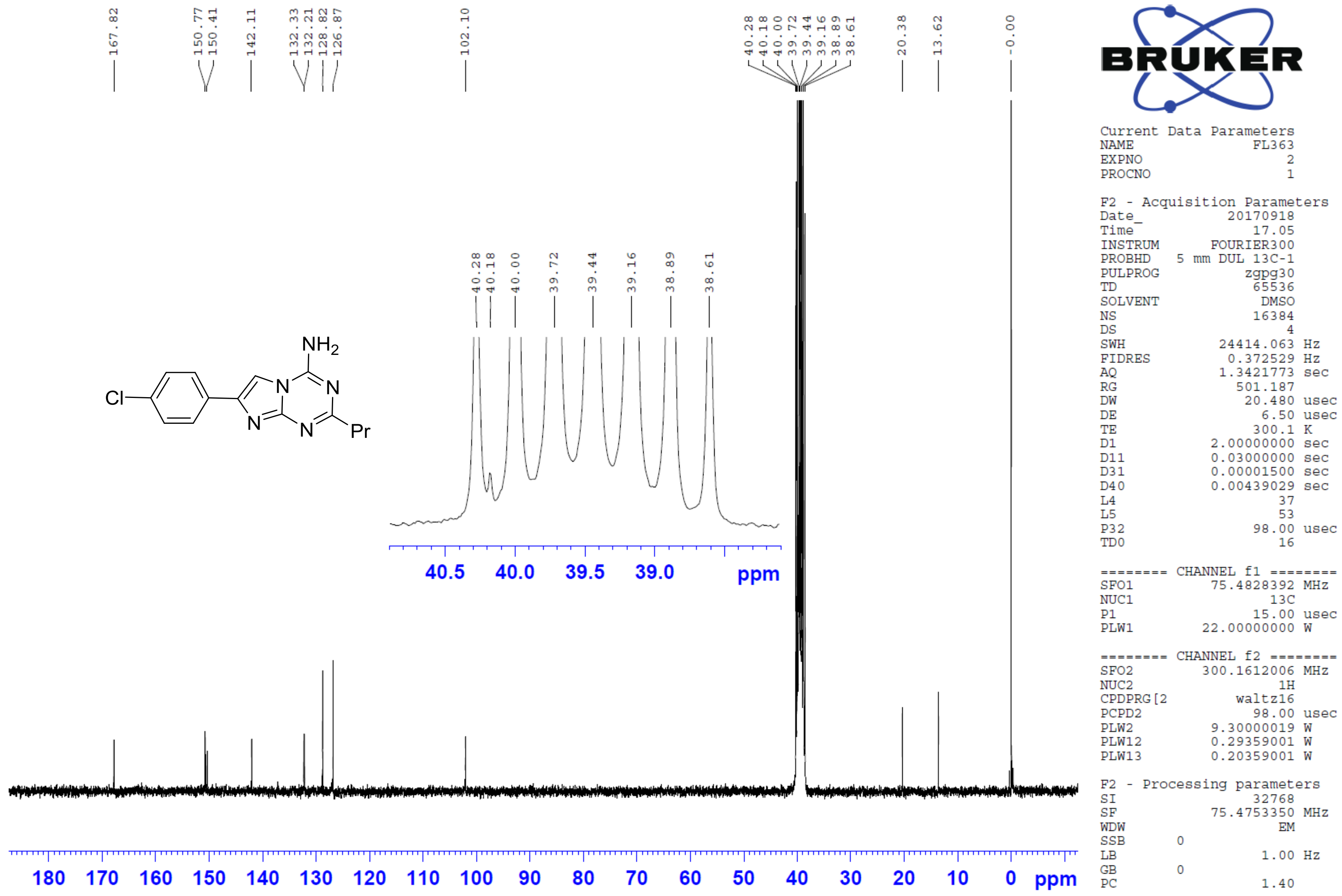

167.82
150.77
150.41
142.11
132.33
132.21
128.82
126.87
102.10
40.28
40.18
40.00
39.72
39.44
39.16
38.89
38.61
20.38
13.62
-0.00
NH2
Cl
N
N
N
N
Pr
40.5
40.0
39.5
39.0
ppm
180
170
160
150
140
130
120
110
100
90
80
70
60
50
40
30
20
10
0
ppm
BRUKER
Current Data Parameters
NAME FL363
EXPNO 2
PROCNO 1
F2 - Acquisition Parameters
Date_ 20170918
Time 17.05
INSTRUM FOURIER300
PROBHD 5 mm DUL 13C-1
PULPROG zgpg30
TD 65536
SOLVENT DMSO
NS 16384
DS 4
SWH 24414.063 Hz
FIDRES 0.372529 Hz
AQ 1.3421773 sec
RG 501.187
DW 20.480 usec
DE 6.50 usec
TE 300.1 K
D1 2.00000000 sec
D11 0.03000000 sec
D31 0.00001500 sec
D40 0.00439029 sec
L4 37
L5 53
P32 98.00 usec
TD0 16
======== CHANNEL f1 ========
SFO1 75.4828392 MHz
NUC1 13C
P1 15.00 usec
PLW1 22.00000000 W
======== CHANNEL f2 ========
SFO2 300.1612006 MHz
NUC2 1H
CPDPRG[2 waltz16
PCPD2 98.00 usec
PLW2 9.30000019 W
PLW12 0.29359001 W
PLW13 0.20359001 W
F2 - Processing parameters
SI 32768
SF 75.4753350 MHz
WDW EM
SSB 0
LB 1.00 Hz
GB 0
PC 1.40

4-Amino-2-propyl-7-(4-methylphenyl)imidazo[1,2-a][1,3,5]triazine (5p)

0.94 (t, J=7.40 Hz, 3 H)
1.77 (m, J=7.40 Hz, 2 H)
2.60 (t, J=7.40 Hz, 2 H)
7.27 (d, J=7.89 Hz, 2 H)
7.75 (d, J=8.07 Hz, 2 H)

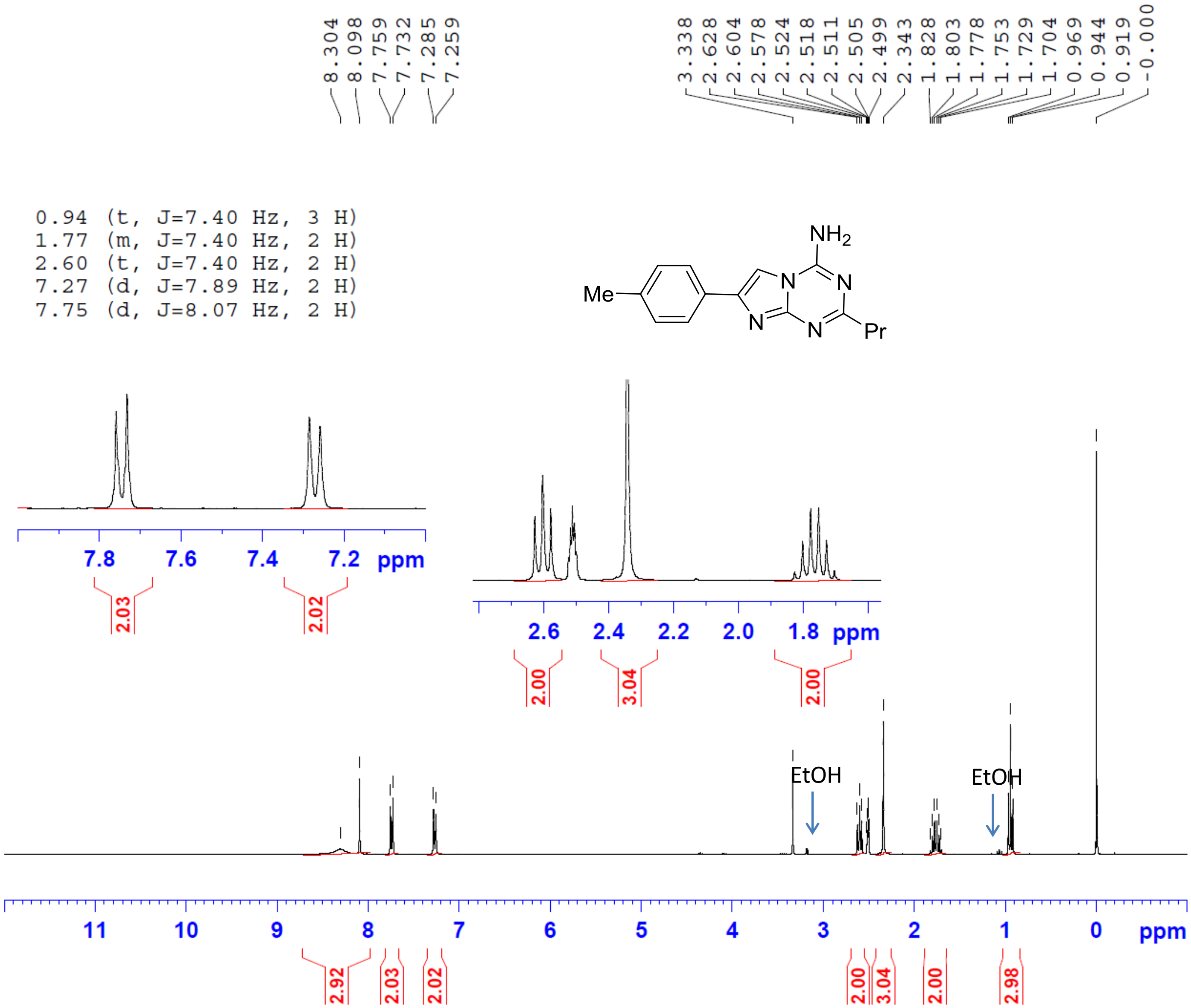

Current Data Parameters
NAME FL367
EXPNO 1
PROCNO 1

F2 - Acquisition Parameters
Date_ 20170924
Time 15.53
INSTRUM FOURIER300
PROBHD 5 mm DUL 13C-1
PULPROG zg30
TD 65536
SOLVENT DMSO
NS 16
DS 2
SWH 6103.516 Hz
FIDRES 0.093132 Hz
AQ 5.3687091 sec
RG 59.2172
DW 81.920 usec
DE 6.50 usec
TE 300.0 K
D1 1.00000000 sec
TD0 1

======== CHANNEL f1 ========
SFO1 300.1618536 MHz
NUC1 1H
P1 13.50 usec
PLW1 9.30000019 W

F2 - Processing parameters
SI 65536
SF 300.1599975 MHz
WDW EM
SSB 0
LB 0.30 Hz
GB 0
PC 1.00

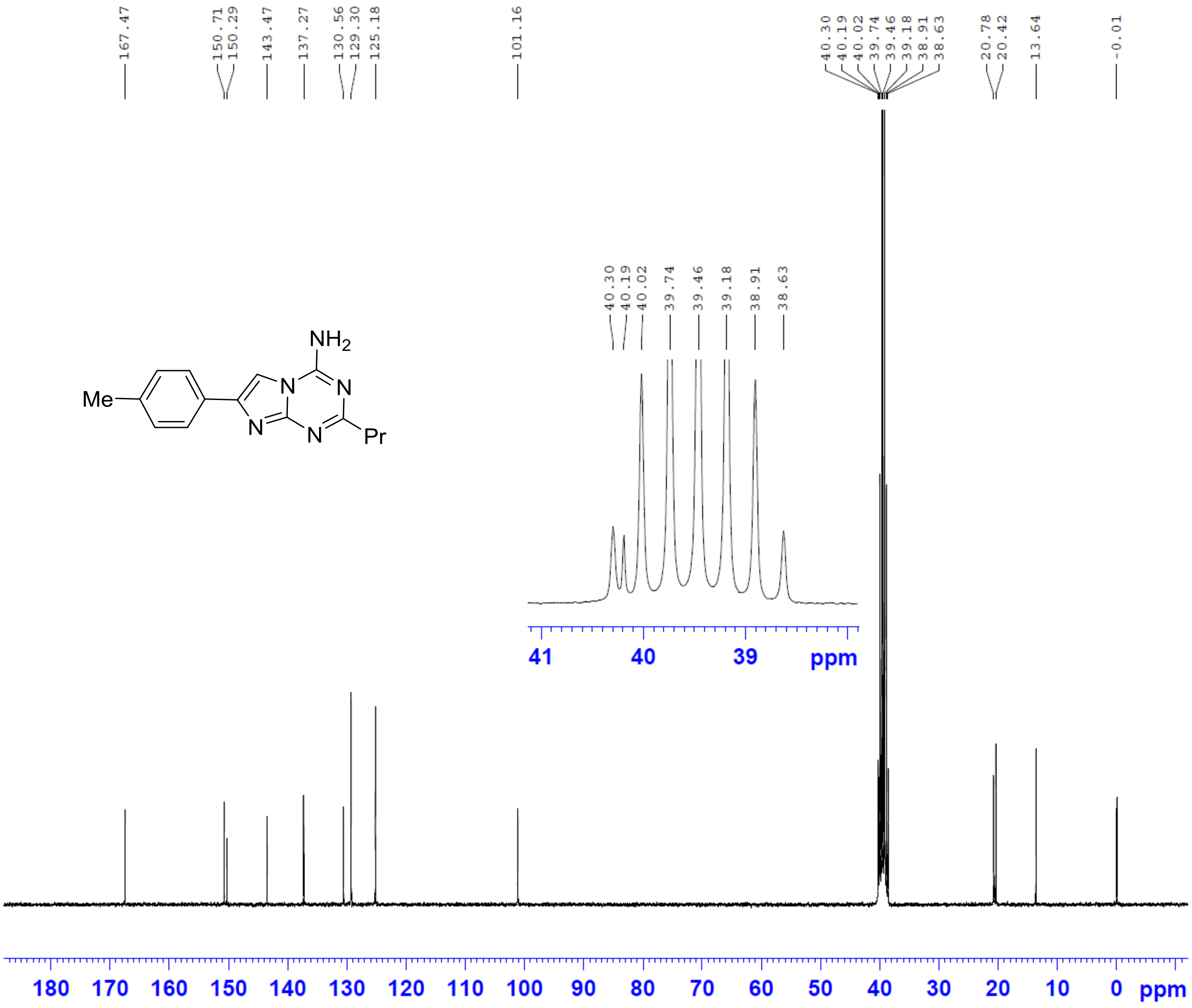

Current Data Parameters
NAME FL367
EXPNO 2
PROCNO 1

F2 - Acquisition Parameters
Date_ 20170925
Time 0.55
INSTRUM FOURIER300
PROBHD 5 mm DUL 13C-1
PULPROG zgpg30
TD 65536
SOLVENT DMSO
NS 9216
DS 4
SWH 24414.063 Hz
FIDRES 0.372529 Hz
AQ 1.3421773 sec
RG 501.187
DW 20.480 usec
DE 6.50 usec
TE 300.1 K
D1 2.00000000 sec
D11 0.03000000 sec
D31 0.00001500 sec
D40 0.00439029 sec
L4 37
L5 53
P32 98.00 usec
TD0 9

======== CHANNEL f1 ========
SFO1 75.4828392 MHz
NUC1 13C
P1 15.00 usec
PLW1 22.00000000 W

======== CHANNEL f2 ========
SFO2 300.1612006 MHz
NUC2 1H
CPDPRG[2 waltz16
PCPD2 98.00 usec
PLW2 9.30000019 W
PLW12 0.29359001 W
PLW13 0.20359001 W

F2 - Processing parameters
SI 32768
SF 75.4753331 MHz
WDW EM
SSB 0
LB 1.00 Hz
GB 0
PC 1.40

4-Amino-2-propyl-7-(4-methoxyphenyl)imidazo[1,2-a][1,3,5]triazine (5q)

0.94 (t, J=7.38 Hz, 3 H)
1.76 (m, J=7.39 Hz, 2 H)
2.59 (t, J=7.41 Hz, 2 H)
7.04 (d, J=8.88 Hz, 2 H)
7.78 (d, J=8.85 Hz, 2 H)

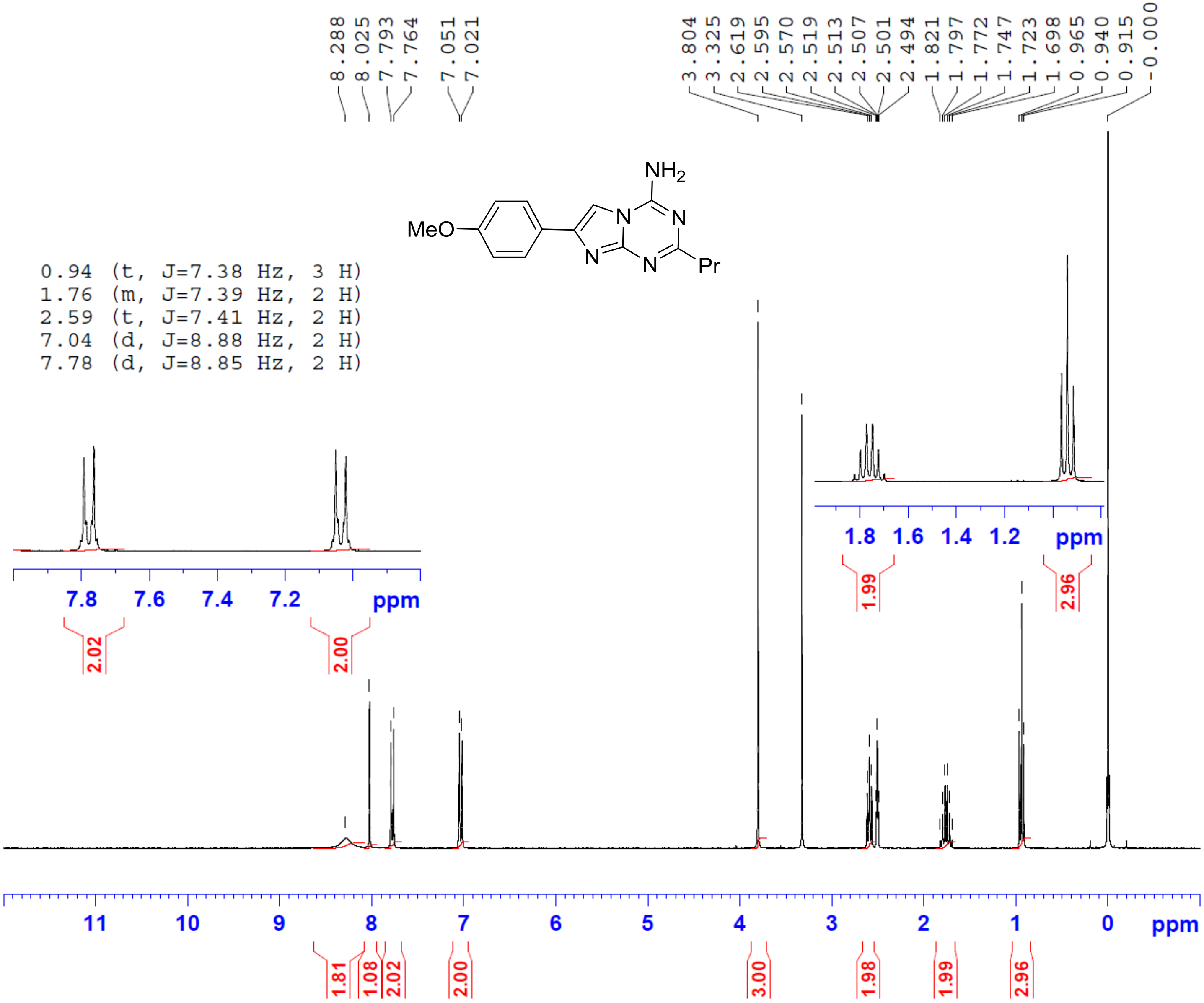

BRUKER

Current Data Parameters
NAME FL348
EXPNO 1
PROCNO 1

F2 - Acquisition Parameters
Date_ 20170808
Time 14.04
INSTRUM FOURIER300
PROBHD 5 mm DUL 13C-1
PULPROG zg30
TD 65536
SOLVENT DMSO
NS 16
DS 2
SWH 6103.516 Hz
FIDRES 0.093132 Hz
AQ 5.3687091 sec
RG 87.3614
DW 81.920 usec
DE 6.50 usec
TE 300.2 K
D1 1.00000000 sec
TD0 1

======== CHANNEL f1 ========
SFO1 300.1618536 MHz
NUC1 1H
P1 13.50 usec
PLW1 9.30000019 W

F2 - Processing parameters
SI 65536
SF 300.1599988 MHz
WDW EM
SSB 0
LB 0.30 Hz
GB 0
PC 1.00

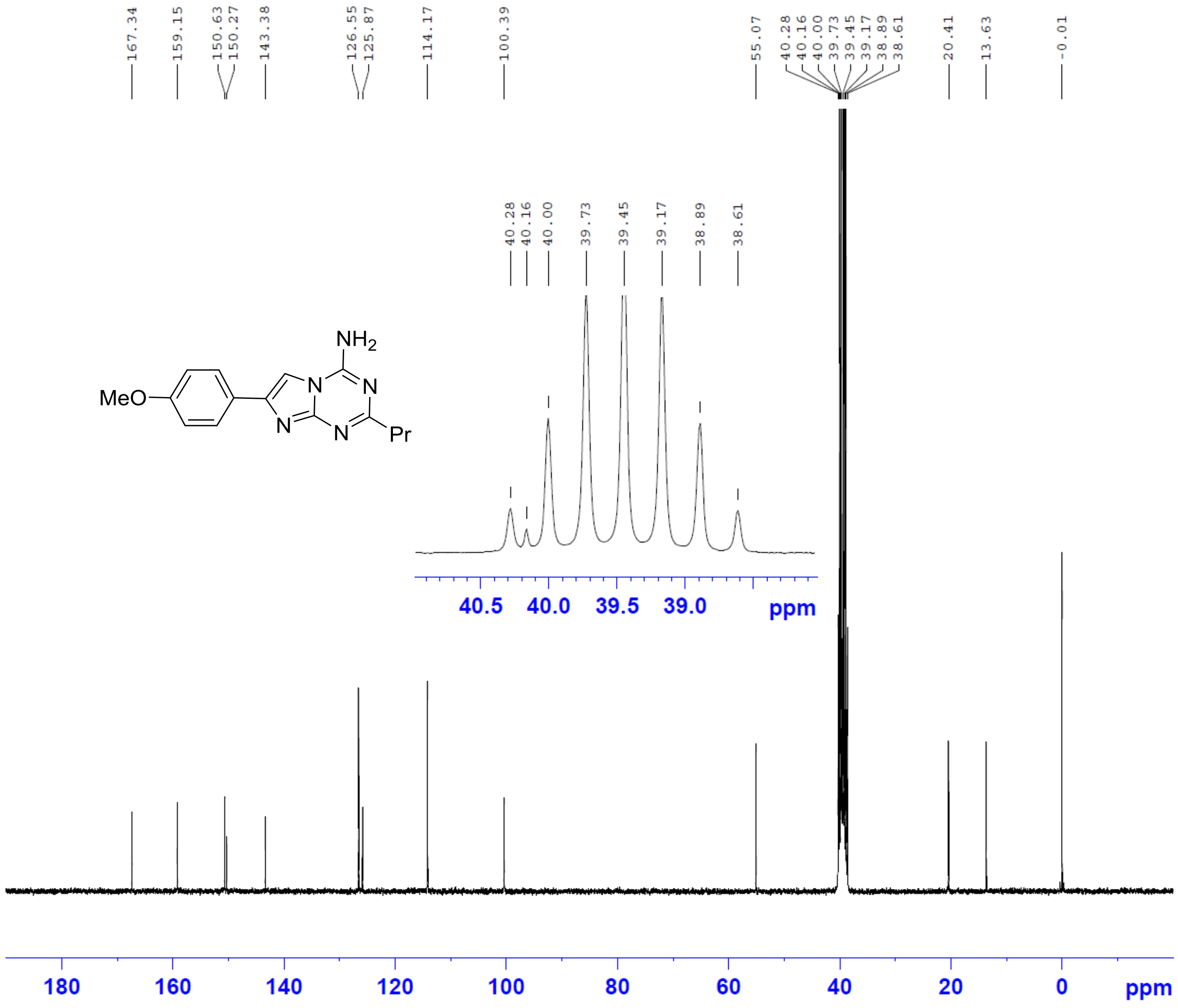

Current Data Parameters
NAME FL348
EXPNO 2
PROCNO 1

F2 - Acquisition Parameters
Date_ 20170808
Time 18.05
INSTRUM FOURIER300
PROBHD 5 mm DUL 13C-1
PULPROG zgpg30
TD 65536
SOLVENT DMSO
NS 14336
DS 4
SWH 24414.063 Hz
FIDRES 0.372529 Hz
AQ 1.3421773 sec
RG 501.187
DW 20.480 usec
DE 6.50 usec
TE 300.2 K
D1 2.00000000 sec
D11 0.03000000 sec
D31 0.00001500 sec
D40 0.00439029 sec
L4 37
L5 53
P32 98.00 usec
TD0 14

======== CHANNEL f1 ========
SFO1 75.4828392 MHz
NUC1 13C
P1 15.00 usec
PLW1 22.00000000 W

======== CHANNEL f2 ========
SFO2 300.1612006 MHz
NUC2 1H
CPDPRG[2 waltz16
PCPD2 98.00 usec
PLW2 9.30000019 W
PLW12 0.29359001 W
PLW13 0.20359001 W

F2 - Processing parameters
SI 32768
SF 75.4753342 MHz
WDW EM
SSB 0
LB 1.00 Hz
GB 0
PC 1.40

4-Amino-2-propyl-7-(4-biphenyl)imidazo[1,2-a][1,3,5]triazine (5r)

0.95 (t, J=7.37 Hz, 3 H)
1.78 (m, J=7.39 Hz, 2 H)
2.62 (t, J=7.40 Hz, 2 H)
7.38 (t, J=7.28 Hz, 1 H)
7.49 (t, J=7.52 Hz, 2 H)
7.73 (d, J=7.65 Hz, 2 H)
7.79 (d, J=8.37 Hz, 2 H)
7.95 (d, J=8.28 Hz, 2 H)

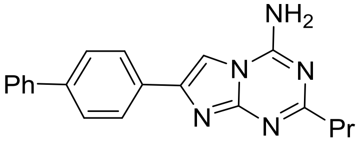

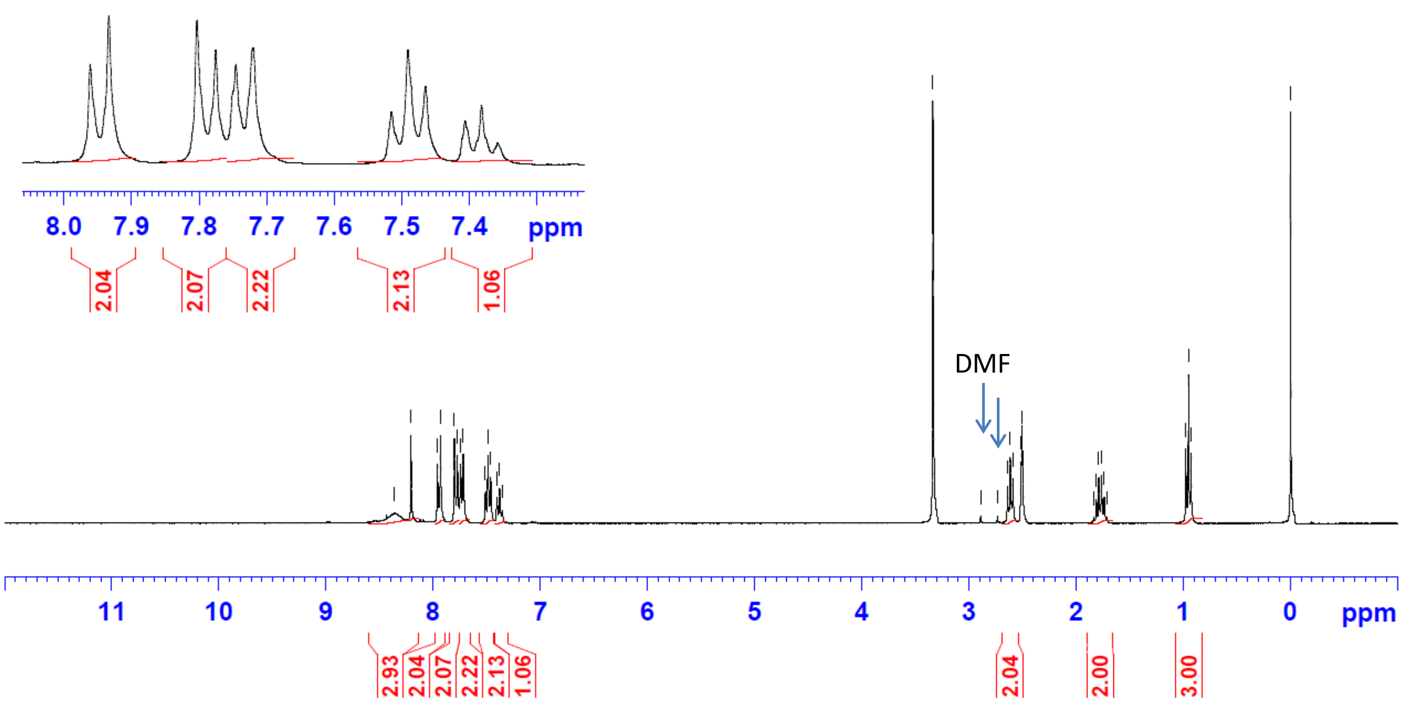


```
Current Data Parameters
NAME                 FL374
EXPNO                    4
PROCNO                   1

F2 - Acquisition Parameters
Date_             20171023
Time                 17.01
INSTRUM         FOURIER300
PROBHD   5 mm DUL 13C-1
PULPROG               zg30
TD                   65536
SOLVENT               DMSO
NS                      16
DS                       2
SWH            6103.516 Hz
FIDRES         0.093132 Hz
AQ            5.3687091 sec
RG                86.1986
DW               81.920 usec
DE                 6.50 usec
TE                300.0 K
D1           1.00000000 sec
TD0                      1

======== CHANNEL f1 ========
SFO1        300.1618536 MHz
NUC1                    1H
P1                 13.50 usec
PLW1          9.30000019 W

F2 - Processing parameters
SI                   65536
SF          300.1599986 MHz
WDW                     EM
SSB      0
LB                  0.30 Hz
GB       0
PC                    1.00
```

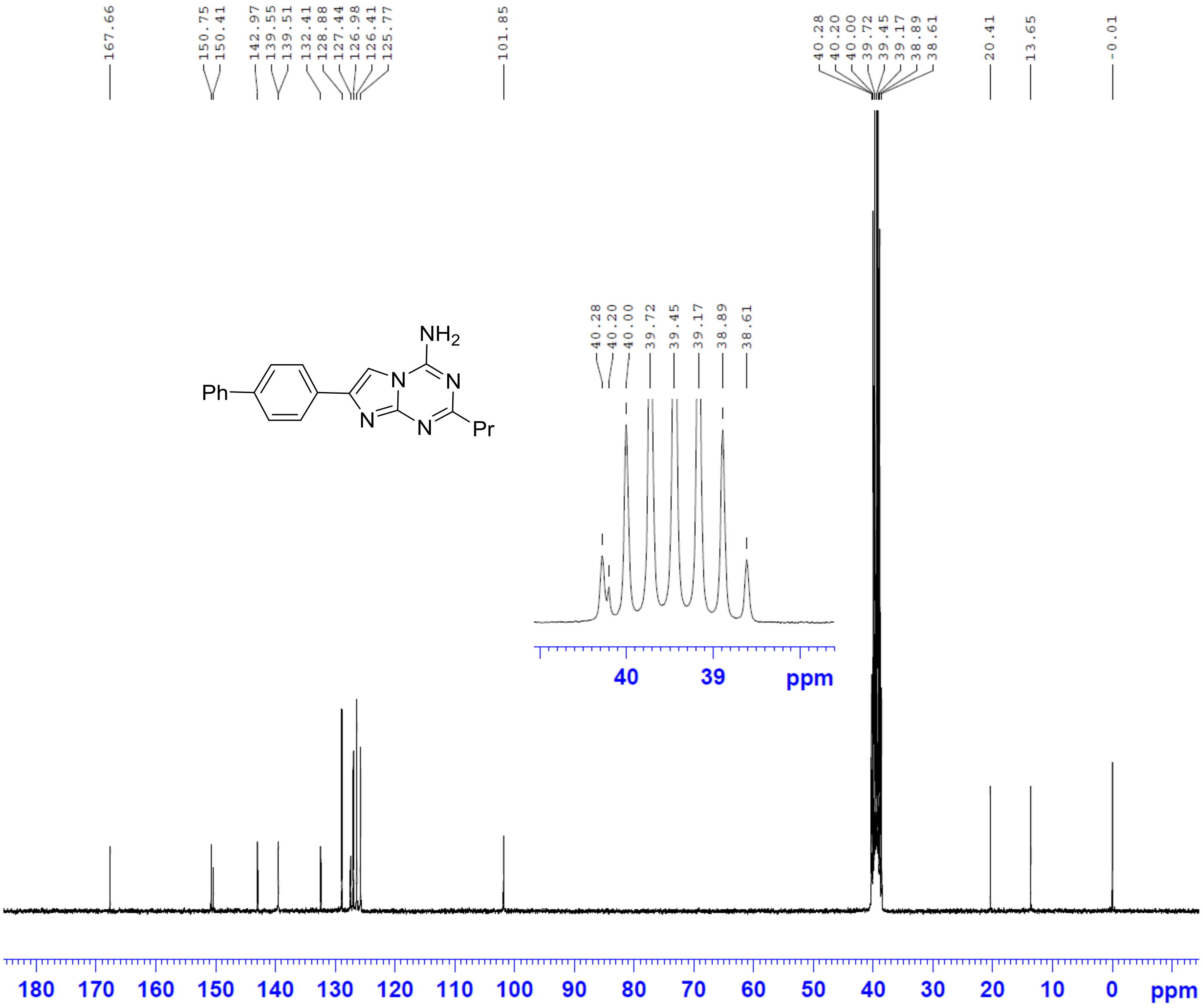


```
Current Data Parameters
NAME              FL374
EXPNO                 3
PROCNO                1

F2 - Acquisition Parameters
Date_          20171021
Time              18.05
INSTRUM      FOURIER300
PROBHD   5 mm DUL 13C-1
PULPROG          zgpg30
TD                65536
SOLVENT            DMSO
NS                17408
DS                    4
SWH           24414.063 Hz
FIDRES         0.372529 Hz
AQ            1.3421773 sec
RG              501.187
DW               20.480 usec
DE                 6.50 usec
TE                300.0 K
D1           2.00000000 sec
D11          0.03000000 sec
D31          0.00001500 sec
D40          0.00439029 sec
L4                   37
L5                   53
P32               98.00 usec
TD0                  17

======== CHANNEL f1 ========
SFO1         75.4828392 MHz
NUC1                13C
P1                15.00 usec
PLW1        22.00000000 W

======== CHANNEL f2 ========
SFO2        300.1612006 MHz
NUC2                 1H
CPDPRG[2        waltz16
PCPD2             98.00 usec
PLW2         9.30000019 W
PLW12        0.29359001 W
PLW13        0.20359001 W

F2 - Processing parameters
SI                32768
SF           75.4753342 MHz
WDW                  EM
SSB      0
LB                 1.00 Hz
GB       0
PC                 1.40
```

4-amino-2-butyl-7-phenylimidazo[1,2-a][1,3,5]triazine (5s)

0.92 (t, J=7.32 Hz, 3 H)
1.36 (m, J=7.39 Hz, 2 H)
1.72 (m, J=7.50 Hz, 2 H)
2.62 (t, J=7.52 Hz, 2 H)
7.35 (t, J=7.34 Hz, 1 H)
7.47 (t, J=7.50 Hz, 2 H)
7.85 (d, J=7.02 Hz, 2 H)

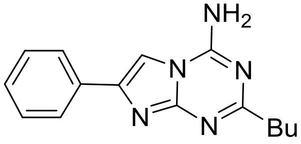

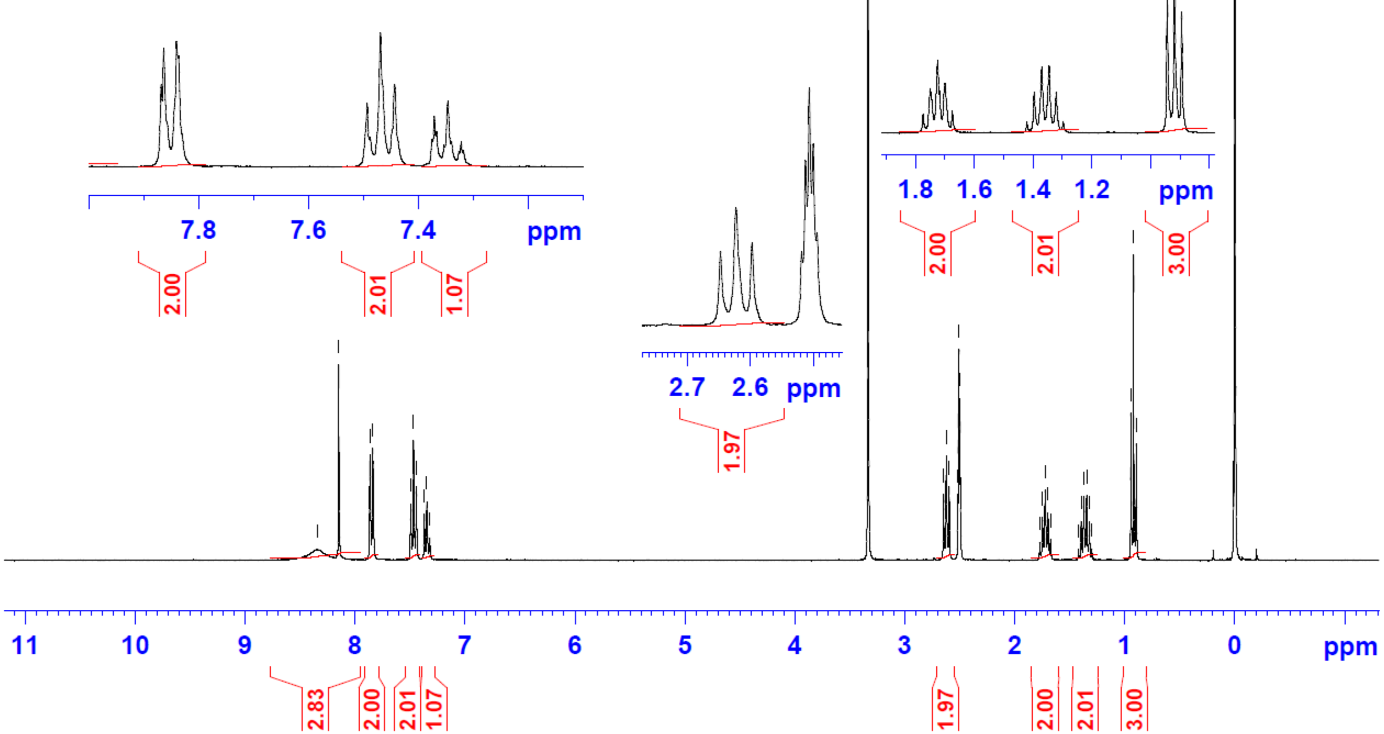

Current Data Parameters
NAME FL335
EXPNO 1
PROCNO 1

F2 - Acquisition Parameters
Date_ 20170601
Time 14.14
INSTRUM FOURIER300
PROBHD 5 mm DUL 13C-1
PULPROG zg30
TD 65536
SOLVENT DMSO
NS 16
DS 2
SWH 6103.516 Hz
FIDRES 0.093132 Hz
AQ 5.3687091 sec
RG 107.76
DW 81.920 usec
DE 6.50 usec
TE 297.7 K
D1 1.00000000 sec
TD0 1

======== CHANNEL f1 ========
SFO1 300.1618536 MHz
NUC1 1H
P1 13.50 usec
PLW1 9.30000019 W

F2 - Processing parameters
SI 65536
SF 300.1599990 MHz
WDW EM
SSB 0
LB 0.30 Hz
GB 0
PC 1.00

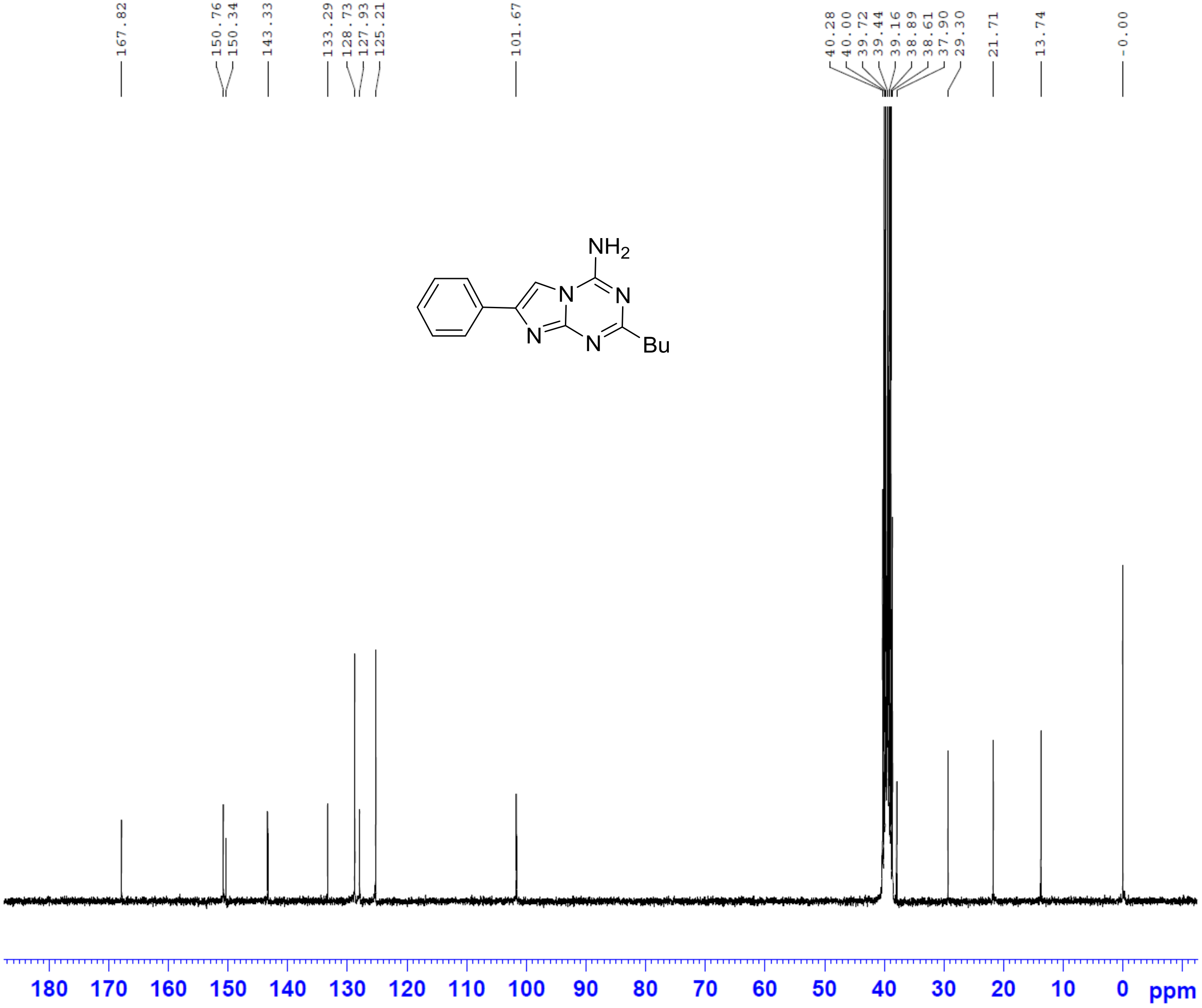

Current Data Parameters
NAME FL335
EXPNO 2
PROCNO 1

F2 - Acquisition Parameters
Date_ 20170608
Time 18.10
INSTRUM FOURIER300
PROBHD 5 mm DUL 13C-1
PULPROG zgpg30
TD 65536
SOLVENT DMSO
NS 15360
DS 4
SWH 24414.063 Hz
FIDRES 0.372529 Hz
AQ 1.3421773 sec
RG 501.187
DW 20.480 usec
DE 6.50 usec
TE 300.1 K
D1 2.00000000 sec
D11 0.03000000 sec
D31 0.00001500 sec
D40 0.00439029 sec
L4 37
L5 53
P32 98.00 usec
TD0 15

======== CHANNEL f1 ========
SFO1 75.4828392 MHz
NUC1 13C
P1 15.00 usec
PLW1 22.00000000 W

======== CHANNEL f2 ========
SFO2 300.1612006 MHz
NUC2 1H
CPDPRG[2 waltz16
PCPD2 98.00 usec
PLW2 9.30000019 W
PLW12 0.29359001 W
PLW13 0.20359001 W

F2 - Processing parameters
SI 32768
SF 75.4753343 MHz
WDW EM
SSB 0
LB 1.00 Hz
GB 0
PC 1.40

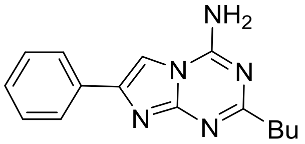

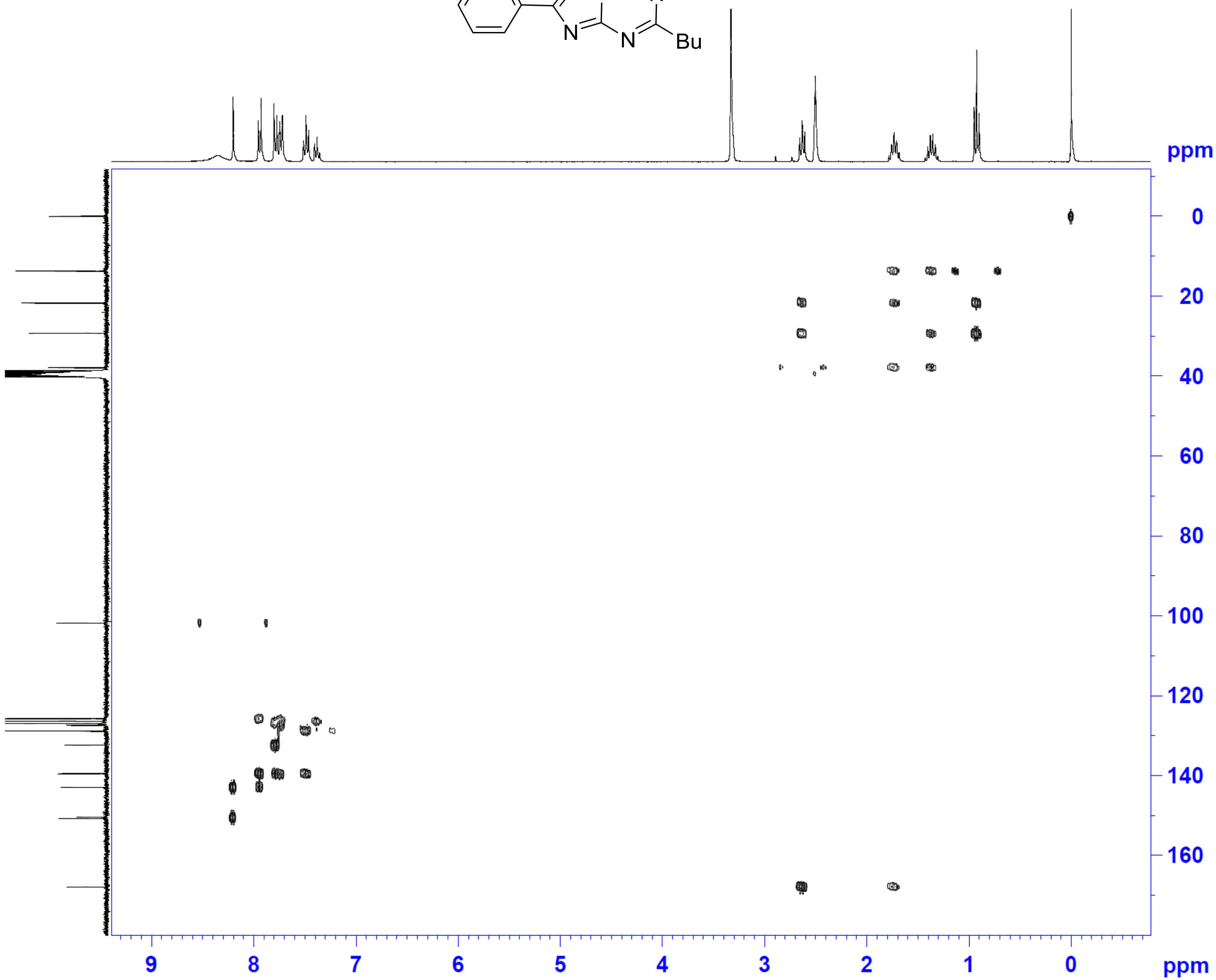


```
Current Data Parameters
NAME              FL375
EXPNO                 6
PROCNO                1

F2 - Acquisition Parameters
Date_          20171123
Time              18.03
INSTRUM      FOURIER300
PROBHD   5 mm DUL 13C-1
PULPROG    hmbcgplpndqf
TD                 2048
SOLVENT            DMSO
NS                  250
DS                   16
SWH            3051.758 Hz
FIDRES         1.490116 Hz
AQ            0.3355443 sec
RG              501.187
DW              163.840 usec
DE                 6.50 usec
TE                300.0 K
CNST2       145.0000000
CNST13       10.0000000
D0           0.00000300 sec
D1           1.32780397 sec
D2           0.00344828 sec
D6           0.05000000 sec
D16          0.00020000 sec
D31          0.00001350 sec
D32          0.00002700 sec
D33          0.00001200 sec
D36          0.00100000 sec
D38          0.00002980 sec
IN0          0.00002980 sec

======== CHANNEL f1 ========
SFO1        300.1612919 MHz
NUC1                 1H
P1                13.50 usec
P2                27.00 usec
PLW1         9.30000019 W

======== CHANNEL f2 ========
SFO2         75.4828272 MHz
NUC2                13C
P3                12.00 usec
PLW2        22.00000000 W

====== GRADIENT CHANNEL =====
GPNAM[1]         RECT.1
GPNAM[2]         RECT.1
GPNAM[3]         RECT.1
GPZ1              50.00 %
GPZ2              30.00 %
GPZ3              40.10 %
P16             1000.00 usec

F1 - Acquisition parameters
TD                  128
SFO1           75.48283 MHz
FIDRES       131.082214 Hz
SW              222.283 ppm
FnMODE               QF

F2 - Processing parameters
SI                 2048
SF          300.1599992 MHz
WDW                SINE
SSB      0
LB       0 Hz
GB       0
PC                 1.40

F1 - Processing parameters
SI                 1024
MC2                  QF
SF           75.4753292 MHz
WDW                SINE
SSB      0
LB       0 Hz
GB       0
```

4-Amino-2-butyl-7-(4-fluorophenyl)imidazo[1,2-a][1,3,5]triazine (5t)

0.92 (t, J=7.34 Hz, 3 H)
1.36 (m, J=7.38 Hz, 2 H)
1.72 (m, J=7.49 Hz, 2 H)
2.62 (t, J=7.52 Hz, 2 H)
7.31 (dd, J=8.93, 8.93 Hz, 2 H)
7.88 (dd, J=5.55, 8.88 Hz, 2 H)

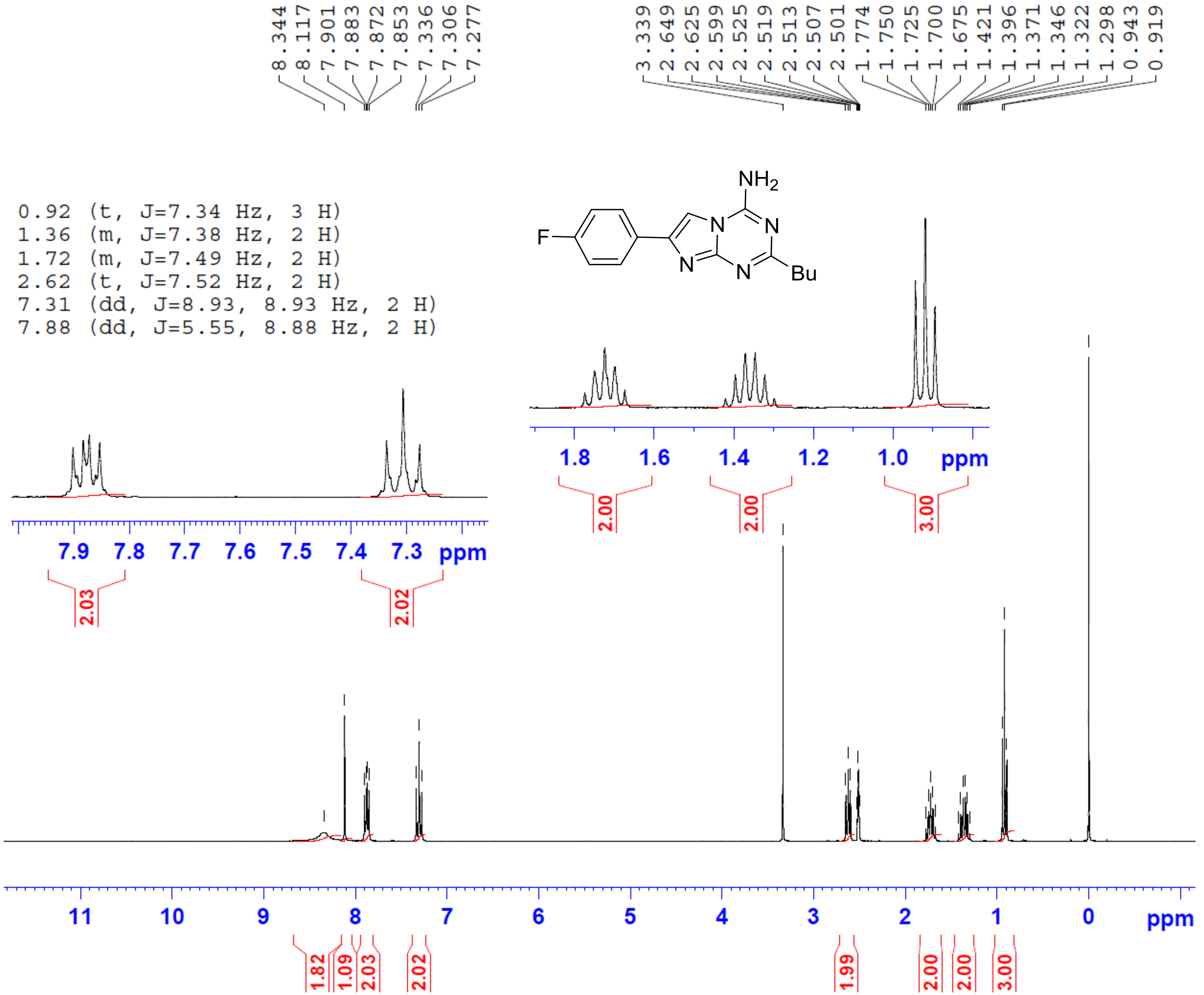

BRUKER

Current Data Parameters
NAME FL354
EXPNO 10
PROCNO 1

F2 - Acquisition Parameters
Date_ 20170817
Time 13.00
INSTRUM FOURIER300
PROBHD 5 mm DUL 13C-1
PULPROG zg30
TD 65536
SOLVENT DMSO
NS 16
DS 2
SWH 6103.516 Hz
FIDRES 0.093132 Hz
AQ 5.3687091 sec
RG 71.026
DW 81.920 usec
DE 6.50 usec
TE 300.0 K
D1 1.00000000 sec
TD0 1

======== CHANNEL f1 ========
SFO1 300.1618536 MHz
NUC1 1H
P1 13.50 usec
PLW1 9.30000019 W

F2 - Processing parameters
SI 65536
SF 300.1599969 MHz
WDW EM
SSB 0
LB 0.30 Hz
GB 0
PC 1.00

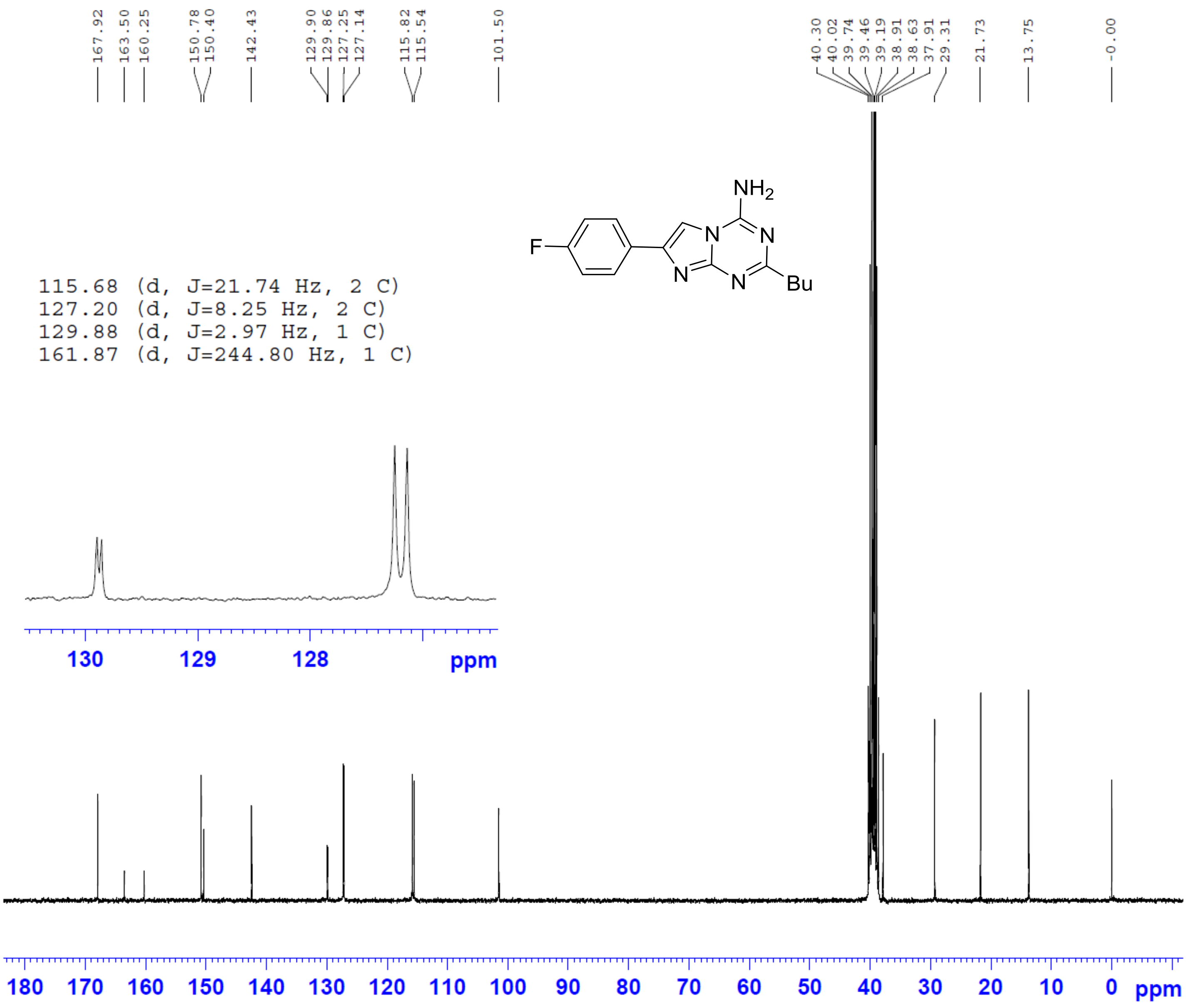

Current Data Parameters
NAME FL354
EXPNO 11
PROCNO 1

F2 - Acquisition Parameters
Date_ 20170818
Time 18.05
INSTRUM FOURIER300
PROBHD 5 mm DUL 13C-1
PULPROG zgpg30
TD 65536
SOLVENT DMSO
NS 15360
DS 4
SWH 24414.063 Hz
FIDRES 0.372529 Hz
AQ 1.3421773 sec
RG 501.187
DW 20.480 usec
DE 6.50 usec
TE 300.0 K
D1 2.00000000 sec
D11 0.03000000 sec
D31 0.00001500 sec
D40 0.00439029 sec
L4 37
L5 53
P32 98.00 usec
TD0 15

======== CHANNEL f1 ========
SFO1 75.4828392 MHz
NUC1 13C
P1 15.00 usec
PLW1 22.00000000 W

======== CHANNEL f2 ========
SFO2 300.1612006 MHz
NUC2 1H
CPDPRG[2 waltz16
PCPD2 98.00 usec
PLW2 9.30000019 W
PLW12 0.29359001 W
PLW13 0.20359001 W

F2 - Processing parameters
SI 32768
SF 75.4753327 MHz
WDW EM
SSB 0
LB 1.00 Hz
GB 0
PC 1.40

4-Amino-2-butyl-7-(4-chlorophenyl)imidazo[1,2-a][1,3,5]triazine (5u)

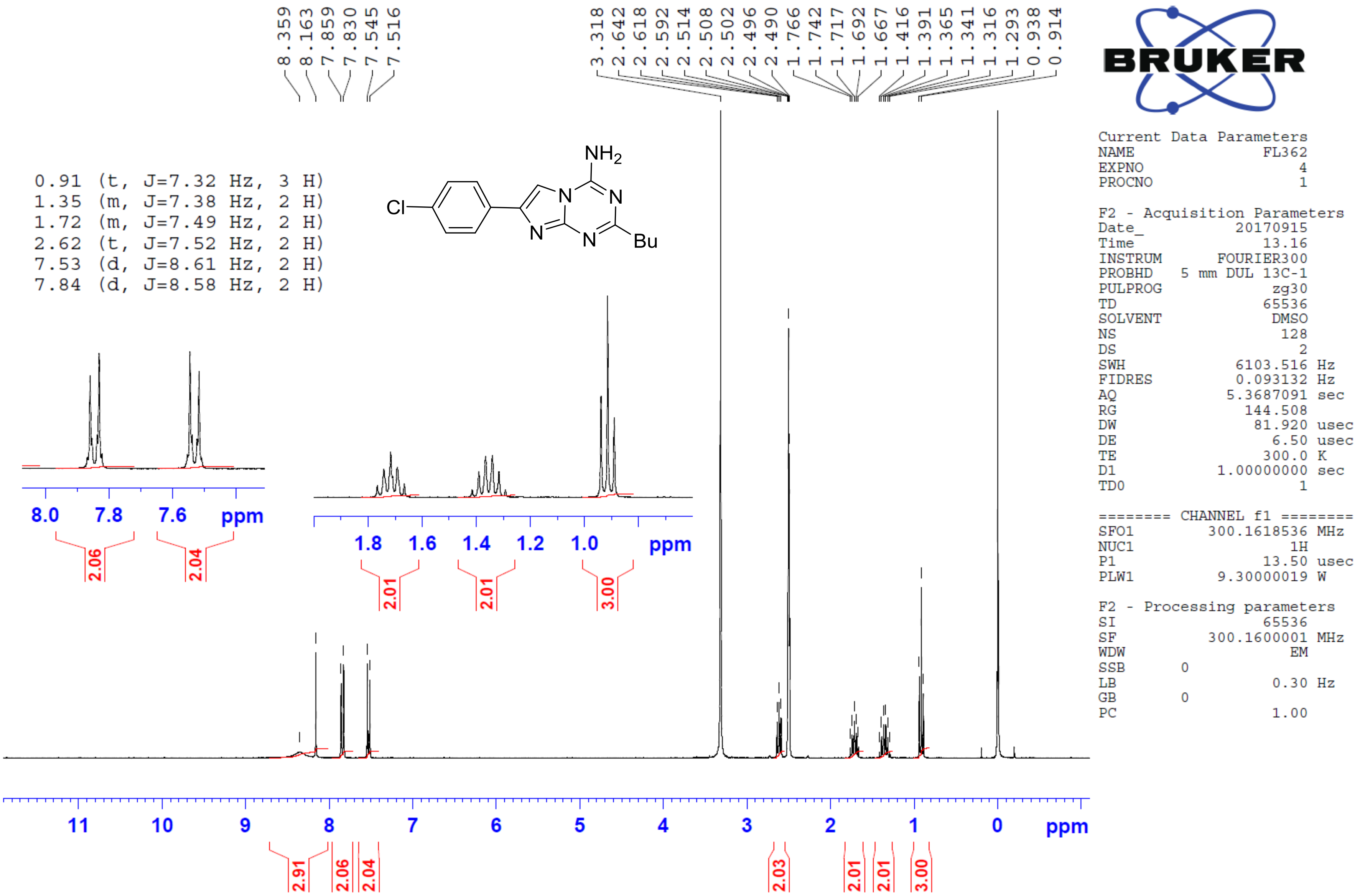

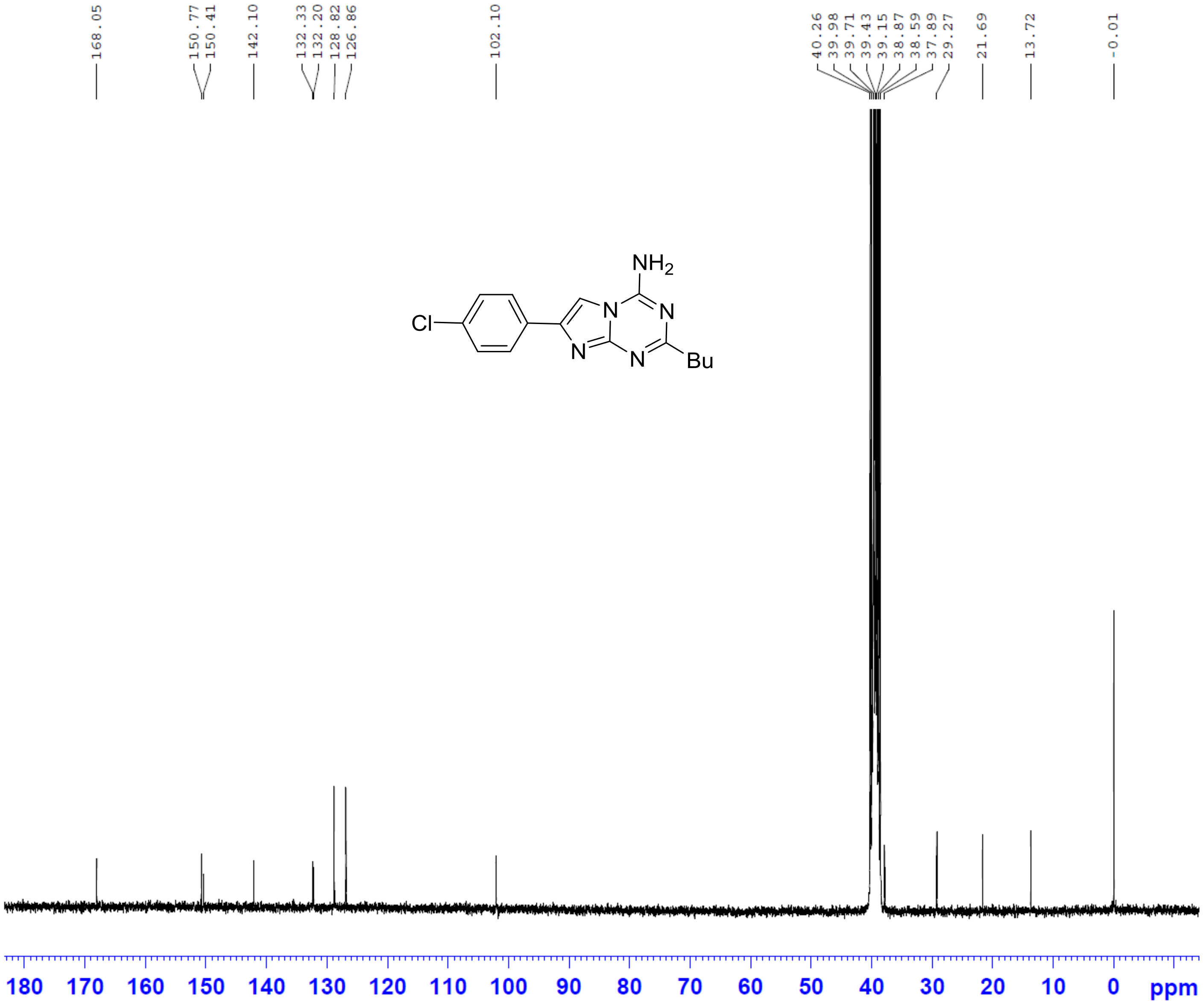


```
Current Data Parameters
NAME              FL362
EXPNO                 5
PROCNO                1

F2 - Acquisition Parameters
Date_          20170915
Time              18.10
INSTRUM     FOURIER300
PROBHD   5 mm DUL 13C-1
PULPROG         zgpg30
TD                65536
SOLVENT            DMSO
NS                40960
DS                    4
SWH           24414.063 Hz
FIDRES         0.372529 Hz
AQ            1.3421773 sec
RG              501.187
DW               20.480 usec
DE                 6.50 usec
TE                300.0 K
D1           2.00000000 sec
D11          0.03000000 sec
D31          0.00001500 sec
D40          0.00439029 sec
L4                   37
L5                   53
P32               98.00 usec
TD0                  40

======== CHANNEL f1 ========
SFO1         75.4828392 MHz
NUC1                13C
P1                15.00 usec
PLW1        22.00000000 W

======== CHANNEL f2 ========
SFO2        300.1612006 MHz
NUC2                 1H
CPDPRG[2        waltz16
PCPD2             98.00 usec
PLW2         9.30000019 W
PLW12        0.29359001 W
PLW13        0.20359001 W

F2 - Processing parameters
SI                32768
SF           75.4754001 MHz
WDW                  EM
SSB      0
LB                 1.00 Hz
GB       0
PC                 1.40
```

4-Amino-2-butyl-7-(4-methylphenyl)imidazo[1,2-a][1,3,5]triazine (5v)

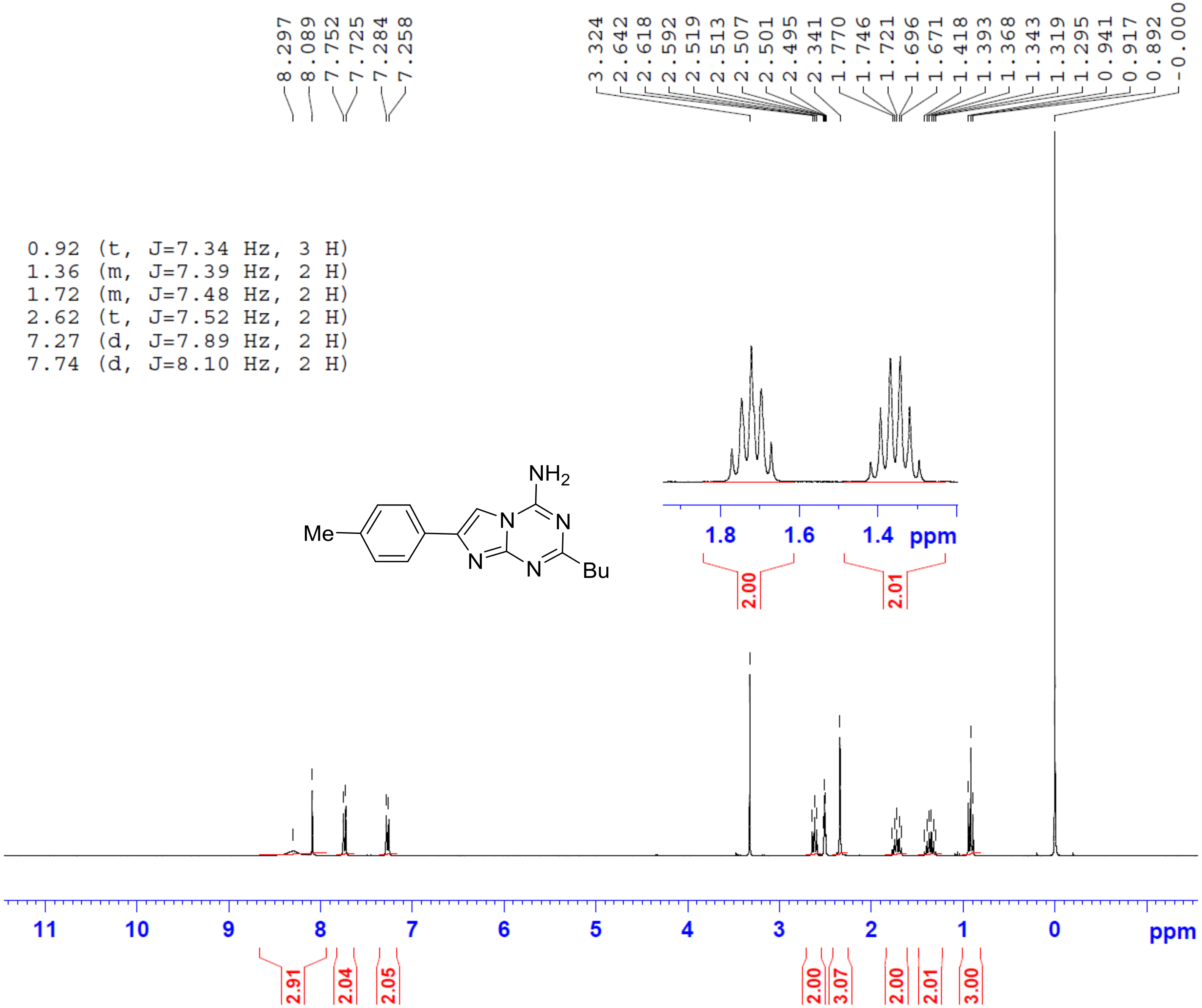

0.92 (t, J=7.34 Hz, 3 H)
1.36 (m, J=7.39 Hz, 2 H)
1.72 (m, J=7.48 Hz, 2 H)
2.62 (t, J=7.52 Hz, 2 H)
7.27 (d, J=7.89 Hz, 2 H)
7.74 (d, J=8.10 Hz, 2 H)

BRUKER

```
Current Data Parameters
NAME                FL368
EXPNO                   2
PROCNO                  1

F2 - Acquisition Parameters
Date_            20170924
Time                16.04
INSTRUM        FOURIER300
PROBHD    5 mm DUL 13C-1
PULPROG              zg30
TD                  65536
SOLVENT              DMSO
NS                     16
DS                      2
SWH              6103.516 Hz
FIDRES           0.093132 Hz
AQ              5.3687091 sec
RG                82.2225
DW                 81.920 usec
DE                   6.50 usec
TE                  300.0 K
D1             1.00000000 sec
TD0                     1

======== CHANNEL f1 ========
SFO1          300.1618536 MHz
NUC1                   1H
P1                  13.50 usec
PLW1           9.30000019 W

F2 - Processing parameters
SI                  65536
SF            300.1599989 MHz
WDW                    EM
SSB      0
LB                   0.30 Hz
GB       0
PC                   1.00
```

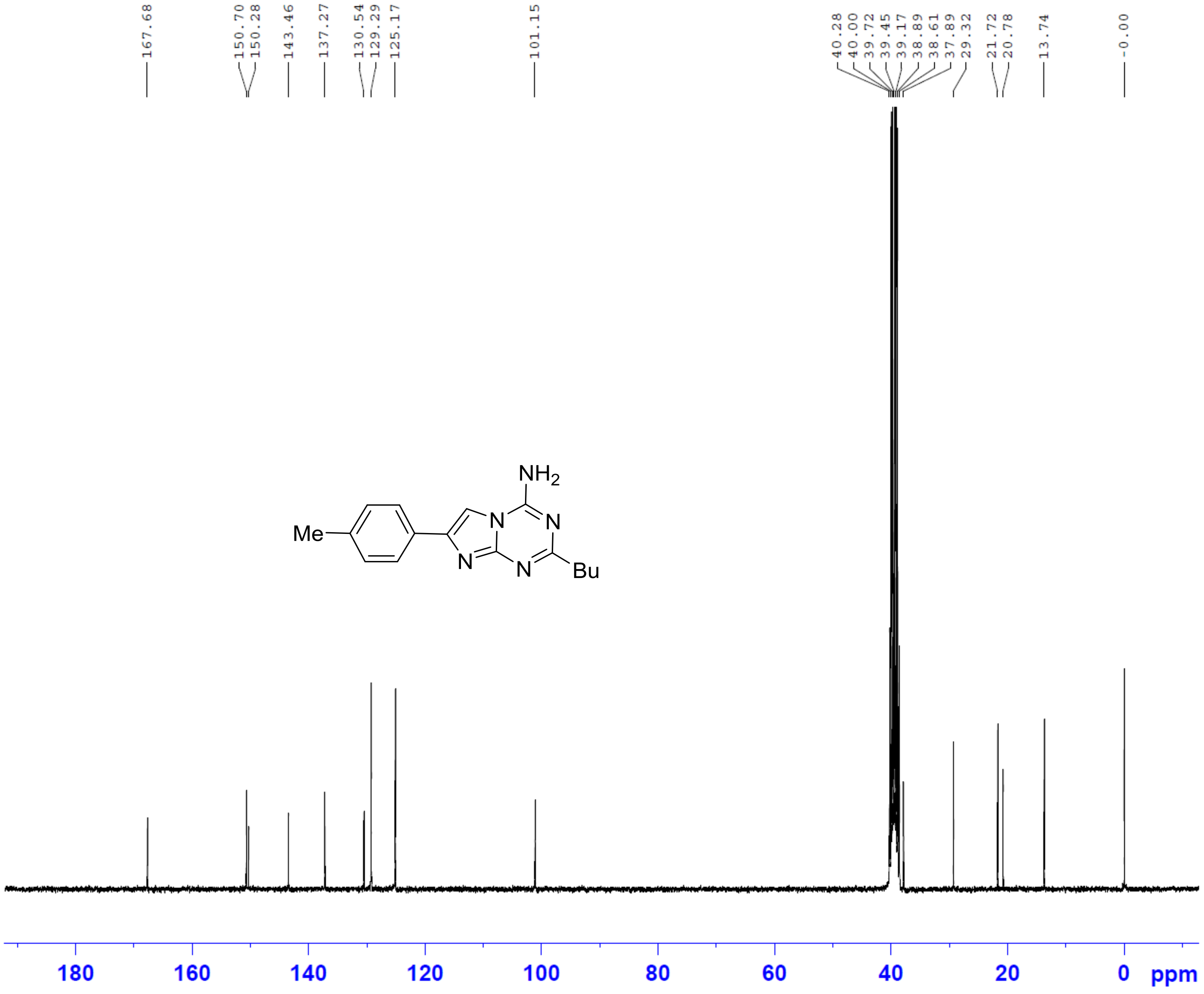


```
Current Data Parameters
NAME               FL368
EXPNO                  3
PROCNO                 1

F2 - Acquisition Parameters
Date_           20170925
Time               18.09
INSTRUM       FOURIER300
PROBHD   5 mm DUL 13C-1
PULPROG          zgpg30
TD                 65536
SOLVENT             DMSO
NS                 13312
DS                     4
SWH            24414.063 Hz
FIDRES          0.372529 Hz
AQ             1.3421773 sec
RG               501.187
DW                20.480 usec
DE                  6.50 usec
TE                 300.1 K
D1            2.00000000 sec
D11           0.03000000 sec
D31           0.00001500 sec
D40           0.00439029 sec
L4                    37
L5                    53
P32                98.00 usec
TD0                   13

======== CHANNEL f1 ========
SFO1          75.4828392 MHz
NUC1                 13C
P1                 15.00 usec
PLW1         22.00000000 W

======== CHANNEL f2 ========
SFO2         300.1612006 MHz
NUC2                  1H
CPDPRG[2         waltz16
PCPD2              98.00 usec
PLW2          9.30000019 W
PLW12         0.29359001 W
PLW13         0.20359001 W

F2 - Processing parameters
SI                 32768
SF            75.4753339 MHz
WDW                   EM
SSB      0
LB                  1.00 Hz
GB       0
PC                  1.40
```

4-Amino-2-butyl-7-(4-methoxyphenyl)imidazo[1,2-a][1,3,5]triazine (5w)

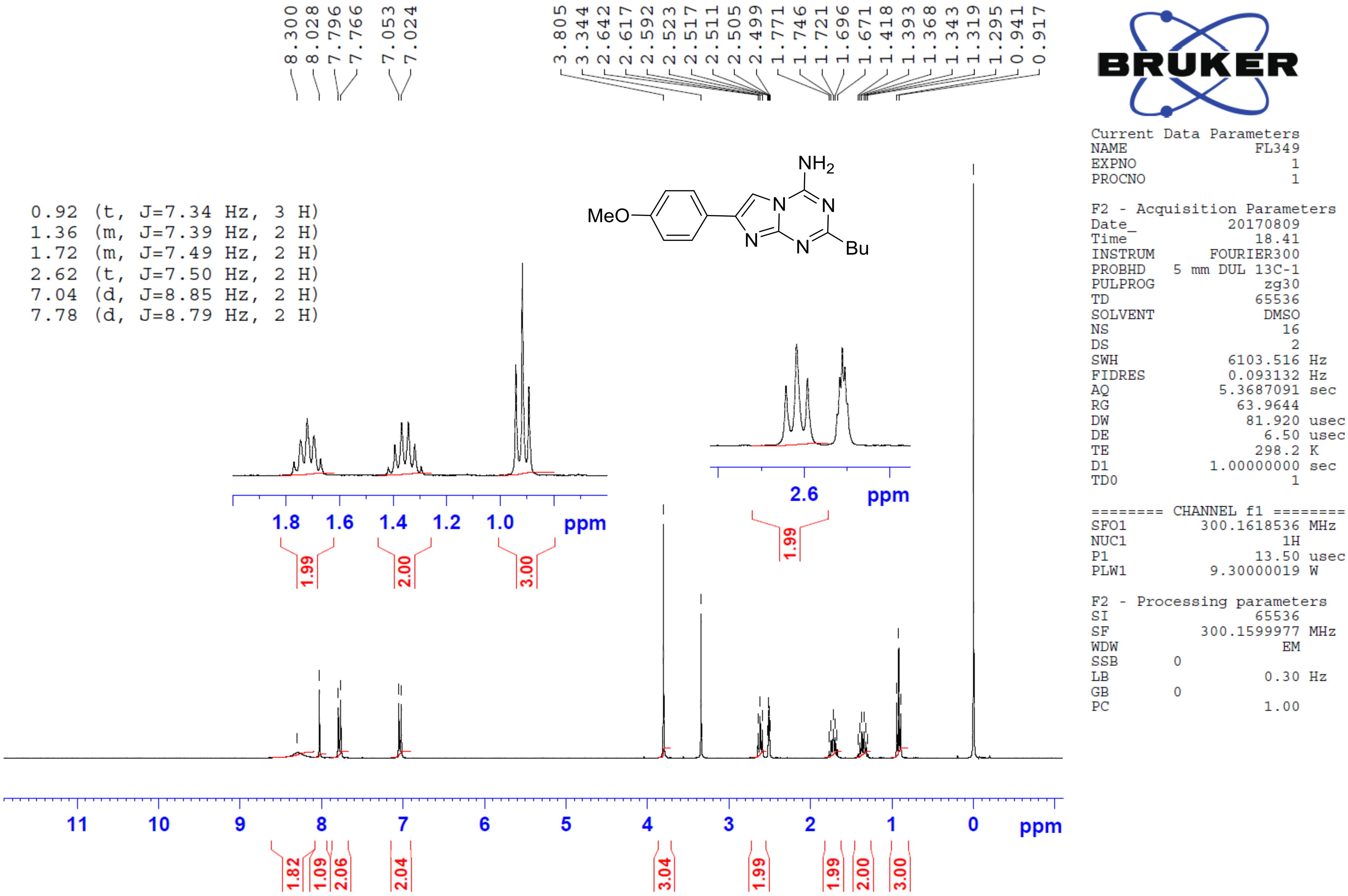

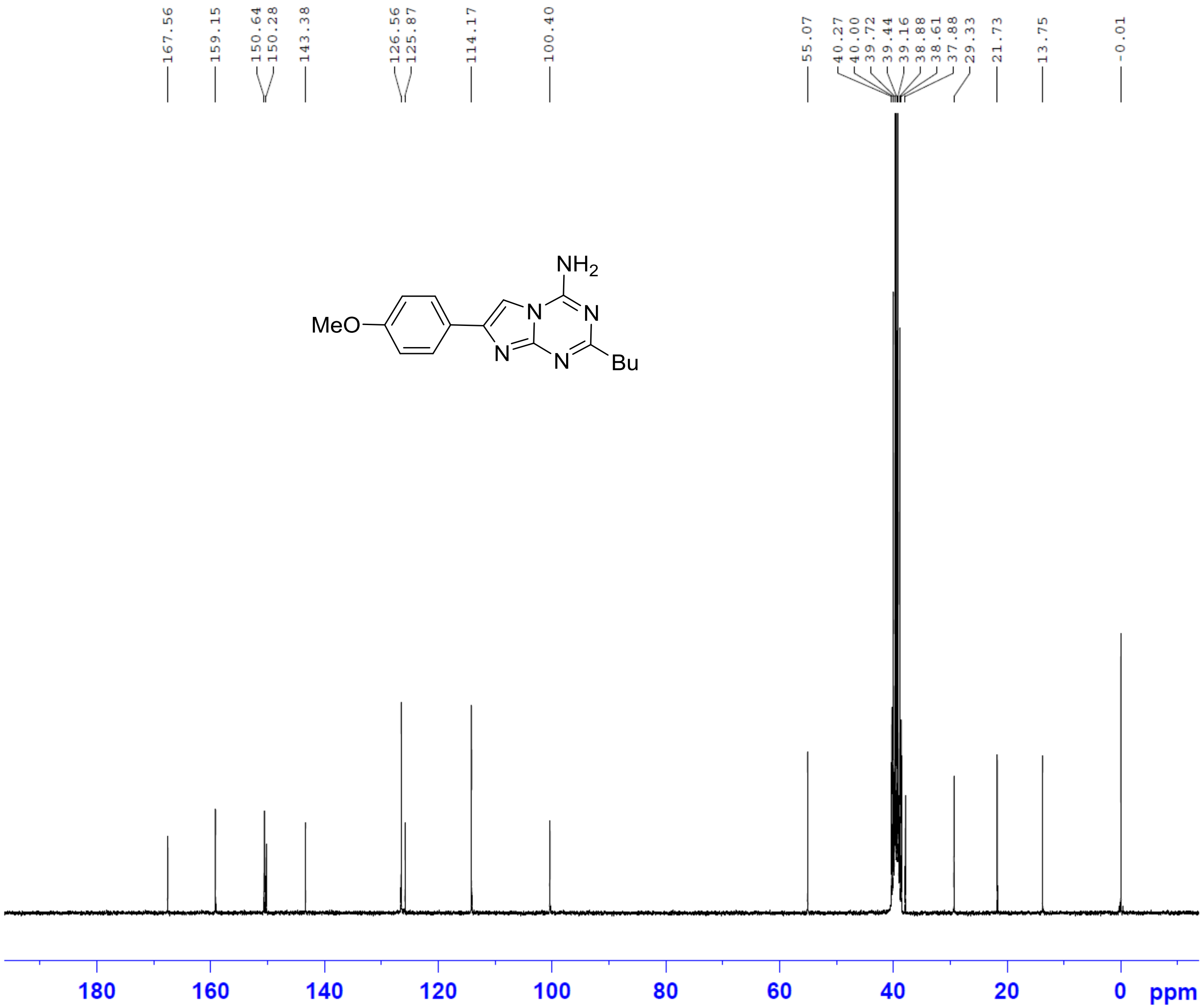

Current Data Parameters
NAME FL349
EXPNO 2
PROCNO 1

F2 - Acquisition Parameters
Date_ 20170809
Time 18.59
INSTRUM FOURIER300
PROBHD 5 mm DUL 13C-1
PULPROG zgpg30
TD 65536
SOLVENT DMSO
NS 14336
DS 4
SWH 24414.063 Hz
FIDRES 0.372529 Hz
AQ 1.3421773 sec
RG 501.187
DW 20.480 usec
DE 6.50 usec
TE 298.2 K
D1 2.00000000 sec
D11 0.03000000 sec
D31 0.00001500 sec
D40 0.00439029 sec
L4 37
L5 53
P32 98.00 usec
TD0 14

======== CHANNEL f1 ========
SFO1 75.4828392 MHz
NUC1 13C
P1 15.00 usec
PLW1 22.00000000 W

======== CHANNEL f2 ========
SFO2 300.1612006 MHz
NUC2 1H
CPDPRG[2 waltz16
PCPD2 98.00 usec
PLW2 9.30000019 W
PLW12 0.29359001 W
PLW13 0.20359001 W

F2 - Processing parameters
SI 32768
SF 75.4753335 MHz
WDW EM
SSB 0
LB 1.00 Hz
GB 0
PC 1.40

4-Amino-2-butyl-7-(4-biphenyl)imidazo[1,2-a][1,3,5]triazine (5x)

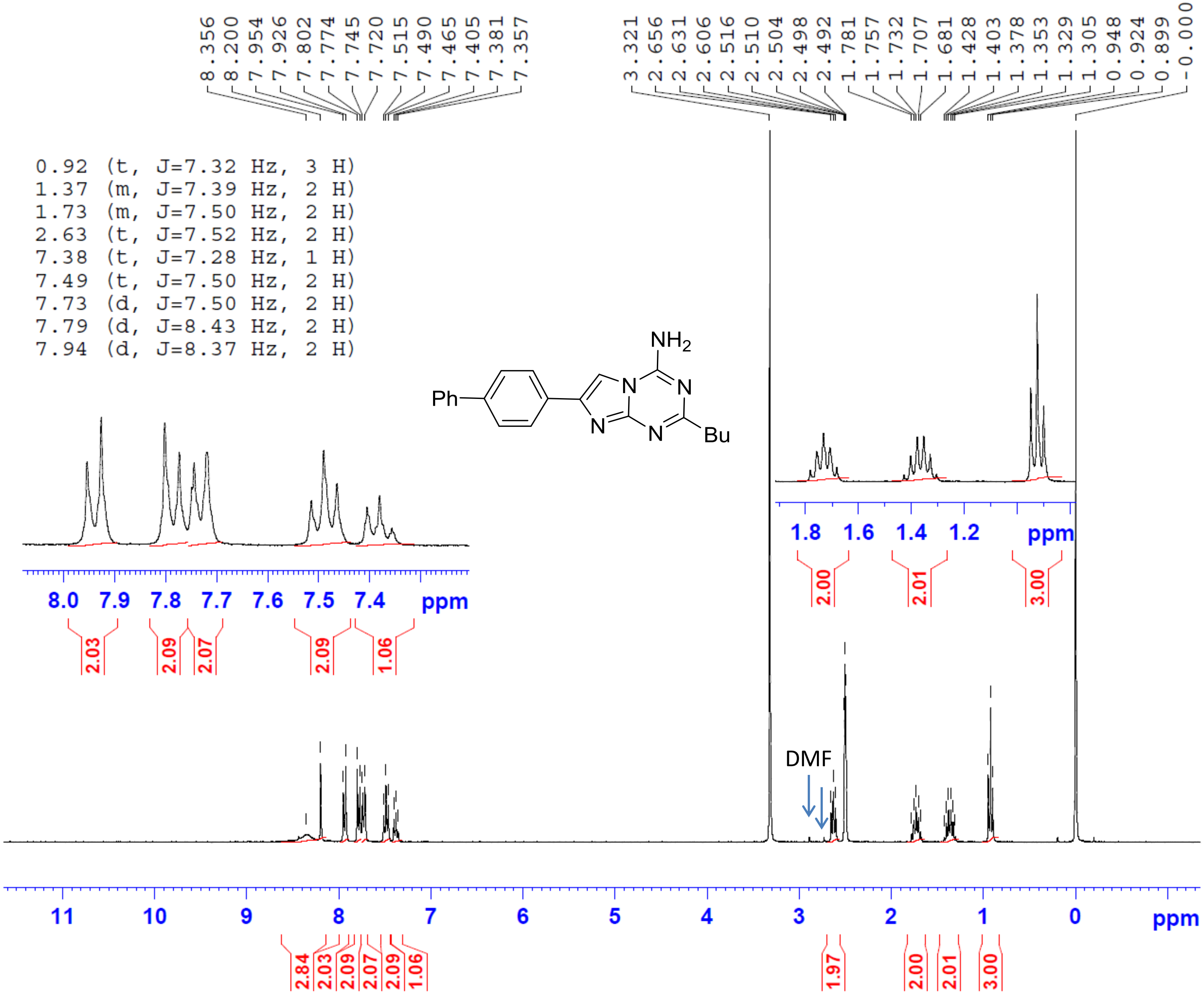


```
0.92 (t, J=7.32 Hz, 3 H)
1.37 (m, J=7.39 Hz, 2 H)
1.73 (m, J=7.50 Hz, 2 H)
2.63 (t, J=7.52 Hz, 2 H)
7.38 (t, J=7.28 Hz, 1 H)
7.49 (t, J=7.50 Hz, 2 H)
7.73 (d, J=7.50 Hz, 2 H)
7.79 (d, J=8.43 Hz, 2 H)
7.94 (d, J=8.37 Hz, 2 H)
```

```
Current Data Parameters
NAME              FL375
EXPNO                 3
PROCNO                1

F2 - Acquisition Parameters
Date_          20171023
Time              17.05
INSTRUM      FOURIER300
PROBHD   5 mm DUL 13C-1
PULPROG            zg30
TD                65536
SOLVENT            DMSO
NS                   16
DS                    2
SWH            6103.516 Hz
FIDRES         0.093132 Hz
AQ            5.3687091 sec
RG              147.582
DW               81.920 usec
DE                 6.50 usec
TE                300.1 K
D1           1.00000000 sec
TD0                   1

======== CHANNEL f1 ========
SFO1        300.1618536 MHz
NUC1                 1H
P1                13.50 usec
PLW1         9.30000019 W

F2 - Processing parameters
SI                65536
SF          300.1599999 MHz
WDW                  EM
SSB        0
LB                 0.30 Hz
GB         0
PC                 1.00
```

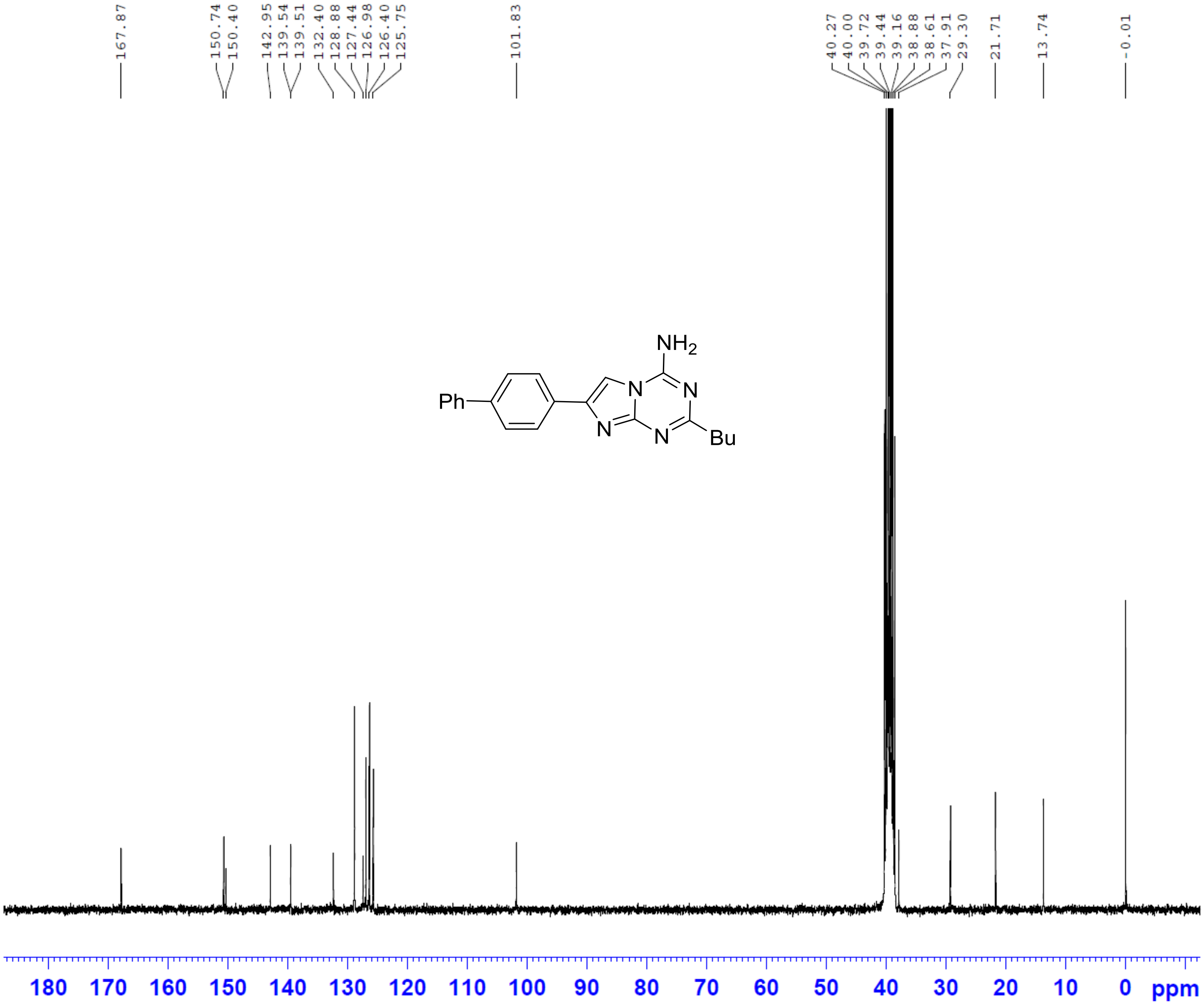

Current Data Parameters

NAME	FL375
EXPNO	2
PROCNO	1

F2 - Acquisition Parameters

Date_	20171022
Time	14.10
INSTRUM	FOURIER300
PROBHD	5 mm DUL 13C-1
PULPROG	zgpg30
TD	65536
SOLVENT	DMSO
NS	20480
DS	4
SWH	24414.063 Hz
FIDRES	0.372529 Hz
AQ	1.3421773 sec
RG	501.187
DW	20.480 usec
DE	6.50 usec
TE	300.0 K
D1	2.00000000 sec
D11	0.03000000 sec
D31	0.00001500 sec
D40	0.00439029 sec
L4	37
L5	53
P32	98.00 usec
TD0	20

======== CHANNEL f1 ========

SFO1	75.4828392 MHz
NUC1	13C
P1	15.00 usec
PLW1	22.00000000 W

======== CHANNEL f2 ========

SFO2	300.1612006 MHz
NUC2	1H
CPDPRG[2	waltz16
PCPD2	98.00 usec
PLW2	9.30000019 W
PLW12	0.29359001 W
PLW13	0.20359001 W

F2 - Processing parameters

SI	32768
SF	75.4753350 MHz
WDW	EM
SSB	0
LB	1.00 Hz
GB	0
PC	1.40

_N'_,_N'_-morpholino-_N_-[3(5)-phenylimidazolo-5(3)yl acetamidine _(6)_

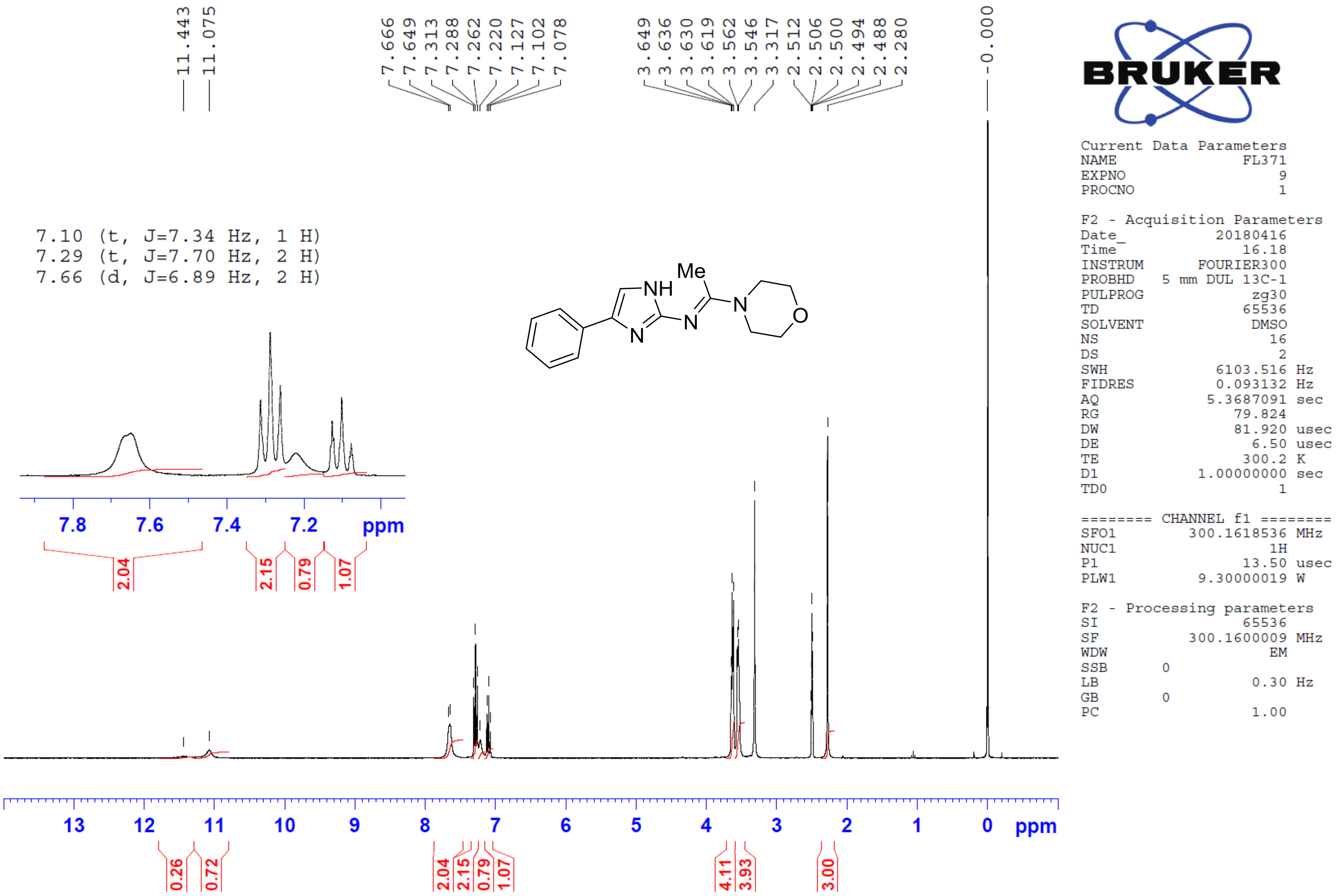

X-Ray crystallographic data for **5g** and **5p**

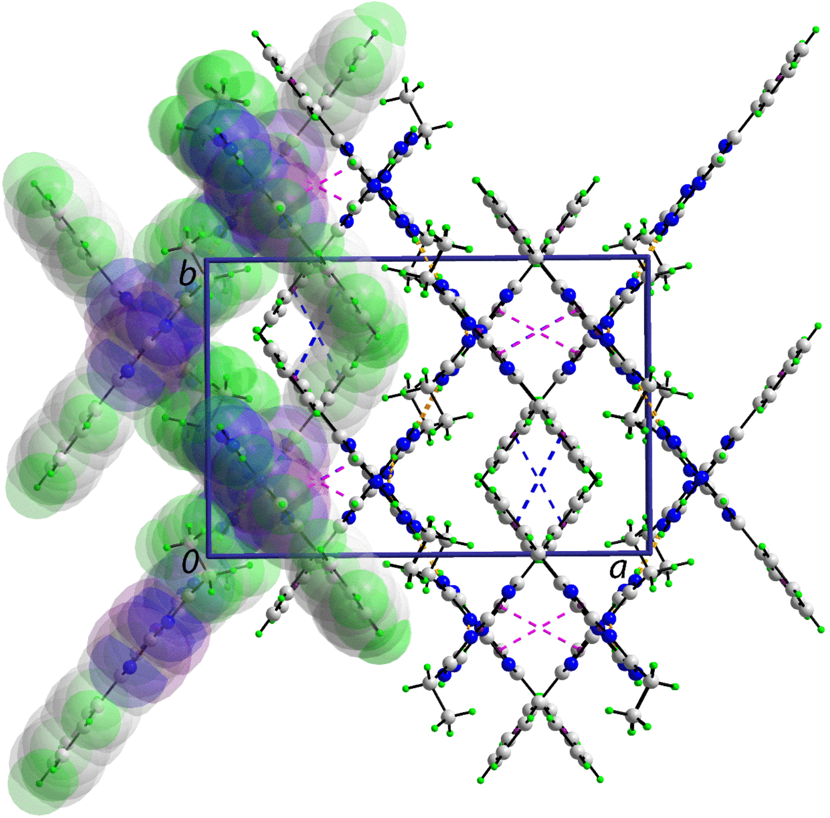

ESI Figure S1. A view in projection down the *c*-axis of the unit cell contents for **5g**. One supramolecular layer is highlighted in space-filling mode. The N–H···N, π(imidazo)···π(imidazo) and π(phenyl)···π(phenyl) interactions are shown as orange, pink and blue dashed lines, respectively. Geometric parameters characterising the identified intermolecular interactions. N–H···N: N41–H4n1···N3^{i} = 2.088(11) Å, N41···N3^{i} = 2.9669(13) Å and angle at H4n1 = 174.5(11)°; N41–H4n2···N1^{ii} = 2.064(12) Å, N41···N1^{ii} = 2.9277(12) Å and angle at H4n2 = 163.9(11)°. π(imidazo)···π(imidazo): Cg(N5,C6,C7,N8,C9)···Cg(N5,C6,C7,N8,C9)^{iii} = 3.4699(6) Å and angle of inclination = 5.41(6)°. π(phenyl)···π(phenyl): Cg(C71-C76)···Cg(C71-C76)^{iv} = 3.8142(7) Å and angle of inclination = 6.53(5)°. Symmetry operations: (i) 1-*x*, 1-*y*, 1-*z*; (ii) *x*, ½-*y*, ½+*z*; (iii) ½-*x*, ½-*y*, *z*; (iv) ½-*x*, -½-*y*, *z*.

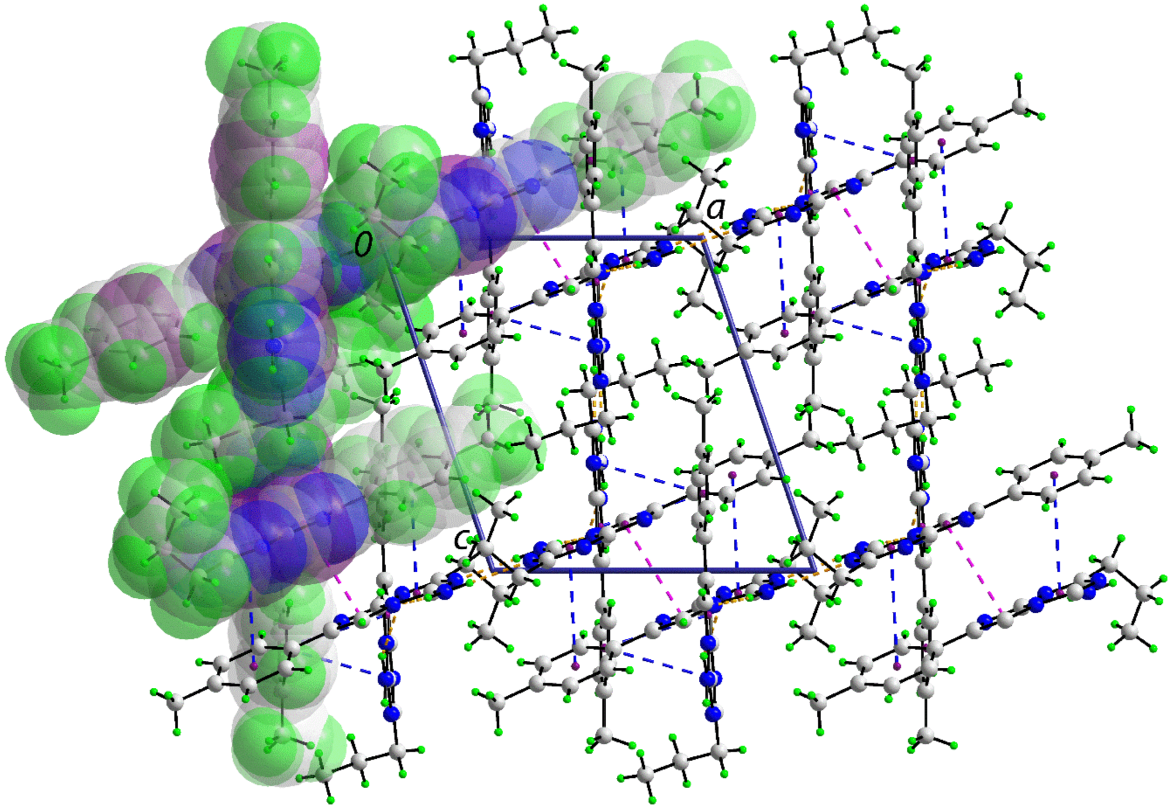

ESI Figure S2. A view in projection down the *b*-axis of the unit cell contents for **5p**. One supramolecular layer is highlighted in space-filling mode. The N–H⋯N, p(imidazo)⋯p(imidazo) and p(triazine)⋯p(phenyl) interactions are shown as orange, pink and blue dashed lines, respectively. Geometric parameters characterising the identified intermolecular interactions. N–H⋯N: N41–H4n1⋯N3^{i} = 2.171(12) Å, N41⋯N3^{i} = 3.0501(16) Å and angle at H4n1 = 179.9(18)°; N41–H4n2⋯N8a^{ii} = 2.192(13) Å, N41⋯N8a^{ii} = 3.0636(15) Å and angle at H4n2 = 173.5(13)°; N41a–H4n3⋯N3a^{iii} = 2.081(12) Å, N41a⋯N3a^{iii} = 2.9561(14) Å and angle at H4n3 = 176.6(13)°; N41a–H4n4⋯N1^{iii} = 2.501(12) Å, N41a⋯N1^{iii} = 3.3320(15) Å and angle at H4n4 = 156.8(15)°. p(imidazo)⋯p(imidazo): Cg(N5,C6,C7,N8,C9)⋯Cg(N5,C6,C7,N8,C9)^{iv} = 3.5695(7) Å and angle of inclination = 0°. p(triazine)⋯p(phenyl): Cg(N1,N3,N5,C2,C4,C9)⋯Cg(C71-C76)^{iv} = 3.8946(7) Å and angle of inclination = 18.43(6)°; Cg(N1a,N3a,N5a,C2a,C4a,C9a)⋯Cg(C71a-C76a)^{v} = 3.9224(7) Å and angle of inclination = 10.30(6)°. Symmetry operations: (i) -*x*, -*y*, 2-*z*; (ii) 1-*x*, -*y*, 1-*z*; (iii) 1-*x*, 1-*y*, 1-*z*; (iv) 1-*x*, -*y*, 2-*z*; (v) 1-*x*, 1-*y*, -*z*.
